# Clinical relevance of biomarkers, new therapeutic approaches, and role of post-translational modifications in the pathogenesis of Alzheimer’s disease

**DOI:** 10.3389/fnagi.2022.977411

**Published:** 2022-09-07

**Authors:** Ibtisam Mumtaz, Mir Owais Ayaz, Mohamad Sultan Khan, Umar Manzoor, Mohd Azhardin Ganayee, Aadil Qadir Bhat, Ghulam Hassan Dar, Badrah S. Alghamdi, Anwar M. Hashem, Mohd Jamal Dar, Gulam Md. Ashraf, Tariq Maqbool

**Affiliations:** ^1^Laboratory of Nanotherapeutics and Regenerative Medicine, Department of Nanotechnology, University of Kashmir, Srinagar, India; ^2^Laboratory of Cell and Molecular Biology, Department of Cancer Pharmacology, CSIR-Indian Institute of Integrative Medicine, Jammu, India; ^3^Centre for Scientific and Innovative Research, Ghaziabad, Utter Pradesh, India; ^4^Neurobiology and Molecular Chronobiology Laboratory, Department of Animal Biology, School of Life Sciences, University of Hyderabad, Hyderabad, India; ^5^Laboratory of Immune and Inflammatory Disease, Jeju Research Institute of Pharmaceutical Sciences, Jeju National University, Jeju, South Korea; ^6^Department of Chemistry, Indian Institute of Technology Madras, Chennai, India; ^7^Sri Pratap College, Cluster University Srinagar, Jammu and Kashmir, India; ^8^Department of Physiology, Neuroscience Unit, Faculty of Medicine, King Abdulaziz University, Jeddah, Saudi Arabia; ^9^Pre-clinical Research Unit, King Fahd Medical Research Center, King Abdulaziz University, Jeddah, Saudi Arabia; ^10^Department of Medical Microbiology and Parasitology, Faculty of Medicine, King Abdulaziz University, Jeddah, Saudi Arabia; ^11^Vaccines and Immunotherapy Unit, King Fahd Medical Research Center, King Abdulaziz University, Jeddah, Saudi Arabia; ^12^Department of Medical Laboratory Sciences, Faculty of Applied Medical Sciences, King Abdulaziz University, Jeddah, Saudi Arabia

**Keywords:** Alzheimer’s disease, AD-related proteins, biomarkers, post translational modifications, AD therapeutics

## Abstract

Alzheimer’s disease (AD) is a neurodegenerative disorder that causes progressive loss of cognitive functions like thinking, memory, reasoning, behavioral abilities, and social skills thus affecting the ability of a person to perform normal daily functions independently. There is no definitive cure for this disease, and treatment options available for the management of the disease are not very effective as well. Based on histopathology, AD is characterized by the accumulation of insoluble deposits of amyloid beta (Aβ) plaques and neurofibrillary tangles (NFTs). Although several molecular events contribute to the formation of these insoluble deposits, the aberrant post-translational modifications (PTMs) of AD-related proteins (like APP, Aβ, tau, and BACE1) are also known to be involved in the onset and progression of this disease. However, early diagnosis of the disease as well as the development of effective therapeutic approaches is impeded by lack of proper clinical biomarkers. In this review, we summarized the current status and clinical relevance of biomarkers from cerebrospinal fluid (CSF), blood and extracellular vesicles involved in onset and progression of AD. Moreover, we highlight the effects of several PTMs on the AD-related proteins, and provide an insight how these modifications impact the structure and function of proteins leading to AD pathology. Finally, for disease-modifying therapeutics, novel approaches, and targets are discussed for the successful treatment and management of AD.

## Introduction

Alzheimer’s disease (AD) is a neurodegenerative disorder associated with diminished regenerative capacity of neurons and impaired cognitive functions including learning and memory (Galimberti and Scarpini, 2012; Haque and Levey, 2019; Chatterjee et al., 2020). Nearly 35 million people are suffering from AD worldwide and is estimated to be doubled by 2030 (Alzheimer’s Association, 2019; Haque and Levey, 2019). The nature of this disease demands proper and long-term medical care which has accounted for an estimated cost of $195 billion in 2019 and is expected to rise to $1 trillion by 2050 (Alzheimer’s Association, 2019). Various factors contribute to AD-related dementia and impairment of cognitive functions, however, extracellular amyloid beta (Aβ) plaques and intracellular aggregates of hyperphosphorylated tau proteins also called neurofibrillary tangles (NFTs) are the two major histopathological hallmarks of AD (Mayeux and Stern, 2012; Xu et al., 2012; Kametani and Hasegawa, 2018; Janeiro et al., 2021). Accumulation of amyloid beta (Aβ) plaques and NFTs initiate a cascade of events, resulting firstly in synaptic dysfunction, axonal degeneration and impaired cellular communication, and followed subsequently as the disease progresses by gliosis, neurodegeneration and widespread neuronal death (Wang et al., 2013; Hampel et al., 2015; Li et al., 2015; Pini et al., 2016; Knezevic et al., 2018). Although accumulation of Aβ deposits and NFTs are the pathological hallmarks of AD and have drawn the special attention of researchers in the search for biological markers, it is clear now that the disease begins decades before the onset of any clinical symptoms. To predict, diagnose, or monitor the progression of AD disease biomarkers are considered useful in every step of patient care. As disease symptoms are subjective, biomarkers provide an objective, measurable way to characterize the disease. Biomarkers for Alzheimer’s disease aim to facilitate early disease prognosis and makes it possible to determine the progression of disease during initial stage and evaluate response to existing and future treatments. Also, biomarkers are likely to predict clinical benefit and support accelerated or traditional drug approval, respectively. There is an unmet need for identification of such clinical biomarker for Alzheimer’s disease.

## Major proteins and enzymes involved in Alzheimer’s progression: Role of amyloid-beta precursor protein, secretase, Tau, beta-site amyloid-beta precursor protein-cleaving enzyme, Apo E, PS1/2, and microglia

### Amyloid-beta precursor protein and secretase enzymes

Amyloid-beta precursor protein (APP) encoded by a gene *APP* (located on chromosome 21) is a ubiquitous type-1 transmembrane protein with three splice variants: APP695, APP751, and APP770 expressed mostly in neurons, astrocytes, and vascular endothelial cells respectively (Miura et al., 2020; Zhao et al., 2020). Although the exact functions of APP are not known, however, its expression increases during differentiation of neurons and synapse formation, and declines once mature connections are established, suggesting the role of APP in aging and development (Chen et al., 2017). Under normal conditions, APP undergoes non-pathogenic processing by involving two important enzymes α-and γ-secretase. These enzymes cleave the APP within the Aβ domain resulting into non-amyloidogenic fragments along with soluble amyloid precursor protein fragments α (sAPPα) and C-terminal fragments (CTFs). In diseased conditions, a different set of enzymes including β-and γ-secretase to cleave the APP in such a way that it generates the neurotoxic Aβ peptides (40–42 amino acid long peptides), along with soluble amyloid precursor protein fragments β (sAPPβ) and CTFs (Nesterova et al., 2019). Various studies have shown a link between the mutations of *APP* and AD; for example 10–15% cases of early-onset-familial Alzheimer’s disease (EOFAD) are reported to be caused by APP gene mutations (Hooli and Tanzi, 2016). Such mutations appear to influence the biology of Aβ by promoting local oligomer/fibril formation or changing the propensity of Aβ to bind to other proteins and affecting Aβ clearance. This ability of APP to undergo cleavage using different set of enzymes to form either soluble or pathogenic amyloid-beta (Aβ) peptides makes APP as a main target protein in AD progression. Any mutation in APP and in the proteins that regulate APP endocytosis and processing in neurons leading to disturbed APP-related intracellular signaling pathways can be used as a biomarker during early stages of AD.

### Tau protein

Tau is a microtubule-associated protein involved in the stabilization of microtubules by promoting their polymerization. The association of tau protein with the microtubules is involved in regulating the axonal transport as well as neuronal cytoskeleton (Zhou et al., 2018). Six different isoforms of tau are usually expressed in normal mature human brains however they are found to be abnormally hyperphosphorylated in AD brains. Any unusual alterations in the structural conformation or phosphorylation events of tau impact its binding affinity with microtubule which leads to its toxic aggregation in the form of neurofibrillary tangles (NFTs) and paired helical filaments (PHFs). These NFTs which on aggregation attain the shape of PHFs are one among the major hallmarks seen in pathology of AD (Augustinack et al., 2002). Tau phosphorylation and its detailed role in the pathogenesis is included elsewhere under post-translational modifications (PTMs) in AD. Importantly, Hyper-phosphorylated tau is considered to be promising biomarker for monitoring the disease progression in AD.

### Beta-site amyloid-beta precursor protein-cleaving enzyme

Beta-Site APP-cleaving enzyme (BACE) is a ubiquitously expressed membrane-bound aspartyl protease. It uses its proteolytic activity for the production of neuro-pathogenic Aβ peptides. There are four splice variants of BACE with 501, 476, 457, and 432 amino acids. Among these variants 501 variant is having the highest degree of proteolytic activity on Aβ amyloid substrate (Mowrer and Wolfe, 2008). The production of Aβ peptides from its precursor APP occurs in two sequential proteolytic cleavages. First cleavage is catalyzed by BACE in which BACE cleaves the ectodomain of APP generating a C99 membrane-bound C-terminal fragment and the second proteolytic reaction is carried out by γ-secretase which further processes a C99 fragment leading to the formation of Aβ peptides (Bolduc et al., 2016). In AD patients BACE is highly expressed in various parts of the brain especially in brain cortex and cerebrospinal fluid (CSF) (Hampel et al., 2020). The increased expression of BACE can serve as an early biomarker in detection of AD (Blennow et al., 2010; Evin et al., 2010), and its increased expression has been directly co-related with the age of the patient and stress level (O’brien and Wong, 2011). BACE1 protein concentrations and rates of enzyme activity are promising candidates among biological markers in clinical trials investigating the role of BACE1 inhibitors in regulating APP processing.

### Apolipoprotein E

In the central nervous system (CNS), apolipoprotein E (ApoE) is mostly synthesized and produced by astrocytes to transport cholesterol to neurons *via* ApoE receptors (Bu, 2009). ApoE is composed of 299 amino acids and exists in three isoforms in humans; ApoE2, ApoE3, and ApoE4. The single amino acid differences alter the structure of these isoforms and influences their functional abilities (Frieden and Garai, 2012). The ApoE4 isoform represents the most significant risk factor for late-onset Alzheimer disease (LOAD) (Corder et al., 1993; Mahoney-Sanchez et al., 2016). The individuals carrying the rare E2 variant are less likely to develop AD and E3 represents the most common but non-pathogenic isoform of ApoE (Serrano-Pozo et al., 2015). Although number of studies have been conducted to understand the mechanism of action of different variants of ApoE, but further investigation and research is needed to fully understand the differential effects of ApoE isoforms on Aβ aggregation and clearance in AD pathogenesis (Kim et al., 2009; Husain et al., 2021). In an interesting study, the total ApoE and ApoE4 plasma proteins were assessed using Australian Imaging, Biomarkers and Lifestyle (AIBL) study of aging and the result were assessed by Positron Emission Tomography (PET) using Pittsburgh compound B (PiB). The levels of these plasma proteins were compared with cerebral Aβ load, and it was found that both the total ApoE as well as ApoE4 levels are significantly lowered in AD patients. From ApoE genotyping, the protein (ApoE) levels were significantly lower among E4 homozygous individuals and in *APOE* E3/E4 heterozygote carriers, ApoE4 levels decreased, indicating that ApoE3 levels increase with disease. This study suggests that ApoE, ApoE3, and ApoE4 can be used as AD biomarkers and possible therapeutic drug targets (Gupta et al., 2011; Soares et al., 2012).

### Presenilin 1

Presenilin 1 protein encoded by *PSEN1* gene is located on chromosome 14 and forms an important component of the γ-secretase complex, which cleaves APP into Aβ fragments (Steiner et al., 2008). It is mostly expressed in endoplasmic reticulum and helps in protein processing (Bezprozvanny and Mattson, 2008). The importance of PSEN1 gene in AD is evident from the fact that it accounts for about 50% cases of early-onset Alzheimer’s disease (EOAD), with complete penetrance (Giri et al., 2016). Mutation in PSEN1 leads to mutations in γ-secretase and increase in the Aβ42/40 ratio resulting in cotton wool plague formation (Zhang et al., 2015; Miki et al., 2019). Only few mutations are insertions and deletions, majority of *PSEN1* mutations are missense. PSEN1 mutations not only affect the activity of γ-secretase enzyme but also directly affect the neuronal functioning by controlling the activity of GSK-3β and kinesin I (Giri et al., 2016). More than 295 pathogenic mutations have been identified in *PSEN1*, of which 70% mutations occur in exons 5, 6, 7, and 8. Studies using genetically modified mice have shown that mutations in PSEN1 lead to impaired Aβ production and increased ratio of Aβ42/Aβ40 (Xia et al., 2015). Recent studies that investigated potential relationships between the molecular composition of FAD-linked Aβ profiles and disease severity by analyzing Aβ profiles generated by 25 mutant PSEN1/GSECs that span a wide range of AAOs, revealed that full spectrum of Aβ profiles (including Aβ37, Aβ38, Aβ40, Aβ42, and Aβ43) better reflects mutation pathogenicity. Furthermore, this study suggested Aβ(37 + 38 + 40)/Aβ(42 + 43) ratio better at predicting the age at disease onset (Petit et al., 2022). Presently, PSEN1 gene is considered as the most common cause of familial Alzheimer’s disease (FAD). However, a recent studies (Sun et al., 2017) contradicts the role of *PSEN1* in AD progression by increasing the Aβ42 production (Hardy and Selkoe, 2002) and therefore is a matter of a debate in the scientific world.

### Presenilin 2

Presenilin 2 protein encoded by *PSEN2* gene is located on chromosome 1 is similar in structure and function to *PSEN1* (Ridge et al., 2013). Similar to PSEN1, it also forms the important component of the γ secretase complex and any mutation in *PSEN2* alters the activity of γ secretase leading to elevated ratio of Aβ42/40 (Wakabayashi and De Strooper, 2008). Despite close homology between the two, mutations in PSEN2 are less toxic and less common than PSEN1, but neuritic plaque accumulation and neurofibrillary tangle (NFT) formation have been found in some people with *PSEN2* mutations (Giri et al., 2016). Efforts to develop disease-modifying therapies for AD have been heavily focused on the amyloid hypothesis but repeated failures in late-stage clinical trials based on these hypotheses heighten the urgency to explore alternative approaches. Thus, therapeutic strategies aimed at restoring secretase activities by involving modifications or mutations at the levels of PSN1 and PSN2 offer a valid and complementary approach to develop disease modifying treatments for FAD.

### Microglial role in Alzheimer’s disease

Microglia, a type of neuroglia (glial cells), forms the innate immune system of our central nervous system (CNS). Proliferation, activation, and concentration of these glial cells in the brain around amyloid plaques, is a prominent feature of AD. Data from human genetic studies also suggest the role of these cells in AD progression. Under normal conditions, microglia protect against AD, however, impaired microglial activities lead to increased risk of AD progression. Activated microglial cells can be harmful and mediate loss of synaptic junctions *via* complement-dependent mechanisms, increase tau phosphorylation and enhance inflammatory responses against neurons leading to activation of neurotoxic astrocytes (Hansen et al., 2018). The role of microglia in forming neuritic plaques was described long back by Alois Alzheimer himself (Alzheimer et al., 1995; Graeber et al., 1997) and further studies have shown the involvement of both reactive astrocytes and microglia in deposition of Aβ plagues (Verkhratsky et al., 2016). In AD patients, the microglia interact with the amyloid peptides, APP, and neurofibrillary tangles during early phase of AD, and their activation promote Aβ clearance through microglia’s scavenger receptors, and thus acts as a hurdle in the progression of AD. The Aβ activation induced continuous activation of microglia involving CD36, Fc receptors, toll-like receptors (TLRs), and complement receptors advanced glycation end products (RAGE), promote Aβ production while hampering Aβ clearance, which ultimately causes neuronal damage (Wang et al., 2015). In a study done on post-mortem brain sections taken from AD patient, it was found that increased microglia activation begins with amyloid NP deposition and the increase was found to be directly related to the part of brain involved in AD (Xiang et al., 2006). Thus, activation of microglia in brain tissues, such as hippocampi, can serve as an inflammatory biomarker for AD.

## Major sources and methods for isolation of potential biomarkers for Alzheimer’s disease

One of the major challenges in the treatment of Alzheimer’s disease is the lack of sensitive and specific biomarkers. Multiple studies have argued that AD begins decades before the onset of clinical symptoms and accumulation of Aβ deposits and NFTs-pathological hallmarks of AD. Clinically relevant biomarkers are expected to be useful in detecting the preclinical as well as symptomatic stages of AD. Such clinically relevant biomarkers used in the validation of AD are structured through a road map called the Strategic Biomarker Roadmap (SBR), initiated in 2017 and according to recent reports is still valid for the assessment of biomarkers of tauopathy, as well as that of the other diagnostic biomarkers of AD and related disorders (Boccardi et al., 2021). Moreover, biomarkers would be significantly helpful to predict, diagnose, or monitor the progression of AD disease during initial stage, and in evaluating response to existing and future treatments. Till date different methods have been followed to access and isolate the different biomarkers for AD.

### Potential biomarkers for Alzheimer’s disease from cerebrospinal fluid

**FIGURE 1:**
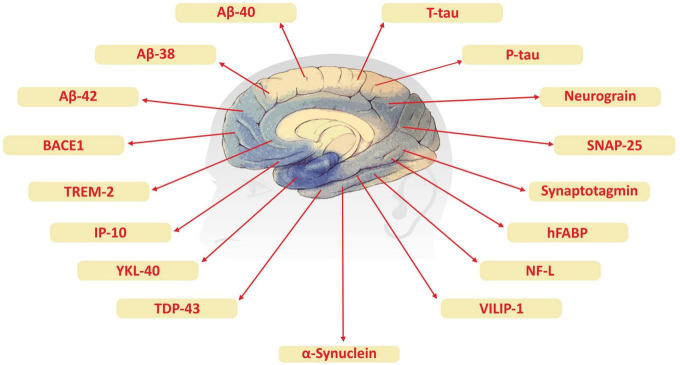
Brain specific biomarker associated with Alzheimer’s disease.

Although advanced neuroimaging techniques have been very useful in assessing structural and physiological changes in the brains of AD patients, clinical biomarkers still represent the most convenient and direct means to monitor the disease state. Despite the fact that PET and cerebrospinal fluid (CSF) biomarkers are useful information for the diagnosis of AD, these methods have limited use due to their sophistication, invasiveness, and high cost. In the search for biological markers of AD, Aβ42, t-Tau, and p-Tau have drawn the special attention of researchers. Various types of brain specific biomarkers associated with AD are shown in Figure 1, CSF specific biomarkers for AD are listed in Table 1 and diagnostic platforms based on four chief biomarkers for AD along with various parameter are listed in Table 2. Aβ42, T-tau and P-tau biomarkers can predict progression from preclinical to clinical AD (Mattsson et al., 2018). The levels and variations of Aβ in CSF have been an essential hallmark feature of AD-type dementia (d’Abramo et al., 2020). It has been suggested that the ratio of CSF Aβ42/Aβ40 can be a superior biomarker because this ratio is quite useful in the differentiation of AD from other non-Alzheimer’s related cognitive changes like the subcortical deficits related to vascular diseases. Aβ42 levels in the CSF are generally lower in comparison to the controls and Aβ42 levels decrease substantially with an increase in disease progression (d’Abramo et al., 2020). The other biomarkers like YKL-40 (Chitinase-3-protein like), VILIP-1 (VLP-1) and, NFL are associated with glial inflammation, neuronal damage and non-specific marker for neurodegeneration respectively can also be used for the diagnosis but the limit of detection (LOD) and accuracy should be validated first in order to gain the specificity (Gaiottino et al., 2013; Olsson et al., 2016).

**TABLE 1:** Biomarkers for Alzheimer’s disease (AD) in cerebrospinal fluid (CSF).

Biomarker	Concerned diagnosis	References
Aβ42	CSF	Olsson et al., 2016; Wellington et al., 2016
Aβ40	Coupled with CSF	Olsson et al., 2016; Wellington et al., 2016; Janelidze et al., 2016b
Aβ38	AD and dementia.	Olsson et al., 2016; Janelidze et al., 2016b
sAPPα	Associated with dementia	Olsson et al., 2016
sAPPβ	Combination with CSF	Olsson et al., 2016
t-Tau and p-Tau	CSF	Olsson et al., 2016
NFL	CSF	Olsson et al., 2016
NSE	CSF	Olsson et al., 2016
VLP-1	CSF	Olsson et al., 2016
HFABP	CSF	Olsson et al., 2016
Albumin-ratio	CSF and dementia	Wellington et al., 2016
YKL-40	CSF	Olsson et al., 2016; Wellington et al., 2016
MCP-1	AD and dementia	Olsson et al., 2016; Wellington et al., 2016
GFAP	CSF and dementia	Olsson et al., 2016; Wellington et al., 2016
Neurogranin	AD	Janelidze et al., 2016b
sTREM2	CSF	Suárez-Calvet et al., 2016
A lpha-synuclein	AD	Majbour et al., 2017

**TABLE 2:** Sensing platforms based on four chief biomarkers associated with Alzheimer’s disease (AD) and their limit of detection (LOD).

Sensing platform	Biomarker	Protein form	Limit of detection	Dynamic range	Sensitivity	Specificity	References
IP-MS	**High performance** plasma amyloid-β biomarkers for Alzheimer’s disease	APP 669-711/Aβ142	2.5Da	∼180 ng/ml	96.7% (AUC)	81.0% (AUC)	Nakamura et al., 2018
IMR	**Assay of plasma** phosphorylated tau protein (threonine 181) and total tau protein in early-stage Alzheimer’s disease	Tau	0.0028 pg/ml	0.001–10,000 pg/ml	0.793 (ROC)	0.836 (ROC)	Yang et al., 2018
Digital-ELISA	**Plasma neurofilament** light as a potential biomarker of neurodegeneration in Alzheimer’s disease	NFL	0.62 pg/ml	unknown	0.84 (ROC)	0.78 (ROC)	Lewczuk et al., 2018
ELISA	**C-terminal neurogranin** is increased in cerebrospinal fluid but unchanged in plasma in Alzheimer’s disease	Neurogranin	3 pg/ml	3–2,000 pg/ml	x	x	De Vos et al., 2015

### Potential biomarkers for Alzheimer’s disease from blood

Blood-based biomarkers are more cost-effective than PET imaging, less invasive than CSF testing, and can be used as viable first-line tools in the multi-stage diagnostic processes (d’Abramo et al., 2020). Since blood testing is a part of clinical routines all over the world which require no special or further training, therefore, blood-based biomarkers for AD are more promising. Although, a lot of progress has been made in understanding the role of biomarkers in the pathophysiology of AD (Tables 1, 2), there is still a need for the development of blood-based biomarkers that can help in the early diagnosis of AD and to understand disease progression. Tau and β-Site APP Cleavage Enzyme 1 (BACE 1) to some levels are useful in this context (Snyder et al., 2014). Tau levels in the blood can be used to predict the onset of future cognitive decline and the levels of BACE1 activity in the blood can also be used to predict the progression of mild cognitive impairment to AD dementia (Hampel et al., 2018). Aβ peptides like Aβ1-42, Aβ1-40, and Aβ1-17 as well as tau are important blood biomarkers to detect AD and its progression. These peptides in plasma can be detected by immunoprecipitation and mass spectroscopy (Pannee et al., 2014; Ovod et al., 2017). It has been found that the plasma levels of Aβ-42, Aβ1-40, and Aβ1-42/Aβ1-40 are low in AD patients but show a significant correlation with CSF levels (Janelidze et al., 2016a). Blood levels of Aβ1-17 also play an important role in the diagnosis of AD. The ratio of free to cell-bound Aβ1-17 levels in the blood helped to understand the difference between healthy individuals and individuals with mild AD with high specificity and sensitivity (Snyder et al., 2014). Tau proteins in plasma have been quantified by sensitive immunoassay techniques and found to be increased in AD patients compared to controls (Zetterberg et al., 2013; Neergaard et al., 2018). Phosphorylated tau (p-tau) proteins are leading blood biomarkers that identify AD, and its underlying pathology. It also highlights future risks of AD. Plasma p-tau (p-tau217 and p-tau181) highlights AD in dementia cases with high accuracy and can also be validated by neuropathological studies. AD progression can be strongly predicted by even baseline increases of p-tau biomarkers However, assay platform comparisons and effects of covariates and accurate biomarker cut-offs for p-tau and Aβ are still lacking necessitating more studies in this direction in the context of Strategic Biomarker Roadmap (Ashton et al., 2021). Moreover, Aβ1–42, Aβ1–40 and phosphorylated tau represent post-translationally modified protein species this explains the involvement of PTMs as biomarkers in AD (Henriksen et al., 2014). Furthermore, recent studies suggested Aβ37/42 ratio could become an improved Aβ biomarker for Alzheimer’s disease of pathogenicity and clinical diagnosis (Liu et al., 2022). Other proteins like axonal protein and neurofilament light (NF-L) are found to be increased in the serum of AD patients and have been found to be comparable with plasma Aβ1–42/Aβ1–40 (Mattsson et al., 2017). However, high NFL-1 concentration in plasma is not associated only with AD but also with other neurodegenerative disorders (Progressive supranuclear palsy and corticobasal syndrome). Recently, investigators questioned if a panel of blood-based biomarkers instead of an individual biomarker could be more useful in the detection of AD. In this direction, O’Bryant used a different set of 30 serum proteins to develop an algorithm that could detect AD with 80% sensitivity and 91% specificity (O’Bryant et al., 2010). It was reported that a panel of three blood markers von Willebrand factor, cortisol, and oxidized LDL antibodies identified using multivariate data analysis, could distinguish between AD patients and normal ones with more than 80% accuracy (Laske et al., 2011). To some level, microRNAs and PTMs can also serve as biomarkers for AD. Alterations in microRNAs levels are reported to be associated with AD pathology and efforts are being made to monitor the changes in the individual miRNAs in blood as biomarkers (Henriksen et al., 2014). A decrease in miR-132-3p levels has been reported to be associated with AD due to the hyper-phosphorylation of tau (Lau et al., 2013). Interestingly, miR-125b and miR-26b have been reported to be increased in AD patients and both are associated with tau phosphorylation (Absalon et al., 2013; Ma et al., 2017). Blood biomarkers are reliable and promising in AD detection Anything that may interfere with the detection of AD biomarkers when their concentration is low in blood is taken care of by modern diagnostic platforms that work with high dilution and high amplification of the specific signal which allows the detection of picomolar/ml or even lesser concentrations of the target biomarker. It was shown that detection by immunoprecipitation followed by liquid chromatography-mass spectroscopy (IP-MS) is a high precision assay for plasma Aβ42-40 ratio, which predicts brain amyloidosis with 90% accuracy (d’Abramo et al., 2020). Recent clinic trials based on biomarker identification for Alzheimer’s disease are listed in Table 3.

**TABLE 3:** List of natural products and synthetic drugs tested for identification of a reliable clinical biomarker in various clinical trials of Alzheimer’s disease.

Therapy	Drug	Stage of AD	Used as (mechanism of action)	References
Anti-amyloid therapy	Solanezumab	Mild	Monoclonal antibody (mAb)	Honig et al., 2018
	Verubecestat	Mild to Moderate stages	BACE inhibitor	Villarreal et al., 2017; Egan et al., 2018
	Verubecestat	Prodromal stage	BACE inhibitor	
	Atabecestat	Preclinical stage		Novak et al., 2020
	Lanabecestat	Early stage		Wessels et al., 2020
	Lanabecestat	Mild stage		
	Aducanumab	Early AD	mAb	Beshir et al., 2022
	CNP520	Preclinical stage	BACE inhibitor	Neumann et al., 2018
	Plasma exchange with albumin 1 Ig	Mild to Moderate stages	Plasma exchange	Boada et al., 2019
	ALZT-OP1a + ALZT-OP1	Preclinical stage	Mast cell stabilizer, anti-inflammatory	Lozupone et al., 2022
	ANAVEX2–73		Anti-tau, Anti-amyloid	
	Crenezumab		mAb	Salloway et al., 2018; Avgerinos et al., 2021
	E2609 (elenbecestat)		BACE inhibitor	Huang et al., 2020; Patel et al., 2022
	Gantenerumab	Prodromal to moderate stage	mAb	Klein et al., 2019
	Gantenerumab and Solanezumab	Early stages		Salloway et al., 2021
	GV-971 (sodium oligomannurarate)	Mild to moderate stage	Aβ aggregation inhibitor	Wang T. et al., 2020
	Solanezumab	Dominantly inherited AD	mAb	Salloway et al., 2021
Non-Anti-amyloid therapy	AC-1204	Mild to Late (severe) stage	Induction of ketosis	Dewsbury et al., 2021
	AGB101 (levetiracetam)	Mild stage	SV2A modulator	Cummings et al., 2020; Tampi et al., 2021
	Aripiprazole	Early stage	Partial agonist at dopamine D2 and 5-HT 1A receptors	Zheng et al., 2020
	AVP-786	Early stage	Sigma-1 receptor agonist, Anti-NMDA receptor	Khoury et al., 2021
	AXS-05	Early stage	Sigma-1 receptor agonist; Anti-NMDA receptor and dopamine-norepinephrine reuptake inhibitor	Huang et al., 2020; Nagata et al., 2022
	Azeliragon	Mild stage	Microglial activation inhibitor, antagonist of the receptor for glycation end products	Burstein et al., 2018; Yang et al., 2021
	OPC-34712 (brexpiprazole)	Mild stage	Agonist of serotonin, 5-hydroxytryptamine1A and dopamine D2 receptors and an antagonist of serotonin 5-hydroxytryptamine2A	Liu et al., 2018; Zarini-Gakiye et al., 2020
	Coconut oil	Early stage	Reduction in ADP-ribosylation factor 1 protein expression	Chatterjee et al., 2020
	COR388	Mild to moderate	Bacterial protease inhibitor	Detke et al., 2020; Seymour and Zhang, 2021
	Escitalopram	Mild stage	Serotonin reuptake inhibitor	Sheline et al., 2020
	Gabapentin Enacarbil	moderate to late stage	Glutamate receptor-independent mechanisms	Liu et al., 2021
	Ginkgo biloba	Early stage	Antioxidant and anti-amyloid aggregation	Vellas et al., 2012; Liu et al., 2021; Xie et al., 2022
	Guanfacine	Healthy but old aged	Alpha-2A-adrenoceptor agonist, a potent 5-HT2B receptor agonist	Barcelos et al., 2018
	Icosapent ethyl (IPE)	Late stage	Omega-3 fatty acids protect neurons from disease	Cummings et al., 2018
	Idalopirdine	Late stage	5-HT6 receptor antagonist	Hung and Fu, 2017
	RVT-101 (intepirdine)	mild to moderate	5-HT6 receptor antagonist	Huang et al., 2020; Akhondzadeh, 2017
	Insulin	Mild stage	Affects metabolism	Craft et al., 2017
	ITI-007 (lumateperone)	mild to moderate	5-HT2A antagonist	Porsteinsson and Antonsdottir, 2017; Cooper and Gupta, 2020
	Losartan, amlodipine, aerobic exercise training, and etc.	mild to moderate	Anti-Angiotensin II receptor, Anti-calcium channel, cholesterol agent	Cummings et al., 2019; Ihara and Saito, 2020
				Szabo-Reed et al., 2019
	Masitinib	mild to moderate	Tyrosine kinase inhibitor	Folch et al., 2015; Ettcheto et al., 2021
	Methylphenidate	——	Dopamine reuptake inhibitor	Khaksarian et al., 2021
	Mirtazapine	——	Alpha-1 antagonist	Correia and Vale, 2021
	MK-4305 (suvorexant)	Mild stage	Orexin antagonist	Zhou F. et al., 2020
	EVP-6124	——	Selective α7 nicotinic acetylcholine receptor partial agonist	Potasiewicz et al., 2020
	Nabilone	Early to mild stage	Anti-cannabinoid receptors 1 and 2	Ruthirakuhan et al., 2020
	Nilvadipine	mild to moderate	Dihydropyridine calcium channel blocker	Lawlor et al., 2018
	AVP-923 (nuedexta)	Moderate to late stage	Uncompetitive NMDA glutamate receptor antagonist, a sigma-1 receptor agonist, and a serotonin and nor epinephrine reuptake inhibitor	Khoury et al., 2021; Liu et al., 2021; Khoury, 2022
	Pioglitazone	Early to Mild stage	Peroxisome proliferator-activated receptor gamma (PPARγ) agonists	Galimberti and Scarpini, 2017
	Troriluzole	Mild to moderate	Glutamate modulator	Yiannopoulou and Papageorgiou, 2020
	TRx0237 (LMTX)	Preclinical or early stage	Tau stabilizers and aggregation inhibitors	Panza et al., 2016
	Vitamin D3	Early stage	Vitamin-D receptor Agonist	Yamini et al., 2018
	Zolpidem zoplicone	Old aged persons	Allosteric modulator of GABA-A receptors	Wu, 2017

### Extracellular vesicles associated with Alzheimer’s disease

Extracellular vesicles (EV) or exosomes (EXOs) were initially considered as cellular trash bags. These lipid-based membrane-bound biological nanoparticles have now emerged as a new paradigm of cell-to-cell communication which has implications for both normal and pathological physiology (Raposo and Stahl, 2019). Recently, extracellular vesicles have been widely used as biomarkers and are found to play an important role in the pathogenesis of Alzheimer’s disease. The presence of disease-related proteins in these extracellular vesicles from AD patients have been actively studied to use them as biomarkers to predict the development of AD before the appearance of clinical symptoms (Lin et al., 2019). They are excreted in the urine and saliva of both healthy and diseased individuals which makes them an excellent choice for diagnostics in diseases like AD as compared to other invasive methods which require lumbar puncture or brain autopsy for diagnosis (Watson et al., 2019). EVs are released from various cell types including the cells associated with neuron-glia communication, neuronal progression, and regeneration from CNS (Surgucheva et al., 2012; Bronisz et al., 2014; Janas et al., 2016). It is widely accepted that EVs are good biomarkers but their nature, regulation, sorting and molecular composition need to be further investigated. Interestingly, the role of exosomes in stimulating aggregation of amyloid-beta (Aβ) peptides *in vitro* and *in vivo* was demonstrated and as was their role in the uptake of Aβ by cultured astrocytes and microglia under *in vitro* conditions (Dinkins et al., 2014). By preventing the secretion of EVs *via* inhibition of neutral sphingomyelinase 2 (nSMase2), a key regulatory enzyme generating ceramide from sphingomyelin, with GW4869, they observed a significant reduction of Aβ plaques in the mice brain. Moreover, exosomal markers such as Alix and flotillins have been found to be associated with the amyloid plaques of the brain of post-mortem tissues of human AD patients when compared to post-mortem samples of healthy individuals, thereby reflecting specific association of EVs with amyloid plaques (Hu et al., 2016). Further, an increase in levels of tau in exosomes have been reported in CSF at an early phase of AD (Saman et al., 2012; Asai et al., 2015). Levels of several exosomal miRNAs that are altered in AD points to their roles in regulating certain events related to AD neurodegeneration as the ability of miRNAs to target genes related to tau phosphorylation, APP processing, and apoptosis is already known (Liu et al., 2014; Ma et al., 2017). The miRNA-193b and miRNA-125b-5p are the two most common exosomal miRNAs identified and are reported to be expressed differentially in exosomes isolated from serum, plasma and CSF of AD patients (Soares Martins et al., 2021). Therefore, miRNA-193b and miRNA-125b-5p are thought to be potential putative peripheral biomarkers of AD pathogenesis. EVs as potential biomarkers for AD can open up a wide arena of discoveries associated with it; however, a proper evaluation and correlation is needed for it as well.

### Circadian genes associated with Alzheimer’s disease

It has been reported that that the levels of the main risk factor for AD progression i.e., Aβ in the brain are regulated by circadian rhythms and the sleep-wake cycle (Kang et al., 2009). Circadian rhythms (CR) are biological clocks that coordinate internal time with the external environment to regulate various physiological processes like the sleep-wake cycle, mental, behavioral and physical changes. Circadian rhythms are managed by a molecular clock in the suprachiasmatic nucleus (SCN) that resides in the anterior part of the hypothalamus. Molecular clockwork consists of different core clock genes such as period circadian regulators 1 and 2 (PER1 and PER2), circadian locomotor output cycles kaput protein (CLOCK), brain and muscle ARNT-like 1 (BMAL1), cryptochrome circadian regulators 1 and 2 (CRY1 and CRY2), These genes are organized in a transcription-translation feedback loop that oscillates every 24 h. Any mutation or alteration in these genes or their subsequent proteins has functional impacts on the CR (Shkodina et al., 2021). Disruptions in CR cause cognitive impairment, psychiatric illness, metabolic syndromes, and are thus considered as a significant risk factor in the onset of cerebrovascular and neurodegenerative disorders such as Alzheimer’s, Parkinson’s, and Huntington’s diseases. Moreover, disrupted sleep wake-cycle and alterations in circadian rhythms are seen to lead to AD progression by dysfunctional tau metabolism. This is achieved by changing the conformation and solubility of tau in the brain involving the post-translational modifications by decreasing the activity of major regulators of tau phosphorylation, especially cyclin-dependent kinase 5 (cdk-5) (Di Meco et al., 2014). During the early development of AD, there is a disruption in the normal expression of clock genes (especially *Cry1*, *Cry2*, and *Per1*) not only in the central pacemaker but also in other brain areas like the cortex, hippocampus, and cerebellum supporting circadian regulation (Bellanti et al., 2017). Since sleep and circadian rhythm dysfunction (SCRD) are very common in the early stage of AD, it is considered as a potential early biomarker for detecting AD. A proper understanding of the circadian clock and investigations of core clock components including *Bmal1*, *Clock*, *Per1*, *Per2*, *Cry1*, *Cry2*, *Rev-erb*α, and *Ror*α by genomic, proteomic, and metabolomic studies and its influence on several key processes involved in neurodegeneration might be helpful to be manipulated at early stages to promote healthy brain aging and decrease the chances of AD progression on one hand and uncover their potential use as biomarkers and hallmarks of circadian disruption during early stages of Alzheimer’s disease progression.

## Post-translational modifications in Alzheimer’s disease

**FIGURE 2:**
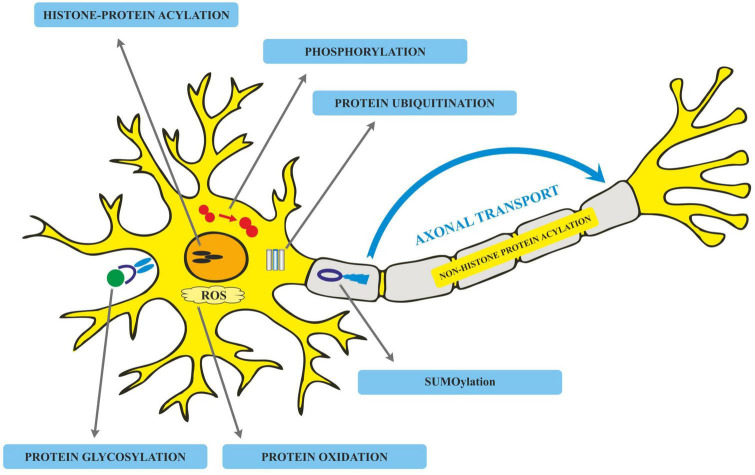
Various post-translational modifications (PTMs) associated with Alzheimer’s disease.

**FIGURE 3:**
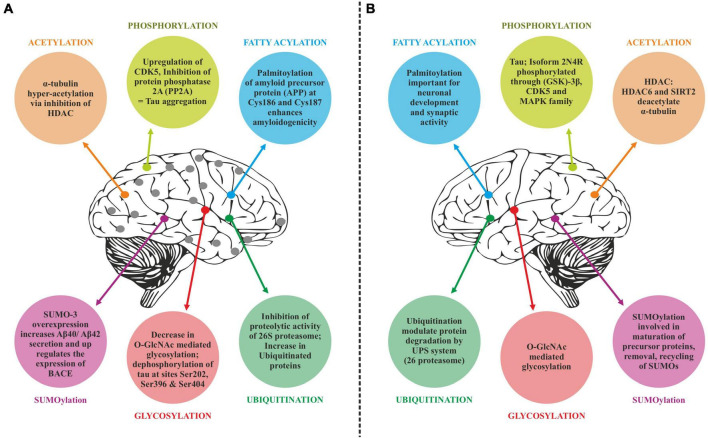
Post-translational modifications (PTM) specific modifications associated with Alzheimer’s disease vs. normal brain. **(A)** PTMs associated with Alzheimer’s disease. **(B)** Normal PTMs.

Translated messages in the form of protein undergo important modifications usually after their synthesis. These post translational modifications (PTMs) usually occur in the various cellular compartments like the Endoplasmic reticulum, Golgi complex, Nucleus, as well as Cytoplasm (Blom et al., 2004). PTM’s play an important role in regulating the structure, localization and, activity of proteins (Deribe et al., 2010). PTMs impact the hydrophobicity of proteins and induce changes in their structural conformations and thus define protein function as well as their interactions with other proteins (Ravid and Hochstrasser, 2008; Ardito et al., 2017). PTMs involve the addition of varied chemical moieties to a target protein that include phosphate, acyl, methyl, and glycosyl groups catalyzed by specific enzymes. PTMs not only define the structural integrity of proteins, these modifications usually cause the proteins to lose or enhance their normal function as well (Santos and Lindner, 2017). Unusual PTMs of various proteins like APP, secretases, tau, various kinases, and phosphatases are linked to the development of neurodegenerative diseases. The appearance of NFTs, senile plaques and aggregation of toxic amyloid β peptides are some of the hallmarks of abnormal PTMs in Alzheimer’s disease. These posttranslational protein modifications are associated with memory weakening, cognitive impairments, reduced synaptic plasticity and thus rapid progression to AD (Chong et al., 2018). Exploring post-translational modifications and understanding their molecular mechanism will open a window to develop effective and rational therapeutic interventions to counter these neurodegenerative disorders. Various PTMs associated with AD are shown in Figure 2. PTM specific modifications associated with AD vs. normal brain are also shown in Figure 3 and discussed in detail here.

### Phosphorylation

Phosphorylation is one of the most frequently occurring PTMs in proteome biology, and about 30% of proteins undergo such modifications (Sacco et al., 2012). It involves the addition of a phosphate group to amino acids particularly to serine, threonine and tyrosine residues (Humphrey et al., 2015). This modification impacts the structure and function of proteins and determines their fate in terms of signaling, trafficking as well as metabolism; thus playing an important part in regulating the normal physiological processes (Ravid and Hochstrasser, 2008). Protein phosphorylation is one of the most predominant PTMs that drives enormous cellular cascades in living cells. The phosphorylation of proteins is reversible and very specific. These events of phosphorylation and dephosphorylation are catalyzed by diverse kinases. A variety of kinases like Akt, Erk, and PKA. GSK3β and Cdks have been found to be overexpressed with enhanced activity accompanied by a significant drop in phosphatases activity in AD patients (Alquezar et al., 2021). Accumulating evidence report that various proteins on altered phosphorylation in these neuro disorders drive the cell death signaling cascades (Suprun, 2019).

#### Tau phosphorylation

**FIGURE 4:**
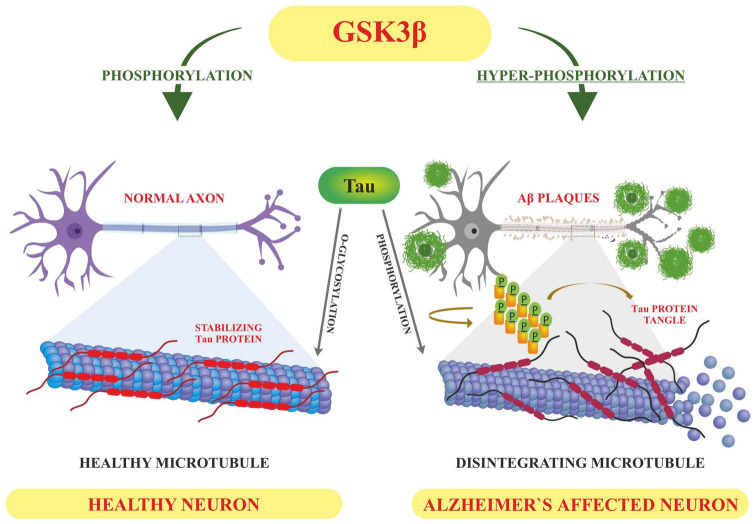
Displaying how the altered post-translational modifications (PTM) leads to Alzheimer’s pathology-based on the literature surveyed GSK3β dependent hyperphosphorylation of tau proteins results in accumulation of NFT’S and deposition of Aβ plaques–hallmarks of Alzheimer’s disease whereas O-linked glycosylation of tau proteins hampers its aggregation thus promoting normal polymerization processes.

Tau is involved in the stabilization of microtubules, as mentioned earlier. Six different isoforms of tau are usually expressed in normal mature human brain which are found to be hyperphosphorylation in AD brains. These six isoforms of tau protein with varied amino acid chain length (331 to 441) emanate from alternative splicing of a single gene. Tau protein has different domains like N terminal projection domain ranging from 1 to 165 amino acids, proline rich domain from 166 to 242, microtubule binding region (MTBR) starting from 243 to 367 as well as carboxy terminal domain 368–441. Tau proteins bear 85 phosphorylation sites; with nearly half of them found to undergo phosphorylation and the majority of these phosphorylated stretches are found in proline rich domain of tau protein, flanking microtubule binding region (Goedert et al., 1989; Hanger et al., 2009). These phosphorylation events and isoform expression are developmentally regulated and it determines the complexity of embryonic cytoskeleton plasticity (Hanger et al., 2007). However, any unusual alterations in its structural conformation or in phosphorylation events impact its binding affinity with microtubule. Tau phosphorylation is residue specific and is mediated by different types of kinases. These kinases are proline rich domain specific kinases like GSK-3, Cdk5, and AMPK, non-proline directed phosphorylating kinases as well as Fyn kinases (Iqbal et al., 2016). Phosphorylation of tau protein by GSK3β at residues other than that of microtubule binding region like, thr231 pro232, and s214 has been found to impede its association with the microtubule thus affecting its anterograde transport (Figure 4). This residue specific phosphorylation leads to the induction of some conformational alterations which in turn lead to hyperphosphorylation of tau protein. It has been found that phosphorylation of Ser 404 accommodated by C-terminal domain alters the tau conformation (Luna-Munoz et al., 2007). Although GSK3B has been found to phosphorylate 36 residues of tau protein, however, preferable sites spotted by 2D phosphor-peptide mapping include Ser199, Thr231 as well as Ser413, respectively. Besides aforementioned mapping, monoclonal antibody technology has been applied to spot the phosphorylation sites that play a role in AD pathology, and it has been found that phosphorylation at specific motifs like Thr212/Ser214 and Thr231/Ser235 are exclusively seen in PHF (Ebneth et al., 1998; Despres et al., 2017). Interestingly phosphorylation of tau proteins at ser293 and ser305 neutralizes its ability to form aggregates without affecting tubulin polymerization. GSK3β like other kinases recognizes primed target proteins as it has been noted that Thr231 residue of tau proteins needs to be primed by other kinases like Cdk5 which is followed by GSK3b mediated phosphorylation. This phosphorylation of tau protein, in turn, impacts its binding ability with the microtubules thus hampering their polymerization and other neuronal functions (Stoothoff and Johnson, 2005). Cdk5 another proline region-specific kinase involved in hyperphosphorylation of tau hampers the ability of tau protein to bind and stabilize microtubules leading to the disruption of axonal transport and neuronal death-an important contributor to the pathology of neurodegenerative diseases like AD (Piedrahita et al., 2010). Growing evidences report that dephosphorylated tau protects nuclear DNA from heat damage and other kinds of oxidative stress insults however tau in hyperphosphorylated form is believed to halt protective functionalities associated with non-phosphorylated forms in neuronal entities (Sultan et al., 2011). Cdk5 isoform is expressed in the brain, unlike cell cycle-related Cdks serine/threonine kinase, shows affinity toward the proline rich domain and phosphorylates some preferable residues like Ser202, Thr205, Ser235, and Ser404; which are found to be essential in regulating Tau-Mt binding. Cdk5 is activated by neuron specific p35 and p39 proteins-which are not considered to be cyclins. Thus, Cdk5 mostly acts in neural cells and is activated by binding to neuronally enriched activators, p35 and p39, to display its functions predominantly in post-mitotic neurons. While the defect of Cdk5 is disparaging to the CNS, its hyperactivation is also lethal to neurons. Some studies have also unraveled the various roles of cdk5 in neuronal remodeling as well as modulation of synaptic transmission (Fleming and Johnson, 1995; Cruz et al., 2003). Fyn, a tyrosine kinase, phosphorylates tyr18 residue and has been reported to precede the deposition of NFT as well as PHF associated with AD pathology (Lee et al., 2004). Exhaustive research has been carried out and it has been observed that hyperphosphorylation of tau protein makes it susceptible to aggregation and leads to the formation of tau assembly *in vitro* conditions whereas dephosphorylation events reverse its assembly to normal levels as well as stabilizes microtubules back. Coordinated interactions of kinase as well as phosphatases have been seen to maintain the phosphorylation status of tau protein and imbalance in such interplay usually drives its aggregations associated with several tauopathies. Various *in vitro* studies have been carried out which unraveled the involvement of specific phosphatases involved in the regulation of phosphorylation in AD. It has been found that PP1, PP2A as well as PP5 are residue specific and dephosphorylates the tau protein at particular residues like ser199, ser20, thr212, and ser409 respectively, thus maintaining the balance between kinase and phosphatase action (Liu et al., 2005). These phosphates also drive the positive feedback cycles by regulating the ERK/MAPK signaling cascade that otherwise activates the GSK3β which progresses the aggregation of tau proteins. Among these phosphates PP2A is most extensively studied as it regulates about 70% of tau dephosphorylation in the human brain. Besides phosphorylation, tau proteins undergo other post translational modifications like acylation and glycosylation playing a role in standard microtubule dynamics (Qian et al., 2010). Emerging evidences suggest that overexpression of GSK3β as well as other kinases like Cdk5 plays a part in the progression of these neurodegenerative disorders like AD thus gaining a special interest in treating these disorders by employing therapeutic intervention in the form of specific inhibitors which can inactivate these kinases. Lithium-One of the most commonly used GSK3β inhibitor that inactivates the gsk3b by enhancing its ser-9 inhibitory phosphorylation which acts as a pseudo substrate and prevents its activity (Zhang et al., 2003). It has been reported that the application of lithium treatment in some experimental murine models that are overexpressing human APP, showed some encouraging results in retarding the neuropathology and cognitive impairments (Hu et al., 2009; Undurraga et al., 2019). Lithium is the only GSK-3β inhibitor that has been in clinical use for a significant time. However, we know that lithium lacks target specificity, and shows adverse side effects and high toxicity. Although two molecules AZD-1080 (AstraZeneca) and NP-12/Tideglusib (Noscria) reached the clinic in 2006, AZD-1080 was later on abandoned due to its nephrotoxicity as observed in phase I clinical trial while as NP-12 is currently in phase IIb trials for Alzheimer’s disease and paralysis supranuclear palsy. Meanwhile presently, an increasing number of GSK-3β inhibitors are being tested in preclinical models, and it is anticipated that some of the potent inhibitors will enter clinical trials (Eldar-Finkelman and Martinez, 2011). Recent developments in the field of small-molecule inhibitors of CDKs have led to several compounds with anticancer potencies both *in vitro* and *in vivo* and models of cancer. However, the specificity of the inhibitors, which inhibit close isoforms, is not fully achieved yet, especially for CDK5, which is involved in neurodegenerative diseases (Łukasik et al., 2021). Other specific inhibitors have been developed with improved therapeutic effects, some of which are non-ATP competitive inhibitors that are selective and significantly less toxic than others like L803-mts as well as TDZD-8, VP0.7 (Kaidanovich-Beilin et al., 2004; Kaidanovich-Beilin and Eldar-Finkelman, 2006). Other conventional ATP competitive inhibitors include paullones, indirubin, SB415286 and SB216763 as well as AR-A014418 respectively (Leost et al., 2000; Bhat et al., 2007). Tau phosphorylation as well as amyloid beta deposition is impeded by treating the transgenic mouse models that are overexpressing APP, as well as reducing the memory weakening in the Morris water maze (Serenó et al., 2009; Ly et al., 2012). A recent finding suggests that selective GSK3 inhibitor SAR502250 is effective in contributing toward neuroprotection as well as diminish behavioral impairments in rodent models with neuropsychiatric abnormalities. Treatment of P301L human transgenic mice with this inhibitor was seen to reduce the tau phosphorylation thus impairs the formation of tau aggregates. Besides implications of specific inhibitors, genetic knockdowns of GSK isoforms have been also shown to recover cognitive abnormalities associated with various murine models. So these findings suggest that intervention of these kinase inhibitors can possibly act as disease modifying tactics to encounter AD related conditions (Griebel et al., 2019).

### Acetylation

Alzheimer’s disease (AD), also known as a protein misfolding disease, is a degenerative and incurable terminal disease of the central Nervous system (CNS) characterized by the presence of two main types of protein aggregates that include amyloid plaques and NFTs. Both genetic and non-genetic (environmental) factors are involved in the development of AD (Singh and Li, 2012; Tönnies and Trushina, 2017). However, the exact gene-environment interactions involved in the development of AD have not been delineated yet. Epigenetic changes are involved in integrating genetic and environmental interactions. These epigenetic processes are thus considered heritable changes in gene expression without causing any change in the coding sequence of genes. Most common epigenetic alterations reported that impact phenotypic outcomes include histone modifications like acetylation, phosphorylation, methylation, ubiquitination, ADP ribosylation, and SUMOylation; DNA methylation and non-coding RNA (Miller and Grant, 2013). These epigenetic changes regulate gene expression by altering the chromatin structure thereby increasing access to transcription factors (Venkatesh and Workman, 2015). The histone modifications involve acetylation and deacetylation of histone proteins which are the chief protein components of chromatin in the form of histone octamers. Each histone octamer consists of four core histones which include H2A, H2B, H3, and H4 (Shahbazian and Grunstein, 2007). These positively charged histone octamers wrap around negatively charged DNA to form nucleosomes-the basic components of chromatin structure. Acetylation and deacetylation of these histones define the chromatin configuration. Acetylation at the N-terminal tails of H3 and H4 histones neutralizes their positive charge thereby reducing the binding of histone to DNA. Acetylation of core histones, considered as the markers of an open configuration of chromatin, opens up the chromatin to facilitate gene transcription (Struhl, 1998). On the other hand, deacetylation does the opposite. Thus, we can say that histone acetylation promotes transcriptional activation, while as histone deacetylation leads to transcriptional repression of genes. These histone modifications regulate critical cellular processes that include cell proliferation, differentiation, apoptosis, inflammation, neuronal plasticity, and metabolic reprogramming (Thiel et al., 2004). During the process of histone acetylation, Histone Acetyltransferases (HATs) transfer an acetyl group from acetyl coenzyme A to the lysine residues present at the N-terminal domains of core histones. This acetylation neutralizes the positive charge in histones and thus reduces affinity between histones and negatively charged phosphates in DNA. Therefore, it opens up chromatin to promote the transcriptional activity of genes (Bannister and Kouzarides, 2011). In contrast to this, histone deacetylase (HDAC) removes the acetyl groups from histones which results in silencing of gene expression. Thus, both histone acetyl transferases (HATs) and histone deacetylases (HDACs) play an important role in chromatin remodeling (Kuo and Allis, 1998). HATs are broadly divided into two classes based on their subcellular localization: A-type HATs, and the B-type HATs which are seen in the nucleus and cytoplasm respectively (Mersfelder, 2008). A-type HATs are further divided into three subclasses based on their structural homology: (1) GNAT family represented by Gnc5, PCAF, and ELP3; (2) MYST family containing Tip60, MOZ/MYST3, MORF/MYST4, HBO1/MYST2, and HMOF/MYST1; and (3) p300/CBP family containing p300 and CBP. Multiple studies have been conducted to investigate the role of these HATS in the etiology of AD (Lu et al., 2015; Li et al., 2021). HAT activity of Tip60 was shown to regulate the expression of genes that are related to behavior, learning, memory, and neuronal apoptosis in Drosophila (Xu et al., 2014). Similarly, HAT activity of CBP and p300 is related to long-term memory and neuronal survival. An interesting study was conducted in behavior-trained rats where hyperacetylated H2B/H4 in the promoters of the synaptic-plasticity-related genes was observed upon expression of CBP/p300 and PCAF (Chatterjee et al., 2013). Furthermore, AD pathological contexts also show a critical CBP/p300 loss with histone H3 deacetylation (Rouaux et al., 2003). A genome-wide study was conducted to examine the histone H3 acetylation pattern in the entorhinal cortex of AD patient samples and compared with the control subjects using chromatin immunoprecipitation and highly parallel sequencing (Marzi et al., 2018; MacBean et al., 2020). Genes involved in the progression of AD like amyloid-β and tau showed highly enriched acetylated peaks. Ramamurthy E. et al. carried out cell type-specific histone acetylation pattern analysis of AD patients and controls and observed differential acetylation peaks in the early onset risk genes (*APP, PSEN1, PSEN2, and BACE1*), and late onset genes *(BIN1, PICALM, CLU, ADAM10, ADAMTS4, SORL1*, and *FERMT2*) associated with the pathogenesis of AD (Marzi et al., 2018; Ramamurthy et al., 2020).

The histone deacetylases are also classified into four groups based on their homology to yeast enzymes: Class I HDACs consist of HDACs 1, 2, 3, and 8; Class II HDACs which are further divided into two subclasses, class IIa including HDACs 4, 5, 7, 9, and class IIb HDACs 6, 10; Class III HDACs which include SirT1-7; and class IV HDAC that has a single member-HDAC11 (Seto and Yoshida, 2014). Although class I HDACs are ubiquitously expressed, in comparison to HDAC1, HDAC2 and –3 show the highest expression levels in brain regions associated with memory and learning, such as the amygdala, hippocampus, and cortical areas (Volmar and Wahlestedt, 2015). The subcellular localization and expression levels of individual HDAC isoforms differ in different cell types during various stages of AD progression. HDAC2 is reported to negatively regulate memory and synaptic plasticity (Guan et al., 2009). The mice overexpressing HDAC2 showed hypoacetylation of histone H4 on their K12 and K5 residues. These mice also showed memory impairment and decreased number of synapses (Kumar et al., 2005). Since HDAC2 shows high levels of expression in the post-mortem brain samples of AD patients. HDAC2 downregulation by using short-hairpin-RNA restored the memory impairment and synaptic plasticity in CK-p25 mice indicating the critical nature of HDAC in memory formation and synaptic plasticity (Gräff et al., 2012). In another study, HDAC3 was shown to be critical for regulating synaptic plasticity in a single neuron or neuronal populations. Some established human neural cell culture models with familial AD (FAD) mutations showed a significant increase in HDAC4 levels in response to Aβ deposition in this cell model (Citron, 2010). Targeting HDAC4 by the selective inhibitor TasQ rescued the expression of genes involved in regulating neuronal memory/synaptic plasticity (Mielcarek et al., 2015). While high levels of HDAC6 protein were seen in the cortices and hippocampi of AD post mortem brain samples, reducing endogenous levels of HDAC6 were reported to restore learning and memory ability in the mouse model of AD. This happens partly because HDAC6 is shown to significantly decrease tau aggregation and promote tau clearance *via* acetylation (Fan et al., 2018). Thus, several studies have demonstrated that abnormal acetylation of core histones is involved in the etiology of AD. In addition to histones, altered acetylation of non-histone proteins which include NF-κb, p53, alpha tubulin and tau have also been reported in the pathogenesis of AD. Tau acetylation is mediated by p300 and CBP histone acetyl transferases at different residues. Acetylation of tau reduces its solubility and thus affects its intrinsic propensity to aggregate. Intracellular tau acetylated at Lys280, preferably by CBP, is seen during all stages of AD disease. Tau acetylation is known to suppress the degradation of phosphorylated tau (Chen et al., 2001; Park et al., 2013). Moreover, both acetylated and hyperphosphorylated-tau were reported to show similar spatial distribution pattern. Conversely, HDAC6 activity promotes deacetylation tau, which then contributes to enhanced tau-microtubule interactions and microtubule stability (Carlomagno et al., 2017).

Histone deacetylases (HDAC) inhibitors for the treatment of AD: A growing body of evidence considers HDAC proteins as therapeutic targets for the treatment of AD. HDAC inhibitors may be alternative drugs to potentially protect against the impairment of cognition in AD patients. It is suggested that HDAC inhibitors may be a good alternative to conventional drugs to potentially improve the cognition features in AD patients (Xu et al., 2011). It has been shown that inhibitors targeting HDACs are involved in improving memory and cognition in the mouse model of AD. In AD animal models, HDAC inhibitors show neuroprotective activities, and thus provide a promising strategy for the treatment of AD (Sun et al., 2017). However, care should be taken when using pan-HDAC inhibitors (non-selective HDAC inhibitors) to treat AD because these HDAC inhibitors are poorly selective and often cause some undesired side effects (Cheng et al., 2015; Sun et al., 2017). Thus, evaluating the role of individual HDAC isoforms fin memory, learning and in the pathogenesis of AD becomes all the more important for the discovery and development of more selective HDAC inhibitors. Isoform selective HDAC inhibitors may, however, greatly eliminate side effects and toxicities associated with pan inhibitors and offer improved efficacy (Balasubramanian et al., 2009).

### Glycosylation

Glycosylation involves the attachment of specialized forms of sugars called glycans to the target proteins through glycosidic bonding like N and O linkages (Spiro, 2002). Glycans are the sugar moieties linked to protein, lipids or other molecular entities and their structural complexity varies depending on the type of monocarbide core it bears. These glycans regulate the various functionalities associated with the normal functioning of cells like cellular signaling (Varki, 2017). These tissue specific glycosylation patterns have also been reported. Unusual alterations in such modification have implications for different diseases including several neurodegenerative disorders (Abou-Abbass et al., 2016). Nearly 50% of proteins have been observed to undergo such modifications. Glycosylation of proteins usually occurs in the ER and Golgi complex to facilitate the trafficking of proteins to different locations like mitochondria, cytoplasm, plasma membrane, nucleus etc., (Kizuka et al., 2017). This modification is usually catalyzed by enzymes to add glycans to target proteins through glycosidic bonding. Based on the type of glycosidic bonding between sugar moiety and target protein, it is categorized into N-linked and O-linked modifications (Shental-Bechor and Levy, 2008; Haukedal and Freude, 2021). N-linked glycosylation involves the attachment of N-acetyl glucosamine to the asparagine residues of target proteins through β-1N linkages. This modification starts with the synthesis of glycan (14 carbon moiety) from N acetyl glucosamine and mannose sugar, which undergoes further sugar modification, followed by the transfer of modified sugar chain to a particular protein thus acquiring complex form. This whole process is carried out by a specific set of enzymes like mannosidases and glucosidases of ER as well as glycosyltransferases of the Golgi complex (Moremen et al., 2012; Reily et al., 2019). On the other hand, O-linked glycosylation occurs in the several cellular compartments like cytoplasm, nucleus and mitochondria and involves the linking of O-glycans usually in the form of N-acetyl galactosamine or N-acetyl glucosamine to target proteins through Ser/Thr residues without undergoing any kind of alteration in the form of trimming of precursor sugar as is seen in N linked type. Many other sugar modifications are also reported which are carried out by enzymes like glycosyl transferases. These modifications involve the addition of galactose, N acetyl glucosamine, sialic acid etc., to target proteins (Clausen and Bennett, 1996; Akasaka-Manya and Manya, 2020). These sugar modifications usually determine the stereochemistry of target proteins which in turn define the structural organization as well as protein stability (Shental-Bechor and Levy, 2008). In the progression of Alzheimer’s disease (AD), glycosylation is associated with tauopathies. Several proteins like APP and BACE1 are seen to be glycosylated in tauopathy patients (Kizuka et al., 2015). It has been seen that O-glycosylation of tau hampers its aggregation without causing any kind of obstruction in its normal polymerization activity (Mietelska-Porowska et al., 2014). Thus, it has been proposed that glycosylation of tau plays a protective role by blocking tau aggregation. Few O-linked glycosylation sites have been spotted in tau protein at residues which are targets of tau phosphorylation as well. These residues include S400, S238, and S409 (Yuzwa et al., 2011). Unlike O-glycosylation, N-type modifications of tau protein have been observed to affect the subcellular localization of tau protein in AD patients (Alquezar et al., 2021). Transgenic mice models of AD have shown a significant disparity in O-GlcNAcylation and phosphorylation with tau protein being hyperphosphorylated at specific serine residues followed by a drop in O-GlcNAcylation levels (Bourré et al., 2018). Both O-linked glycosylation and phosphorylation of tau protein are well-adjusted in normal conditions and it has been observed that hyperphosphorylated protein shows a decreased glycosylation thus impacting its nuclear localization (Lefebvre et al., 2003). Thus we can say that O-glycosylation competes with phosphorylation and shields tau protein from hyperphosphorylation at particular stretches of serine residues by Protein Kinase A (PKA), therefore playing a protective role in normal brain functions. However, it has been found that N-glycosylation helps tau phosphorylation by hampering dephosphorylation or by activating PKA mediated tau phosphorylation, and thus promotes the progression of pathology associated with Alzheimer’s disease (Liu et al., 2004). Altered glycosylation patterns of many other proteins involved in the progression of neuro disorders like APP have been reported as well. Glycosylated APP has been reported in CSF of patients showing AD pathology (Schedin-Weiss et al., 2014). It has been reported that N-bonded glycan display modulation of amyloid beta production and structural variations in such glycans alters APP transport as well as trafficking. O-linked glycosylation of APP directs it to plasma membranes to promote the processing of APP through non-toxic, non-amyloidogenic pathways, thus lowering the production of toxic amyloid beta peptides (Chun et al., 2015a). N-glycans are also involved in the trafficking and secretion of APP. It has been shown that blocking the activity of mannosidase hampers the production of hybrid and complex forms of N glycan which affects APP transport and other proteins to the synaptic membrane (Bieberich, 2014). Recently site-directed mutagenesis approach has shown that specific O-glycosylation at Thr576, directs its transport toward the plasma membrane and the subsequent endocytosis of APP elevates Aβ levels (Chun et al., 2015b). Treatment of experimental 5XFAD mice with a small molecule inhibitor blocking the glycosylation of gamma secretase lead to reduced production of AB peptides, thus slowing the progression of neuroinflammation followed by recovery in memory impairments (Kim et al., 2013). Patients displaying AD pathology have been found to contain BACE1 post translationally altered with bisecting GlcNAc however blocking this modification using knockout approach slows down APP processing followed by reduced amyloid deposition. Experimental knockout Mgat3-gene mice have been shown to alleviate cognitive abnormalities as well as deposition of amyloid beta aggregates (Elder et al., 2010; Haukedal and Freude, 2021). Besides these enzyme dependent modifications, there are enzyme independent post translational modifications called glypiations which involve the covalent attachment of sugars to lysine residue of target proteins. This alteration usually produces advanced glycation end products (AGES) which are speculated as glycotoxins playing a role in age related diseases (Cho et al., 2007). It has been observed that glycation modifications cause aggregation of the tau proteins by hampering the ubiquitination process required for tau degradation. Moreover. this modification usually reduces the binding affinity of tau protein toward microtubule thus affecting polymerization followed by defective axonal transport and other synaptic functions (Ko et al., 1999).

### Fatty acylation

The attachment of long chain fatty acids like palmitate (16 carbon saturated fatty acid) and myristate (14 carbon saturated fatty acid) by amide and thioester linkages respectively is called fatty acylation (Lanyon-Hogg et al., 2017). The mechanism of fatty acylation tunes various cellular processes such as protein-protein interactions, membrane targeting, and intercellular as well as intracellular signaling. Dysregulation in the process of fatty acylation leads to the development of disease conditions including neuronal defects (Wright et al., 2010; Tate et al., 2015). Of all the three lipid modifications viz myristylation, prenylation and palmitoylation; myristoylation and palmitoylation are the most common acylation processes and only palmitoylation is reversible (Resh, 2016).

#### Palmitoylation

S-Palmitoylation is a vital post-translational modification that is important for the function and trafficking of various synaptic proteins. Palmitoylation reactions are carried out by palmitoyl acyltransferases (PATs) that catalyze the attachment of palmitate (16 carbon) covalently to cysteine residues through thioester bonds (Cho and Park, 2016). Palmitoylation (addition of sulfhydryl group to palmitoyl group) is important for synaptic activity and an increase in palmitoylation contributes to pathogenesis of AD to a large extent (Bhattacharyya et al., 2013). BACE 1 or β-secretase, a 501 amino acid type 1 transmembrane aspartic acid protease, associated with the retroviral aspartic β-secretase protease and pepsin family, leads to APP cleavage in the amyloidogenic pathway generating Aβ including pathogenic Aβ42 (Vassar et al., 2009). S-Palmitoylation of BACE 1 takes place at specific Cys residues viz Cys-474, 478, and 485, out of which Cys 474 is a part of the transmembrane domain. Mutations that change these cysteine residues to alanine cause displacement of BACE1 from the lipid rafts without affecting the processing of APP and produce peptides of amyloid (Vetrivel et al., 2009). S-Palmitoylation of BACE1 and its role in Alzheimer’s disease was studied by developing a gene knock-in transgenic AD mice, where the cysteine residues of S-palmitoylation were changed to alanine residues. The lack of S-Palmitoylation of BACE 1 was observed to reduce the cerebral amyloid burden in AD mice significantly, it also reduced cognitive defects. This suggests that the intrinsic S-palmitoylation of BACE 1 has an impact on the pathogenesis of amyloid and further cognitive decline (Andrew et al., 2017). Palmitoylation targets APP to the lipid rafts and enhances its BACE-1 mediated cleavage which ultimately increases the amyloidogenic processing. Palmitoylation inhibitors impair the processing of APP and α and β (a family of proteolytic enzymes that cleave APP to produce amyloid beta peptides). Acyl Coenzyme A Cholesterol acyltransferase (ACAT) inhibitor, known to redistribute cellular cholesterol, inhibits APP palmitoylation and significantly reduces Aβ generation (Bhattacharyya et al., 2013).

#### Myristoylation

Myristoylation, is a eukaryotic post and co-translational modification, is the covalent attachment of myristic acid, a 14-carbon saturated fatty acid, to the N-terminal glycine of proteins. Proteins that are destined to be myristoylated begin with the sequence Met-Gly. N-M-T acts on myristoyl coenzyme A and transfers myristate from it to N-terminal glycine to a varied range of substrate proteins. These myristoylated proteins play critical roles in many signaling pathways to mediate protein-protein and protein-membrane interactions, subcellular targeting of proteins etc. Myristoylation is mediated by the enzymes commonly known as N-myristoyltransferases (N-M-T). In vertebrates myristoylation is carried out by NMT1 and NMT2-members of the GCN5 acetyltransferase superfamily, expressed in nearly all tissues and are reported to be involved in the progression and development of various pathological conditions which include Alzheimer’s disease, cancer, epilepsy etc., (Thinon et al., 2014). A key event in AD is the cleavage of APP by β-secretase to generate APP C99, which then undergoes additional cleavages by γ-secretase to produce Aβ40 and Aβ42 peptides (Su et al., 2010). The presenilin-1 (PSEN1) and presenilin-2 (PSEN2) genes encodes the major component of γ secretase responsible for APP cleavage resulting in the subsequent formation of Aβ peptides (Delabio et al., 2014) and altered APP processing is usually seen in AD patients carrying PSEN mutation. It has been shown that calmyrin, a calcium binding myristoylated protein, plays a versatile role in intracellular signaling and is also important in the functioning of presenilin (Stabler et al., 1999). Calmyrin preferentially interacts and colocalizes with PSEN2. The co-expression of calmyrin and PSEN2 in Hela cells were reported to modify the subcellular distribution of these proteins and cause cell death, thus suggesting that these two proteins act in concert in pathways that regulate cell death (Stabler et al., 1999). PSEN2 and calmyrin mutually regulate each other; calmyrin regulates the PSEN2 function when it detects changes in calcium homeostasis, on the other hand, PSEN2 proteins may disrupt calcium homeostasis altering the calcium binding capacity of calmyrin. Also, the overexpression of presenilins causes perturbations in calcium balance (Guo et al., 1996; Keller et al., 1998). Any imbalance in the regulation of calcium could be fatal to the cell because calcium plays a central role in various cellular processes and in apoptosis (McConkey and Orrenius, 1997).

Normally, APP, β-and γ-secretases and phosphatidylinositol 4, 5-bisphosphate (PIP2), which is an signaling lipid moiety for endocytosis, are located on the lipid rafts. Also, It has been shown that endocytotic invagination of the membrane causes smaller lipid rafts to fuse to form larger rafts where APP, β, and γ secretases come together, this combination brings APP, β, and γ secretases in close proximity to one another causing APP cleavage thus, inducing the amyloidogenic pathway in AD (Su et al., 2010). The myristoylated alanine-rich C kinase substrate (MARCKS) usually binds to the membranes to shield PIP2 from taking a part in endocytosis. This process halts endocytosis, thus reducing the Aβ production (van Rheenen et al., 2005). Phosphorylation of MARCKS by protein kinase C (PKC) or its interaction with Ca2 + leads to its release from the membrane into the cytoplasm, thus releasing PIP2 and promoting endocytosis again, and subsequent generation of Aβ40 and Aβ42 peptides in AD (Arbuzova et al., 2002). Thus, MARCKS provides a novel therapeutic option for reducing the generation of Aβ40 and Aβ42 peptides by regulating the pathway of endocytosis.

### Ubiquitination

Ubiquitin is a greatly conserved 8.6 kDa regulatory protein composed of 76 amino acids found in almost all tissues of eukaryotes encoded by UBB, UBC, UBA52, and RPS27A genes (Kimura and Tanaka, 2010). Ubiquitination interchangeable with ubiquitylation is the addition of ubiquitin to a substrate protein. Ubiquitination is known to mark proteins for degradation; it can affect their function and alter protein sub-cellular localization as well (Mukhopadhyay and Riezman, 2007). Ubiquitination is regulated by three main enzymes viz (E1) Ubiquitin activating enzymes, (E2) Ubiquitin conjugating enzymes and (E3) Ubiquitin ligase. Ubiquitination can take place by either the addition of a single ubiquitin protein (mono-ubiquitination) or a chain of ubiquitin proteins (Polyubiquitination) (Mukhopadhyay and Riezman, 2007). These modifications generally occur at the side chain of lysine residues or the N-terminal methionine, although lately cysteine, serine and threonine residues have also been recognized as places for ubiquitination (McClellan et al., 2019). The site, length, and attachment of these ubiquitin proteins help to determine the fate and stability of a substrate (Ramesh et al., 2020). As EI, E2, and E3 enzymes help in the attachment of ubiquitin proteins, the deubiquitinases (DUBs) detach ubiquitin proteins from the substrates (Schmidt et al., 2021). Interestingly, Ubiquitin, protein can itself be post-translationally modified with acetylation and phosphorylation for enhanced diversity and regulation. The ubiquitin proteasomal degradation pathway is one of the major routes responsible for the clearance of misfolded proteins to maintain protein homeostasis. Any perturbation of the ubiquitination degradation pathway leads to toxic aggregation of species promoting the onset of various neurodegenerative diseases including Alzheimer’s (Zhang et al., 2017). AD is mainly caused by the unusual accumulation of misfolded proteins and peptides which result in the formation of amyloid plaques and NFTs, respectively. APP, BACE1 and tau proteins are the major targets of abnormal modification by ubiquitination (Perluigi et al., 2016). β-secretase/BACE1 leads to APP cleavage in the amyloidogenic pathway generating Aβ. The therapeutic inhibition/regulation of β-secretase would therefore reduce the production of all forms of beta-amyloid including the pathogenic Aβ42 (Vassar et al., 2009). The regulation of BACE1 level is done by the ubiquitination and proteasome degradation system. Tau is a protein is also rich in lys residues thus has high susceptibility toward ubiquitination. The central role of tau ubiquitination is to regulate tau clearance by proteasomal or lysosomal autophagy system (Gonzalez-Santamarta et al., 2020).

**FIGURE 5:**
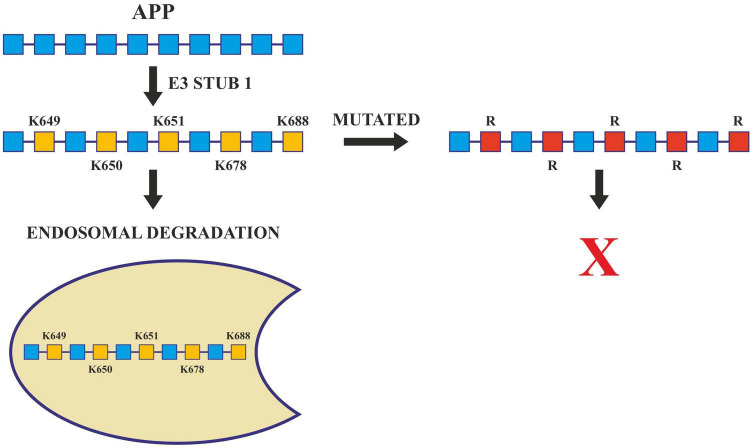
Ubiquitination in Alzheimer’s disease. Ubiquitination of APP by E3 ligase (Stub1) targets it to endosomal degradation. Mutations at lysine residues inhibit the ubiquitination and endosomal degradation of APP increasing the amyloid burden.

Further, the presenilin (PSEN) proteins are known to play an essential role in AD pathogenesis by mediating the intramembranous cleavage of APP generating (Aβ) (Oikawa and Walter, 2019). In order to sort APP into the endosome and allow their processing by PSEN, ubiquitination of its lysine residues present in its cytosolic domain is carried out by E3 ligases. These ligases are active in Alzheimer’s and they ubiquitinate at Lys 649/650/651/678 of ACR. Any mutation that changes these lysine residues to arginine hinders the ubiquitination of APP and increases Aβ40 levels (Williamson et al., 2017; Figure 5). Apart from this, Lys 203 and Lys 382 of BACE1 are also important ubiquitination sites for degradation. Mutations at these sites also disrupt the degradation of BACE1, thus increasing the production of Aβ (Wang et al., 2012). BACE1 is ubiquitinated by an E3 ligase known as FbX2 *via* trp280 which causes it to degrade through the proteasome pathway. In turn, the expression level of FbX2 gets affected by PGC-1α [Peroxisome proliferator-activated receptor gamma (PPARγ) coactivator-1α] which is known to promote the degradation of BACE 1 through the ubiquitin degradation system (Ramesh et al., 2020). The AD brain has altered expression levels of both FbX2 and PGC-1α and any supplementation of FbX2 externally reduces the levels of BACE1 and also improves synaptic function (Gong et al., 2010). This suggests that FbX2 has a role in the reduction of Aβ levels.

The ubiquitination of tau is also an important factor to study AD pathology. NFTs and paired helical filaments (PHFs) which were derived from the Alzheimer’s affected brain at an early stage, depicted that tau is hyperphosphorylated and ubiquitinated at lys-6,11, and 48. These residues thus have a significant role in AD pathogenesis. While lys48 linked polyubiquitination targets tau to proteasomal degradation, lys6 linked polyubiquitination prevents its degradation and thus hinders the clearance of PHFs (Mayeux and Stern, 2012). As already known that dUbs are the species that mediate deubiquitination, the only dUb that has been reported to target tau is the Otub1, a cysteine protease. Otub1 is reported to prevent the degradation of tau by removing Lys48 polyubiquitin chains from the endogenous tau. This removal of the Lys 48 polyubiquitin chain prevents degradation of tau in primary neurons derived from transgenic mouse models, which suggests that Otub 1 has a necessary role in regulating tau ubiquitination (Juang et al., 2012).

#### SUMOylation

SUMOylation, a reversible post-translational protein modification, occurs by the binding of an 11KDa, Small Ubiquitin-like Modifier (SUMO) peptide to the lysine residues of target proteins. SUMOylation helps in the normal functioning of proteins by regulating the transactivation of transcription factors, localization of proteins and protein-protein interactions to subcellular regions (Zhang and Sarge, 2008; Feligioni et al., 2015). Similar to the ubiquitination pathway, the SUMOylation process also requires a SUMO-EI activating enzyme, a SUMO-E2 conjugating enzyme and a SUMO-E3 ligase to complete the cycle. Various SUMO paralogues (SUMO-1,2,3,4, and 5) get expressed in a tissue specific manner. The subtypes SUMO2 and SUMO3 share sequence homology of around 95% therefore, they are more commonly called SUMO2/3. SUMO1 and SUMO2/3 are predominantly expressed in brains, SUMO4 in lymph nodes, spleen and kidney while SUMO5 is expressed in the testis (Liang et al., 2016). In AD patients, the expression of SUMO-related proteins are altered, for example the post-mortem brain sections taken from Alzheimer’s patients showed enhanced SUMO3 labeling in the hippocampal region (learning and memory) (Li et al., 2003). SENP3 (a SUMO-specific proteases 3), which takes part in the maturation of native SUMO as well as de-SUMOylation process has been reported to get down-regulated in the inferior parietal lobes of sporadic Alzheimer’s patients (Weeraratna et al., 2007). In addition, the proteins like tau, AβPP, GSK3β, BACE1, and JNK which are involved in AD are SUMO targets (Feligioni and Nisticò, 2013). As already known the abnormal intracellular accumulation is an important hallmark in the progression of AD and is an important target for SUMOylation studies. SUMO1 modification of tau happens on K340, through which tau binds with microtubules (Dorval and Fraser, 2006). SUMOylation and phosphorylation of tau are known to stimulate each other reciprocally. Increased SUMOylation enhances phosphorylation and vice-versa. The SUMOylated/Phosphorylated tau does not bind to tubulin thus making it unable to promote microtubule assembly, and it also removes normal tau from the microtubule assembly, which therefore serves as a template for the transition of normal tau into a misfolded protein (Luo H.B. et al., 2014). SUMOylation of tau also leads to increased formation of NFTs by either competing with ubiquitination or by enhancing the aggregation of tau (Luo Y. et al., 2014).

**FIGURE 6:**
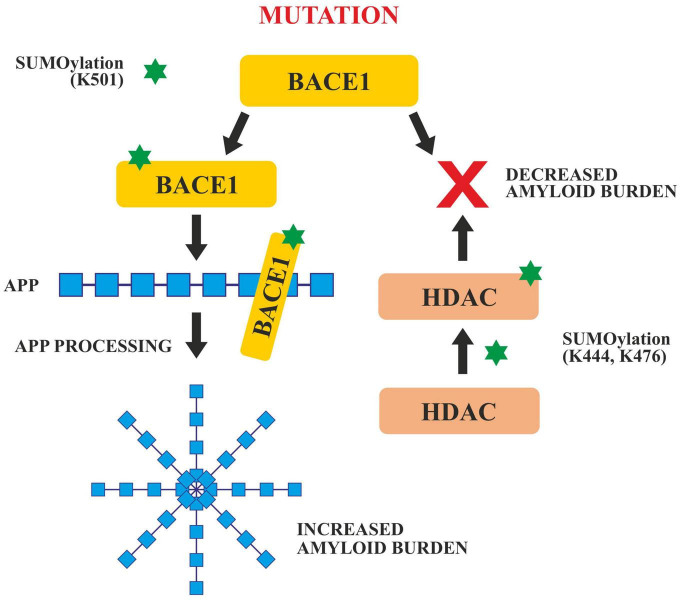
SUMOylation of BACE1 and HDAC. SUMOylation at Lys 501 enhances the activity of BACE1 which enhances APP cleavage and increases amyloid burden. Any mutation in the BACE 1 SUMOylation site decreases the amyloid burden. SUMOylation of HDAC at Lys 444 and Lys 476 also decreases the amyloid burden.

In APP processing, SUMOylation helps in its trafficking, its modulation and finally in its amyloidogenic processing. Both SUMO1 and SUMO2, SUMOylates APP *in vitro* on lysines 587 and 595 which reduces Aβ levels in Hela cells overexpressing APP (Martins et al., 2016). Apart from tau and APP, BACE 1 SUMOylation also has a significant role to play in AD pathogenesis. BACE 1, cleaves APP during late or early endosome for Aβ generation. SUMOylation of BACE1 at Lys 501, increases the stability and enhances its protease activity resulting in the processing of APP and ultimately excessive Aβ production. Mutation at this SUMOylation site (lys501) causes BACE1 to degrade which confirms that lys501 is important in stabilizing BACE1 upon SUMOylation (Ramesh et al., 2020; Figure 6).

Histone deacetylases play critical roles in the modulation of various cellular processes like chromatin remodeling, DNA repair and transcription (Seto and Yoshida, 2014). SUMOylation of HDACs at lys444 and lys476 decrease the amyloid burden (Figure 5). HDACs particularly HDAC1 maintains genomic integrity in cultured neurons and the mouse brain (Pao et al., 2020). Co-localization studies done *in vivo* and *in vitro*, have revealed that the SUMOylation of HDAC1 in the hippocampal CA2 region gets increased in the presence of PIAS1(Protein inhibitor of STAT1). HDAC1 SUMOylation also gets enhanced when Aβ is administered directly in the rat hippocampus (Tao et al., 2017). Furthermore, it has been reported that the Corticotropin Releasing Factor (CRF), Insulin like Growth Factor (IGF-1) and Brain Derived Neurotropic Factor (BDNF) increased the SUMOylation in the CA2 region of HDAC1 of the rat brain. SUMOylation of HDAC1 results in the suppression of HDAC1 and CREB (Cyclic AMP Responsive Element Binding). CREB then binds to the region of the promoter of Mcl-2 (a family of proteins having a role in apoptosis) and enhances its expression. HDAC1 SUMOylation promotes cellular apoptosis, enhances the amyloid burden and relieves memory and synaptic deficits in PSEN/APP mice (Tao et al., 2017).

### Deamidation

Deamidation is a spontaneous and non-enzymatic post translational modification which takes place at the side chains of asparagine and glutamine (Dunkelberger et al., 2012). Protein and peptides can undergo deamidation of glutaminyl (Gln) and asparaginyl (Asn) to produce glutamyl and aspartyl residues which cause changes in the structure of the protein because of the addition of negative charge. It influences protein stability, structure, folding, and aggregation. Studies done *in vivo* and *in vitro* have shown that deamidation rates are dependent upon the pH, primary sequence, and three dimensional structure of the protein, buffer ions, ionic strength, and temperature etc., (Robinson and Robinson, 2001). Although deamidation of proteins has been found to be involved in the process of amyloid formation, however, the role of deamidation in the formation of Alzheimer’s plaques is still not very well understood One hypothesis is that deamidation can contribute to the development of plaque through the isomerization of Asp to iso-Asp that might prevent the damaged peptides to degrade properly (Dunkelberger et al., 2012). As an example, the Tottori Japanese mutation (Asn-7) and the Lowa mutation (Asn-23) are two common Aβ mutations that have been found to deaminate to iso-Asp in AD patients (Wakutani et al., 2004). Under physiological conditions, the process of deamidation takes place through the formation of a 5-membered succinimide ring (intermediate) facilitated by the protein L-isoaspartate-*O*-methyltransferase (PIMT), which can restore iso-aspartate to succinimide by transferring methyl from S-adenosyl-methionine (SAM) to the side chain of iso-Asp. These 5-membered rings then hydrolyze into aspartic or iso-aspartic acid (from asparagine) or glutamic or iso-glutamic acid (from glutamine) (Dunkelberger et al., 2012). Deamidation produces L-forms of the products, but they also have the potential to racemize to D-forms through the succinimide intermediate. The ratio of normal to iso-products is 1:3 (Dunkelberger et al., 2012). This isomerization increases the length of the backbone by a CH2 group resulting in the addition of a rotable bond in the backbone of the peptide (Dunkelberger et al., 2012) which therefore, causes serious structural changes leading to the augmentation of Alzheimer’s pathogenesis (Roher et al., 1993). It has been shown that different Aβ biochemical pools have different amounts of N-terminus Isomerization. The membrane fractions and insoluble plaques in AD brains have nearly 85% isomerized Aβ_1–15_, whereas the soluble and vesicular portions have lower isomerization percentage [42 (Bronisz et al., 2014)]. Moreover, on deamidation some otherwise non-amyloidogenic proteins have been reported to convert to amyloidogenic which also leads to Alzheimer’s disease (Nilsson et al., 2002).

### Oxidative/nitrosative stress

The imbalance between the creation of reactive oxygen/nitrogen species (ROS/RNS) and the ability of the cell’s antioxidant defense to neutralize them is known as oxidative stress. The brain requires a lot of oxygen to function correctly and is highly vulnerable to the action of ROS. Furthermore, the brain is abundant in polyunsaturated fatty acids (PUFAs) that are at risk of peroxidation or free radical attack. Moreover, there is a high quantity of iron, a powerful ROS catalyst, and other antioxidant molecules present in the brain. The etiology of several neurodegenerative disorders, including Alzheimer’s disease, has been linked to an increase in oxidative stress. The increase of Aβ peptide and hyperphosphorylated tau protein in the brain during the progression of Alzheimer’s disease has been suggested to have significant pro-oxidant effects, either directly or indirectly. Through activation of NADPH oxidase and inducible nitric oxide synthase (iNOS), amyloid-β could induce reactive oxygen species (ROS) production from cortical neurons, as a result, superoxide anion (O2^–^) and nitric oxide are formed, which leads to the production of hydrogen peroxide (H_2_O_2_) and peroxynitrite (ONOO) (Medeiros et al., 2007). Amyloid-β disrupts the mitochondrial electron transport by negatively regulating the key enzymes involved in the electron transport chain such as cytochrome C oxidase which leads to the increase in superoxide anion (O2^–^) and later on its conversion into hydrogen peroxide (H_2_O_2_) thus exacerbating oxidative stress (Yan et al., 2013). Aβ induced oxidative stress also leads to the activation of different signaling pathways, for example, p38 a member of mitogen-activated protein kinases (MAPKs) family is activated during Aβ-mediated oxidative stress (Zhu et al., 2000). When compared to control individuals, late AD patients had higher levels of several lipid peroxidation markers such as 4-hydroxyhexanal (HHE), F2-isoprostane, and F4-neuroprostane. Peroxidation of lipids harms mitochondria by impairing respiration by releasing electrophilic aldehydes (Kuhla et al., 2007). Protein oxidation indicators such as protein carbonyls and 3-nitrotyrosine (3-NT) were also shown to be higher in MCI and AD patients (Alzheimer’s Association, 2019). Significant increases in the levels of DNA oxidation marker 8-hydroxy guanosine (8-OH-dG) were seen in CSF of Alzheimer’s patients when compared with control (Lovell et al., 1999). Oxidation of different proteins in the brain alters the function of enzymes responsible for the proper neuron and glial function, for example, Glutamine synthetase and creatine kinases undergo oxidative modifications during oxidative stress that leads to excitotoxicity altering the concentrations of glutamate and decrease in energy metabolism in AD (Moreira et al., 2005). As a result of oxidative stress, Aβ, and tau have been shown to undergo a variety of changes. Tau helps to organize microtubules by actively interacting with newly generated microtubules. Modification of tau by oxidative stress leads to the disruption of microtubule organization in AD patients (Heston and White, 1978). Levels of different metal ions such as Cu^2+^ and Zn^2+^ are tightly regulated for different functions of the brain and also prevent the oxidative stress resulting from the interaction of Fe^2+^ or Cu^2+^ with oxygen to generate free radicals. In AD the homeostasis is disrupted and leads to oxidative stress (Deibel et al., 1996).

In AD patients various stress responses or antioxidant genes are activated during oxidative stress such as heme-oxygenase 1, which degrades the prooxidant heme into antioxidant biliverdin, carbon monoxide and free iron. These antioxidants play central neuro-protective roles in AD (Maines, 2000; Pini et al., 2016) Gene expression of Mn^–^, Cu-, and Zn-superoxide dismutase (Mn^–^ and Cu, Zn-SOD), catalase (CAT), glutathione peroxidase (GSH-Px), and glutathione reductase (GSSG-R) is enhanced (Aksenov et al., 1998). Superoxide dismutase (SOD) is an enzyme that catalyzes the conversion of superoxide radicals to H2O2 and oxygen (O2) as part of the body’s initial defense against ROS (Balmuş et al., 2017). In both MCI and AD patients, serum SOD activity was lower than in controls. GSH reacts with oxidized products or ROS either catalyzed by glutathione peroxidase (GPx) or on its own or leads to the formation of glutathione disulphide (GSSG). Glutathione reductase can then convert the GSSG back to reduced GSH (GR). According to various human brain studies, damaged brain areas of Alzheimer’s patients have a decreased ratio of reduced to oxidized glutathione (GSH/GSSG) (Benzi and Moretti, 1995; Li et al., 2015). α-tocopherol (Vitamin E) an endogenous antioxidant protects lipid peroxidation because of its lipophilic nature. Reduced Levels of α-tocopherol were present in the plasma of mild AD patients compared to control (Brigelius-Flohé and Traber, 1999; Baldeiras et al., 2008). Higher vitamin E levels in the blood have also been linked to a lower incidence of Alzheimer’s disease in older people (Mangialasche et al., 2010). Ascorbic acid (vitamin C) is one of the most significant water-soluble antioxidants required for the reactivation of Vitamin E, and its levels in plasma are lower in MCI and AD patients as compared to controls (Rinaldi et al., 2003). Different studies have reported that N-3 PUFA’s is having a strong neuroprotective effect in humans in the early stages of AD development, corresponding to MCI. Enriching brain cells with N-3 PUFA causes a low-level oxidative/nitrosative stress, which will boost antioxidant activities and hence have a neuro-protective impact (Barone et al., 2014). Zhang et al. have recently reported that in both *in vitro* and *in vivo* models n-3 PUFAs induces neuroprotective effects through targeting Nrf2 and upregulating heme oxygenase-1 (HO-1) (Zhang et al., 2014).

## Therapeutic prospects for the treatment of Alzheimer’s disease

Some of the approaches that can be adopted for the treatment of AD include:

1. Novel exosomes-based therapeutics (NET): Several intriguing properties of exosomes, such as immunological inertness and the natural ability of EVs to transfer cargo between the cells makes them unique natural biological vehicles to cross the biological barriers such as the blood-brain barrier (BBB), which increases their therapeutic potential exponentially for delivering drugs particularly non-coding RNAs across the brain, resulting in silencing of disease-causing genes in the neurological disorders (Familtseva et al., 2019). Further, in mouse studies, it was demonstrated that Neprilysin (NEP) deficient mice showed elevated levels of Aβ, which relates NEP to the degradation of Aβ in the brain (Farris et al., 2003). When MSC-derived exosomes with NEP activity were administered intravenously, it led to reduction in the deposition of Aβ plaques in AD mice, a promising approach in AD therapeutics (Ding et al., 2018). Apart from their role as biomarkers exosomes have also emerged as promising vehicles for pharmaceuticals and they are also reported to play a neuroprotective role in neurodegenerative illnesses and traumatic brain injuries. E.g., exosomes derived from multipluripotent mesenchymal stem cells (MP-MSCs-EXOS), support functional recovery in these illnesses by inhibiting apoptosis and neuroinflammation (Alzheimer et al., 1995). However, the lack of techniques to isolate tissue specific EVs and lack of standard methodology to isolate EVs from different biological fluids are some of the critical challenges that needs to be overcome before utilizing the properties of EVs in treating various challenging diseases. For example, BACE 1 is an important therapeutic target for lowering Aβ levels in AD by specifically silencing BACE1 using small interfering RNAs (siRNAs), significant reduction of Aβ levels were seen (Zhou Y. et al., 2020). Thus, exosome based nanocarrier systems could be employed as effective therapeutic strategies in AD.
2. CRISPR/Cas9 based gene-editing: This approach has demonstrated a great potential for the treatment of AD and other diseases by correcting specific gene sequences (DiCarlo et al., 2017; Schneller et al., 2017; German et al., 2019; Mirza and Karim, 2019; Karimian et al., 2020; Xu et al., 2020; Bhardwaj et al., 2021). In a study using CRISPR/Cas9, gene editing of endogenous APP at the extreme C-terminus inhibited interactions of APP and BAC1 within the endosomes, thereby the most important cleavage event in Aβ generation was avoided in Sporadic AD (Das et al., 2013; Sun et al., 2019). Thus, CRISPR/Cas9 technology could be used to correct increased Aβ production or mutations in APP, PSEN-1, and PSEN-2 genes, which are known to be a causative factor in familial AD, and is a promising tool and has potential for therapeutics in AD. However, further studies are required to establish off-targets and safety of this approach.
3. Nanotherapeutic approaches: Functionalized and tailored nanomaterials have been designed that have the ability to cross the BBB easily and act on the target cells and cellular components like cellular proteins, peptides, and nucleic acids (Tsou et al., 2017; Furtado et al., 2018). Interestingly, nanomaterial-based drugs are considered promising due to their small size and multidimensional therapeutic abilities, and various nano-formulations such as carbon nanotubes, quantum dots, dendrimers, fullerenes, and tailor-made gold nanoparticles help in the targeted delivery of drugs in the targeted cells in AD brain to reduce synaptic impairment (Karthivashan et al., 2018; Farheen et al., 2021; Khan et al., 2021). Interestingly, the amyloid-β-derived peptides prevented *in vitro* tau aggregation and their inhibitory effect on tau and non-toxic nature points to their therapeutic importance in overcoming AD (Hampel et al., 2015; Kametani and Hasegawa, 2018; Abeysinghe et al., 2020; Farheen et al., 2021). Nanomaterials help in hindering amyloid protein growth and its accumulation due to their highly sensitive molecular detection and identification (Elbassal et al., 2017; Li et al., 2019; Jara-Guajardo et al., 2020; Gorantla et al., 2021). Due to their ability to cross BBB, nanomaterials have been used to identify AD biomarkers, and to study the beta-amyloid protein and tau proteins pathological mechanism (Kametani and Hasegawa, 2018). Another approach in nanotherapeutics is the use of nano-phytomedicine which is the conjugation of nanoparticles and medicinal plants, or their components. The antioxidative, anti-inflammatory, anticholinesterase and, anti-amyloid properties of phytochemicals make them promising therapeutic agents (Bhattacharya et al., 2022). For example, curcumin loaded poly lactic-co-glycolic-acid (PLGA) nanoparticles system has been shown to enhance the action of curcumin and when targeted to the neuroblastoma cell line for the treatment of Alzheimer’s disease they were reported to reduce oxidative damage of the cells. Further, it has been reported that the NPs-Cur delivery system enhances the action of curcumin on several pathways implicated in the pathophysiology of AD by either inhibiting M1-microglial activation or by reducing the expression of apolipoprotein J or clusterin (Chopra et al., 2021). Therefore, nanotechnology-based approaches, with all its advantages already discussed, to target various therapeutic targets in AD including PTMs provides an excellent strategy to manage the progression and decrease the burden of AD.
4. Reactive oxygen species have been shown to be one of the primary drivers in the pathogenesis of AD. Because of the protective effects of phytochemicals against oxidative stress-induced cell apoptosis, they are being explored as potential therapeutic agents for treating or preventing neurodegenerative diseases. Phytochemicals show neuro-protective effects and due to their various effects as anti-inflammatory and antioxidative activities (Palmal et al., 2014). For example, Salidroside, one of the main bioactive ingredients of R. Crenulata, was reported to improve the proliferation and neuronal differentiation of NSCs in the hippocampus of STZ-treated rats, where it acts by scavenging ROS to protect Neural Stem Cells (NSCs) from necrosis and apoptosis (Zhang et al., 2007; Uddin et al., 2021). Another example is Curcumin, which is a polyphenolic natural compound. It has shown its beneficial therapeutic effect in AD and related conditions in various preclinical and clinical studies. The findings from these studies suggested that the curcumin treatment counteracts the deleterious effects of oxidative stress and reverses memory (Walia et al., 2021). Several other phytochemicals have been reported to be involved in slowing down the onset and progression of AD and in improving cognitive functions. However, further research is needed to bring these phytochemicals to a clinical stage, where they can be used as effective antioxidants and/or anti-inflammatory agents for the treatment of AD. Plant derived phytochemicals combined with nanotherapeutics approaches have great potential in targeted therapies for AD and are quite prnset and progressioomising.
5. Another promising therapeutic approach is using antibody-based immunotherapy against Aβ to initiate its clearance or to reduce its neurotoxicity. Recently, Aducanumab, a human monoclonal antibody, when administered intravenously into transgenic mouse models of AD, was shown to selectively target the aggregated forms of Aβ by entering the brain and binding to parenchymal Aβ thereby reducing the soluble and in-soluble forms of Aβ in a dose dependent manner (Qu et al., 2012). Therefore, monoclonal antibody based immuno therapeutics hold a great promise in treating AD, besides other novel therapeutic approaches discussed above.

## Future direction

Considering the complex nature of human health and disease, a single gene, protein, glycan or a phosphorylated site may be inadequate as a precise biomarker for diagnosis and for knowing the effective possible cure for any specific disease, so the need of the hour is to establish methods to develop PTM signatures and to develop PTM specific diagnostic and therapeutic interventions for the treatment of AD. Emerging technological approaches like metabolomics, lipidomics, and proteomics hold a great promise and intriguing role in identifying and predicting the progression of neurodegenerative diseases like AD by exploring changes occurring in cells, tissues, and biofluids; structure and function of AD-related proteins, protein-protein interactions (Schumacher-Schuh et al., 2021) and so there is a need to employ these technological approaches for reliable biomarker-based diagnostics and therapeutic interventions. Recently, Huo et al. used targeted multiomics strategy to assess blood and brain samples from different cohorts and reported different serum metabolites that play an important role in predicting cognitive decline in AD patients, as well as other brain metabolites related to neuropathological measurements (Huo et al., 2020). Multiomics profiling analysis of a group of elderly patients with normal cognition, mild cognitive impairments as well as with dementia have unraveled new pathways associated with AD pathology including homeostasis, extracellular matrix signaling as well as immune responses, which enabled researchers to define combination of molecules that could be useful in predicting cognitive impairments as well as omics signatures associated with AD pathology (Clark et al., 2021). Such approaches have made it possible to identify various transcripts and metabolites associated with metabolism of fatty acids and inflammation in AD patients showing disturbances in blood brain barrier and unusual exchange of metabolites. Moreover, new compounds related to AD pathology have been identified by lipidomic analysis in AD patients that include sphingolipids, phospholipids, and ceramides, which have been found to be linked with some neuronal deuteriations like memory impairments, hippocampus volume loss and rapid progression of AD (Han et al., 2011; Mielke et al., 2012). AD is multifactorial disease with varied parameters, of which certain belongs to metabolic changes and some are environmental as well. In order to enhance the specificity of certain biomarkers at varied geographical levels, we need to enhance the proteome analysis of AD patients with accurate and precise detections Untargeted proteome analysis is need of an hour at various AD hit geographical locations to achieve this goal of differential detections and to develop AD therapeutics. A common database generated based on proteome data from plaques or from sera of patients can not only provide an insight of whole protein compositions but also help us to differentiate the proteome of subjects having underlying disease as well giving clear image of changes associated with AD. By using TMT-LC/LC-MS/MS platforms, seven deep proteomic datasets have been developed recently, which identified > 8,000 proteins from 192 subjects (Bai et al., 2020; Higginbotham et al., 2020; Wang Z. et al., 2020; Sathe et al., 2021). These data sets of AD proteome could be used as control data for other geographical locations. After developing the data-sets from various geographical locations, we need to pool the data by high-throughput screening and develop an AI based system which can easily detect the changes in proteins associated with certain factor. So that we can easily make the datasets more reliable and the biomarkers associated with it.

To help identify new biomarkers for AD, molecular profiling which is based on integration of systems biology with high-throughput technology is the best way forward in this direction and these platforms are going to be a valuable tool in AD diagnostics. It is believed that systems biology-based approaches/platforms will help uncover the exact mechanisms involved in the pathogenesis of AD and help understand about triggering factors involved in the onset and progression of the disease (Sevigny et al., 2016). This, in turn, will enable researchers to design disease modifying therapies inhibiting key steps of pathogenesis, and thereby hindering the occurrence and progression of the disease (Hampel et al., 2015; Abeysinghe et al., 2020). Furthermore, it is hoped that recent developments in the identification of biomarkers like miRNAs in exosomes and understanding of circadian clock genes and their dysregulation at different stages will prove to be potential future generation biomarkers, which can revolutionize AD diagnostics. However, more research and validation are needed in this direction.

## Conclusion

Biomarkers play a central role in the diagnosis of any disease, but the lack of clinically relevant biomarkers that can provide information about early disease stages as well as its subsequent progression has complicated development of effective treatments for AD. Several candidates as potential biomarkers for early diagnosis of AD are being studied worldwide. We have reviewed the recent literature related to most the advanced technical developments in the identification of biomarkers, like PTM signatures, levels of miRNAs in exosomes and have highlighted the need to understand circadian clock genes and their dysregulations at different stages, which are expected to be potential new generation biomarkers that can revolutionize AD diagnostics. The main hallmarks of AD include accumulation of insoluble proteins primarily composed of amyloid-β plaques and NFTs in the brain. In this review, we have described in detail how aberrant post-translational modifications of AD-related proteins like APP, Aβ, tau, and BACE1 are involved in impairing their normal function resulting in onset and progression of AD. Finally, we also discussed recent developments and therapeutic approaches for AD based on targeted siRNA/miRNA therapeutics, exosome-based drug carriers, nanoparticle-based targeted therapies, CRISPR/Cas9 based gene-editing, monoclonal antibody-based immunotherapies, and phytochemicals which are highly promising interventions that can be employed for the management and treatment of AD in future.

## Author contributions

TM and GA conceptualized the idea, developed the contents for this review, and approved the revised final manuscript. IM, MD, and TM prepared the first draft. IM, MA, MK, UM, MG, AB, GD, BA, and AH contributed in writing different sections/sub-section. IM, MA, AB, MK, and UM contributed in the preparation of original figures and tables. TM, GA, MD, IM, MA, MK, and UM contributed in critical revision of the manuscript.

## Abbreviations

AD: Alzheimer’s disease
AGES: Advanced Glycation End Products
APP: Amyloid Precursor Protein
Aβ: Amyloid Beta
BACE: β-Site APP Cleavage Enzyme
BBB: Blood Brain Barrier
BDNF: Brain Derived Neurotropic Factor
BMAL: Brain and muscle ARNT-like
Cas9: CRISPR-associated protein 9
CLOCK: Circadian Locomotor Output Cycles Kaput Protein
CNS: Central Nervous System
CR: Circadian rhythms
CREB: Cyclic AMP Responsive Element Binding
CRF: Corticotropin Releasing Factor
CRISPR: Clustered Regularly Interspaced Short Palindromic Repeats
CRY: Cryptochrome Circadian Regulators
CSF: Cerebrospinal Fluid
ELISA: Enzyme-Linked Immunosorbent Assay
ER: Endoplasmic Reticulum
EV: Extracellular vesicles
EXOs: Exosomes
FAD: Familial Alzheimer’s disease
GSSG: Glutathione Disulphide
HATs: Histone Acetyltransferases
HDACs: Histone Deacetylases
IGF: Insulin like Growth Factor
LOD: Limit of Detection
LP-MS: liquid chromatography-mass spectroscopy
MARCKS: Myristoylated Alanine-Rich C Kinase Substrate
miRNAs: micro RNAs
MP-MSCs-EXOS: Exosomes from Multipluripotent mesenchymal cells
MTBR: Microtubule Binding Region
NCS: Neuronal Stem Cells
NEP: Neprilysin
NET: Novel exosome-based therapeutics
NFTs: Neurofibrillary Tangles
NMT: N-myristoyltransferases
PER: Period Circadian Regulators
PHFs: Paired Helical Filaments
PKA: Protein Kinase A
PSEN: Presenilin
PTMs: Post-Translational Modifications
ROS/RNS: Reactive Oxygen/Nitrogen Species
SCN: Suprachiasmatic Nucleus
SCRD: Sleep and Circadian Rhythm Dysfunction
SiRNAs: Small Interfering RNAs
SUMO: Small Ubiquitin-like Modifier.

## References

[B1] Abeysinghe A. Deshapriya R. Udawatte C. (2020). Alzheimer’s disease; a review of the pathophysiological basis and therapeutic interventions. *Life Sci.* 256:117996. 10.1016/j.lfs.2020.117996 32585249

[B2] Abou-Abbass H. Abou-El-Hassan H. Bahmad H. Zibara K. Zebian A. Youssef R. (2016). Glycosylation and other PTMs alterations in neurodegenerative diseases: Current status and future role in neurotrauma. *Electrophoresis* 37 1549–1561. 10.1002/elps.201500585 26957254 PMC4962686

[B3] Absalon S. Kochanek D. M. Raghavan V. Krichevsky A. M. (2013). MiR-26b, upregulated in Alzheimer’s disease, activates cell cycle entry, tau-phosphorylation, and apoptosis in postmitotic neurons. *J. Neurosci.* 33 14645–14659. 10.1523/JNEUROSCI.1327-13.2013 24027266 PMC3810537

[B4] Akasaka-Manya K. Manya H. (2020). The Role of APP O-glycosylation in Alzheimer’s Disease. *Biomolecules* 10:1569. 10.3390/biom10111569 33218200 PMC7699271

[B5] Akhondzadeh S. (2017). New hopes for treatment of Alzheimer’s disease. *Avicenna J. Med. Biotechnol.* 10:1. PMC5742647 29296259

[B6] Aksenov M. Y. Tucker H. M. Nair P. Aksenova M. V. Butterfield D. A. Estus S. (1998). The expression of key oxidative stress-handling genes in different brain regions in Alzheimer’s disease. *J. Mol. Neurosci.* 11 151–164. 10.1385/JMN:11:2:151 10096042

[B7] Alquezar C. Arya S. Kao A. W. (2021). Tau post-translational modifications: Dynamic transformers of tau function, degradation, and aggregation. *Front. Neurol.* 11:595532. 10.3389/fneur.2020.595532 33488497 PMC7817643

[B8] Alzheimer A. Stelzma R. A. Schnitzlein H. N. Murllagh F. R. (1995). An english I’ranslation of Alzheimer’s 1907 paper,“ijber eine eigenartige erlranliung der hirnrinde”. *Clin. Anat.* 8 429–431. 10.1002/ca.980080612 8713166

[B9] Alzheimer’s Association. (2019). 2019 Alzheimer’s disease facts and figures. *Alzheimers Dement.* 15 321–387. 10.1016/j.jalz.2019.01.010

[B10] Andrew R. J. Fernandez C. G. Stanley M. Jiang H. Nguyen P. Rice R. C. (2017). Lack of BACE1 S-palmitoylation reduces amyloid burden and mitigates memory deficits in transgenic mouse models of Alzheimer’s disease. *Proc. Natl. Acad. Sci. U.S.A.* 114 E9665–E9674. 10.1073/pnas.1708568114 29078331 PMC5692556

[B11] Arbuzova A. Schmitz A. A. Vergères G. (2002). Cross-talk unfolded: MARCKS proteins. *Biochem. J.* 362 1–12. 10.1042/bj3620001 11829734 PMC1222354

[B12] Ardito F. Giuliani M. Perrone D. Troiano G. Lo Muzio L. (2017). The crucial role of protein phosphorylation in cell signaling and its use as targeted therapy. *Int. J. Mol. Med.* 40 271–280. 10.3892/ijmm.2017.3036 28656226 PMC5500920

[B13] Asai H. Ikezu S. Tsunoda S. Medalla M. Luebke J. Haydar T. (2015). Depletion of microglia and inhibition of exosome synthesis halt tau propagation. *Nat. Neurosci.* 18 1584–1593. 10.1038/nn.4132 26436904 PMC4694577

[B14] Ashton N. Leuzy A. Karikari T. Mattsson-Carlgren N. Dodich A. Boccardi M. (2021). The validation status of blood biomarkers of amyloid and phospho-tau assessed with the 5-phase development framework for AD biomarkers. *Eur. J. Nucl. Med. Mol. Imaging* 48 2140–2156. 10.1007/s00259-021-05253-y 33677733 PMC8175325

[B15] Augustinack J. C. Schneider A. Mandelkow E.-M. Hyman B. T. (2002). Specific tau phosphorylation sites correlate with severity of neuronal cytopathology in Alzheimer’s disease. *Acta Neuropathol.* 103 26–35. 10.1007/s004010100423 11837744

[B16] Avgerinos K. I. Ferrucci L. Kapogiannis D. (2021). Effects of monoclonal antibodies against amyloid-β on clinical and biomarker outcomes and adverse event risks: A systematic review and meta-analysis of phase III RCTs in Alzheimer’s disease. *Ageing Res. Rev.* 68:101339. 10.1016/j.arr.2021.101339 33831607 PMC8161699

[B17] Bai B. Wang X. Li Y. Chen P.-C. Yu K. Dey K. K. (2020). Deep multilayer brain proteomics identifies molecular networks in Alzheimer’s disease progression. *Neuron* 105 975.–991. 10.1016/j.neuron.2019.12.015 31926610 PMC7318843

[B18] Balasubramanian S. Verner E. Buggy J. J. (2009). Isoform-specific histone deacetylase inhibitors: The next step? *Cancer Lett.* 280 211–221. 10.1016/j.canlet.2009.02.013 19289255

[B19] Baldeiras I. Santana I. Proença M. T. Garrucho M. H. Pascoal R. Rodrigues A. (2008). Peripheral oxidative damage in mild cognitive impairment and mild Alzheimer’s disease. *J. Alzheimers Dis.* 15 117–128. 10.3233/JAD-2008-15110 18780972

[B20] Balmuş I.-M. Strungaru S.-A. Ciobica A. Nicoara M.-N. Dobrin R. Plavan G. (2017). Preliminary data on the interaction between some biometals and oxidative stress status in mild cognitive impairment and Alzheimer’s disease patients. *Oxid. Med. Cell. Longev.* 2017:7156928. 10.1155/2017/7156928 28811866 PMC5546061

[B21] Bannister A. J. Kouzarides T. (2011). Regulation of chromatin by histone modifications. *Cell Res.* 21 381–395. 10.1038/cr.2011.22 21321607 PMC3193420

[B22] Barcelos N. M. Van Ness P. H. Wagner A. F. MacAvoy M. G. Mecca A. P. Anderson G. M. (2018). Guanfacine treatment for prefrontal cognitive dysfunction in older participants: A randomized clinical trial. *Neurobiol. Aging* 70 117–124. 10.1016/j.neurobiolaging.2018.05.033 30007160 PMC6503670

[B23] Barone E. Di Domenico F. Mancuso C. Butterfield D. A. (2014). The Janus face of the heme oxygenase/biliverdin reductase system in Alzheimer disease: It’s time for reconciliation. *Neurobiol. Dis.* 62 144–159. 10.1016/j.nbd.2013.09.018 24095978 PMC3877707

[B24] Bellanti F. Iannelli G. Blonda M. Tamborra R. Villani R. Romano A. (2017). Alterations of clock gene RNA expression in brain regions of a triple transgenic model of Alzheimer’s disease. *J. Alzheimers Dis.* 59 615–631. 10.3233/JAD-160942 28671110 PMC5523844

[B25] Benzi G. Moretti A. (1995). Age-and peroxidative stress-related modifications of the cerebral enzymatic activities linked to mitochondria and the glutathione system. *Free Radic. Biol. Med.* 19 77–101. 10.1016/0891-5849(94)00244-E 7635361

[B26] Beshir S. A. Aadithsoorya A. Parveen A. Goh S. S. L. Hussain N. Menon V. (2022). Aducanumab therapy to treat Alzheimer’s disease: A narrative review. *Int. J. Alzheimers Dis.* 2022:9343514. 10.1155/2022/9343514 35308835 PMC8926483

[B27] Bezprozvanny I. Mattson M. P. (2008). Neuronal calcium mishandling and the pathogenesis of Alzheimer’s disease. *Trends Neurosci.* 31 454–463. 10.1016/j.tins.2008.06.005 18675468 PMC2566585

[B28] Bhardwaj S. Kesari K. K. Rachamalla M. Mani S. Ashraf G. M. Jha S. K. (2021). CRISPR/Cas9 gene editing: New hope for Alzheimer’s disease therapeutics. *J. Adv. Res.* 10.1016/j.jare.2021.07.001 [Epub ahead of print]. PMC9481950 36100328

[B29] Bhat R. V. Berg S. Burrows J. Lindquist J. (2007). “GSK-3 inhibitors for the treatment of Alzheimer’s disease,” in *Alzheimer’s disease. topics in medicinal chemistry*, eds Lau L. F. Brodney M. A. (Berlin: Springer), 10.1007/7355_2007_015

[B30] Bhattacharya T. Soares G. A. B. E. Chopra H. Rahman M. M. Hasan Z. Swain S. S. (2022). Applications of phyto-nanotechnology for the treatment of neurodegenerative disorders. *Materials* 15:804. 10.3390/ma15030804 35160749 PMC8837051

[B31] Bhattacharyya R. Barren C. Kovacs D. M. (2013). Palmitoylation of amyloid precursor protein regulates amyloidogenic processing in lipid rafts. *J. Neurosci.* 33 11169–11183. 10.1523/JNEUROSCI.4704-12.2013 23825420 PMC3718372

[B32] Bieberich E. (2014). Synthesis, processing, and function of N-glycans in N-glycoproteins. *Adv. Neurobiol.* 9 47–70. 10.1007/978-1-4939-1154-7_3 25151374 PMC4236024

[B33] Blennow K. Hampel H. Weiner M. Zetterberg H. (2010). Cerebrospinal fluid and plasma biomarkers in Alzheimer disease. *Nat. Rev. Neurol.* 6 131–144. 10.1038/nrneurol.2010.4 20157306

[B34] Blom N. Sicheritz-Pontén T. Gupta R. Gammeltoft S. Brunak S. (2004). Prediction of post-translational glycosylation and phosphorylation of proteins from the amino acid sequence. *Proteomics* 4 1633–1649. 10.1002/pmic.200300771 15174133

[B35] Boada M. López O. Núñez L. Szczepiorkowski Z. M. Torres M. Grifols C. (2019). Plasma exchange for Alzheimer’s disease management by albumin replacement (AMBAR) trial: Study design and progress. *Alzheimers Dement.* 5 61–69. 10.1016/j.trci.2019.01.001 30859122 PMC6395854

[B36] Boccardi M. Dodich A. Albanese E. Gayet-Ageron A. Festari C. Ramusino M. (2021). The strategic biomarker roadmap for the validation of Alzheimer’s diagnostic biomarkers: Methodological update. *Eur. J. Nucl. Med. Mol. Imaging* 48 2070–2085. 10.1007/s00259-020-05120-2 33688996 PMC8175304

[B37] Bolduc D. M. Montagna D. R. Seghers M. C. Wolfe M. S. Selkoe D. J. (2016). The amyloid-beta forming tripeptide cleavage mechanism of γ-secretase. *Elife* 5:e17578. 10.7554/eLife.17578 27580372 PMC5134833

[B38] Bourré G. Cantrelle F.-X. Kamah A. Chambraud B. Landrieu I. Smet-Nocca C. (2018). Direct crosstalk between O-GlcNAcylation and phosphorylation of tau protein investigated by NMR spectroscopy. *Front. Endocrinol.* 9:595. 10.3389/fendo.2018.00595 30386294 PMC6198643

[B39] Brigelius-Flohé R. Traber M. G. (1999). Vitamin E: Function and metabolism. *FASEB J.* 13 1145–1155. 10.1096/fasebj.13.10.1145 10385606

[B40] Bronisz A. Wang Y. Nowicki M. O. Peruzzi P. Ansari K. I. Ogawa D. (2014). Extracellular vesicles modulate the glioblastoma microenvironment via a tumor suppression signaling network directed by miR-1. *Cancer Res.* 74 738–750. 10.1158/0008-5472.CAN-13-2650 24310399 PMC3928601

[B41] Bu G. (2009). Apolipoprotein E and its receptors in Alzheimer’s disease: Pathways, pathogenesis and therapy. *Nat. Rev. Neurosci.* 10 333–344. 10.1038/nrn2620 19339974 PMC2908393

[B42] BursteinA.SabbaghM.AndrewsR.ValcarceC.DunnI.AltstielL. (2018). Development of Azeliragon, an oral small molecule antagonist of the receptor for advanced glycation endproducts, for the potential slowing of loss of cognition in mild Alzheimer’s disease. *J. Prev. Alzheimers Dis.* 5 149–154. 10.14283/jpad.2018.18 29616709

[B43] Carlomagno Y. Chung D. C. Yue M. Castanedes-Casey M. Madden B. J. Dunmore J. (2017). An acetylation–phosphorylation switch that regulates tau aggregation propensity and function. *J. Biol. Chem.* 292 15277–15286. 10.1074/jbc.M117.794602 28760828 PMC5602388

[B44] Chatterjee P. Fernando M. Fernando B. Dias C. B. Shah T. Silva R. (2020). Potential of coconut oil and medium chain triglycerides in the prevention and treatment of Alzheimer’s disease. *Mech. Ageing Dev.* 186:111209. 10.1016/j.mad.2020.111209 31953123

[B45] Chatterjee S. Mizar P. Cassel R. Neidl R. Selvi B. R. Mohankrishna D. V. (2013). A novel activator of CBP/p300 acetyltransferases promotes neurogenesis and extends memory duration in adult mice. *J. Neurosci.* 33 10698–10712. 10.1523/JNEUROSCI.5772-12.2013 23804093 PMC6618502

[B46] Chen G.-F. Xu T.-H. Yan Y. Zhou Y.-R. Jiang Y. Melcher K. (2017). Amyloid beta: Structure, biology and structure-based therapeutic development. *Acta Pharmacol. Sin.* 38 1205–1235. 10.1038/aps.2017.28 28713158 PMC5589967

[B47] Chen Lf Fischle W. Verdin E. Greene W. C. (2001). Duration of nuclear NF-κB action regulated by reversible acetylation. *Science* 293 1653–1657. 10.1126/science.1062374 11533489

[B48] Cheng X. Liu Z. Liu B. Zhao T. Li Y. Alam H. B. (2015). Selective histone deacetylase 6 inhibition prolongs survival in a lethal two-hit model. *J. Surg. Res.* 197 39–44. 10.1016/j.jss.2015.02.070 25837686 PMC4606460

[B49] Cho E. Park M. (2016). Palmitoylation in Alzheimer? s disease and other neurodegenerative diseases. *Pharmacol. Res.* 111 133–151. 10.1016/j.phrs.2016.06.008 27293050

[B50] Cho S.-J. Roman G. Yeboah F. Konishi Y. (2007). The road to advanced glycation end products: A mechanistic perspective. *Curr. Med. Chem.* 14 1653–1671. 10.2174/092986707780830989 17584071

[B51] Chong F. P. Ng K. Y. Koh R. Y. Chye S. M. (2018). Tau proteins and tauopathies in Alzheimer’s disease. *Cell. Mol. Neurobiol.* 38 965–980. 10.1007/s10571-017-0574-1 29299792 PMC11481908

[B52] Chopra H. Dey P. S. Das D. Bhattacharya T. Shah M. Mubin S. (2021). Curcumin nanoparticles as promising therapeutic agents for drug targets. *Molecules* 26:4998. 10.3390/molecules26164998 34443593 PMC8402133

[B53] Chun Y. S. Kwon O.-H. Oh H. G. Kim T.-W. McIntire L. B. Park M. K. (2015b). Threonine 576 residue of amyloid-β precursor protein regulates its trafficking and processing. *Biochem. Biophys. Res. Commun.* 467 955–960. 10.1016/j.bbrc.2015.10.037 26471307

[B54] Chun Y. S. Park Y. Oh H. G. Kim T.-W. Yang H. O. Park M. K. (2015a). O-GlcNAcylation promotes non-amyloidogenic processing of amyloid-β protein precursor via inhibition of endocytosis from the plasma membrane. *J. Alzheimers Dis.* 44 261–275. 10.3233/JAD-140096 25208619

[B55] Citron M. (2010). Alzheimer’s disease: Strategies for disease modification. *Nat. Rev. Drug Discov.* 9 387–398. 10.1038/nrd2896 20431570

[B56] Clark C. Dayon L. Masoodi M. Bowman G. L. Popp J. (2021). An integrative multi-omics approach reveals new central nervous system pathway alterations in Alzheimer’s disease. *Alzheimers Res. Ther.* 13 1–19. 10.1186/s13195-021-00814-7 33794997 PMC8015070

[B57] Clausen H. Bennett E. P. (1996). A family of UDP-GalNAc: Polypeptide N-acetylgalactosaminyl-transferases control the initiation of mucin-type O-linked glycosylation. *Glycobiology* 6 635–646. 10.1093/glycob/6.6.635 8922959

[B58] Cooper D. Gupta V. (2020). *Lumateperone*. Tampa, FL: StatPearls. 32809679

[B59] Corder E. H. Saunders A. M. Strittmatter W. J. Schmechel D. E. Gaskell P. C. Small G. (1993). Gene dose of apolipoprotein E type 4 allele and the risk of Alzheimer’s disease in late onset families. *Science* 261 921–923. 10.1126/science.8346443 8346443

[B60] Correia A. S. Vale N. (2021). Antidepressants in Alzheimer’s disease: A focus on the role of mirtazapine. *Pharmaceuticals (Basel)* 14:930. 10.3390/ph14090930 34577630 PMC8467729

[B61] Craft S. Claxton A. Baker L. D. Hanson A. J. Cholerton B. Trittschuh E. H. (2017). Effects of regular and long-acting insulin on cognition and Alzheimer’s disease biomarkers: A pilot clinical trial. *J. Alzheimers Dis.* 57 1325–1334. 10.3233/JAD-161256 28372335 PMC5409050

[B62] Cruz J. C. Tseng H.-C. Goldman J. A. Shih H. Tsai L.-H. (2003). Aberrant Cdk5 activation by p25 triggers pathological events leading to neurodegeneration and neurofibrillary tangles. *Neuron* 40 471–483. 10.1016/S0896-6273(03)00627-5 14642273

[B63] Cummings J. Lee G. Ritter A. Zhong K. (2018). Alzheimer’s disease drug development pipeline: 2018. *Alzheimers Dement.* 4 195–214. 10.1016/j.trci.2018.03.009 29955663 PMC6021548

[B64] Cummings J. Lee G. Ritter A. Sabbagh M. Zhong K. (2020). Alzheimer’s disease drug development pipeline: 2020. *Alzheimers Dement.* 6:e12050. 10.1002/trc2.12050 32695874 PMC7364858

[B65] Cummings J. Lee G. Ritter A. Sabbagh M. Zhong K. Research D. T. (2019). Alzheimer’s disease drug development pipeline: 2019. *Alzheimers Dement.* 5 272–293. 10.1016/j.trci.2019.05.008 31334330 PMC6617248

[B66] d’Abramo C. D’adamio L. Giliberto L. (2020). Significance of blood and cerebrospinal fluid biomarkers for Alzheimer’s disease: Sensitivity, specificity and potential for clinical use. *J. Pers. Med.* 10:116. 10.3390/jpm10030116 32911755 PMC7565390

[B67] Das U. Scott D. A. Ganguly A. Koo E. H. Tang Y. Roy S. (2013). Activity-induced convergence of APP and BACE-1 in acidic microdomains via an endocytosis-dependent pathway. *Neuron* 79 447–460. 10.1016/j.neuron.2013.05.035 23931995 PMC3741682

[B68] De Vos A. Jacobs D. Struyfs H. Fransen E. Andersson K. Portelius E. (2015). C-terminal neurogranin is increased in cerebrospinal fluid but unchanged in plasma in Alzheimer’s disease. *Alzheimers Dement.* 11 1461–1469. 10.1016/j.jalz.2015.05.012 26092348

[B69] Deibel M. Ehmann W. Markesbery W. (1996). Copper, iron, and zinc imbalances in severely degenerated brain regions in Alzheimer’s disease: Possible relation to oxidative stress. *J. Neurol. Sci.* 143 137–142. 10.1016/S0022-510X(96)00203-1 8981312

[B70] Delabio R. Rasmussen L. Mizumoto I. Viani G.-A. Chen E. Villares J. (2014). PSEN1 and PSEN2 gene expression in Alzheimer’s disease brain: A new approach. *J. Alzheimers Dis.* 42 757–760. 10.3233/JAD-140033 24927704

[B71] Deribe Y. L. Pawson T. Dikic I. (2010). Post-translational modifications in signal integration. *Nat. Struct. Mol. Biol.* 17 666–672. 10.1038/nsmb.1842 20495563

[B72] Despres C. Byrne C. Qi H. Cantrelle F.-X. Huvent I. Chambraud B. (2017). Identification of the Tau phosphorylation pattern that drives its aggregation. *Proc. Natl. Acad. Sci. U.S.A.* 114 9080–9085. 10.1073/pnas.1708448114 28784767 PMC5576827

[B73] Detke M. Lynch C. Holsinger L. Kapur S. Hennings D. Raha D. (2020). COR388 for the treatment of Alzheimer’s disease (4098). *Neurology* 94:4098.

[B74] Dewsbury L. S. Lim C. K. Steiner G. (2021). The efficacy of ketogenic therapies in the clinical management of people with neurodegenerative disease: A systematic review. *Adv. Nutr.* 12 1571–1593. 10.1093/advances/nmaa180 33621313 PMC8321843

[B75] Di Meco A. Joshi Y. B. Praticò D. (2014). Sleep deprivation impairs memory, tau metabolism, and synaptic integrity of a mouse model of Alzheimer’s disease with plaques and tangles. *Neurobiol. Aging* 35 1813–1820. 10.1016/j.neurobiolaging.2014.02.011 24629673

[B76] DiCarlo J. E. Sengillo J. D. Justus S. Cabral T. Tsang S. H. Mahajan V. B. (2017). CRISPR-cas genome surgery in ophthalmology. *Transl. Vis. Sci. Technol.* 6:13. 10.1167/tvst.6.3.13 28573077 PMC5450921

[B77] Ding M. Shen Y. Wang P. Xie Z. Xu S. Zhu Z. (2018). Exosomes isolated from human umbilical cord mesenchymal stem cells alleviate neuroinflammation and reduce amyloid-beta deposition by modulating microglial activation in Alzheimer’s disease. *Neurochem. Res.* 43 2165–2177. 10.1007/s11064-018-2641-5 30259257

[B78] Dinkins M. B. Dasgupta S. Wang G. Zhu G. Bieberich E. (2014). Exosome reduction in vivo is associated with lower amyloid plaque load in the 5XFAD mouse model of Alzheimer’s disease. *Neurobiol. Aging* 35 1792–1800. 10.1016/j.neurobiolaging.2014.02.012 24650793 PMC4035236

[B79] Dorval V. Fraser P. E. (2006). Small ubiquitin-like modifier (SUMO) modification of natively unfolded proteins tau and α-synuclein. *J. Biol. Chem.* 281 9919–9924. 10.1074/jbc.M510127200 16464864

[B80] Dunkelberger E. B. Buchanan L. E. Marek P. Cao P. Raleigh D. P. Zanni M. T. (2012). Deamidation accelerates amyloid formation and alters amylin fiber structure. *J. Am. Chem. Soc.* 134 12658–12667. 10.1021/ja3039486 22734583 PMC3410046

[B81] Ebneth A. Godemann R. Stamer K. Illenberger S. Trinczek B. Mandelkow E.-M. (1998). Overexpression of tau protein inhibits kinesin-dependent trafficking of vesicles, mitochondria, and endoplasmic reticulum: Implications for Alzheimer’s disease. *J. Cell Biol.* 143 777–794. 10.1083/jcb.143.3.777 9813097 PMC2148132

[B82] Egan M. F. Kost J. Tariot P. N. Aisen P. S. Cummings J. L. Vellas B. (2018). Randomized trial of verubecestat for mild-to-moderate Alzheimer’s disease. *N. Engl. J. Med.* 378 1691–1703. 10.1056/NEJMoa1706441 29719179 PMC6776074

[B83] Elbassal E. A. Morris C. Kent T. W. Lantz R. Ojha B. Wojcikiewicz E. P. (2017). Gold nanoparticles as a probe for amyloid-β oligomer and amyloid formation. *J. Phys. Chem. C* 121 20007–20015. 10.1021/acs.jpcc.7b05169 29276551 PMC5737797

[B84] Eldar-Finkelman H. Martinez A. (2011). GSK-3 inhibitors: Preclinical and clinical focus on CNS. *Front. Mol. Neurosci.* 4:32. 10.3389/fnmol.2011.00032 22065134 PMC3204427

[B85] Elder G. A. Gama Sosa M. A. De Gasperi R. (2010). Transgenic mouse models of Alzheimer’s disease. *Mt. Sinai. J. Med.* 77 69–81. 10.1002/msj.20159 20101721 PMC2925685

[B86] Ettcheto M. Cano A. Sanchez-López E. Verdaguer E. Folch J. Auladell C. (2021). Masitinib for the treatment of Alzheimer’s disease. *Neurodegener. Dis. Manag.* 11 263–276. 10.2217/nmt-2021-0019 34412534

[B87] Evin G. Barakat A. Masters C. L. (2010). BACE: Therapeutic target and potential biomarker for Alzheimer’s disease. *Int. J. Biochem. Cell Biol.* 42 1923–1926. 10.1016/j.biocel.2010.08.017 20817005

[B88] Familtseva A. Jeremic N. Tyagi S. C. (2019). Exosomes: Cell-created drug delivery systems. *Mol. Cell. Biochem.* 459 1–6. 10.1007/s11010-019-03545-4 31073888

[B89] Fan S.-J. Huang F.-I. Liou J.-P. Yang C.-R. (2018). The novel histone de acetylase 6 inhibitor, MPT0G211, ameliorates tau phosphorylation and cognitive deficits in an Alzheimer’s disease model. *Cell Death Dis.* 9 1–14. 10.1038/s41419-018-0688-5 29844403 PMC5974403

[B90] Farheen, Khan M. A. Ashraf G. M. Bilgrami A. L. Rizvi M. (2021). New horizons in the treatment of neurological disorders with tailorable gold nanoparticles. *Curr. Drug Metab.* 22 931–938. 10.2174/1389200222666210525123416 34036910

[B91] Farris W. Mansourian S. Chang Y. Lindsley L. Eckman E. A. Frosch M. P. (2003). Insulin-degrading enzyme regulates the levels of insulin, amyloid β-protein, and the β-amyloid precursor protein intracellular domain in vivo. *Proc. Natl. Acad. Sci. U.S.A.* 100 4162–4167. 10.1073/pnas.0230450100 12634421 PMC153065

[B92] Feligioni M. Nisticò R. (2013). SUMO: A (oxidative) stressed protein. *Neuromolecular Med.* 15 707–719. 10.1007/s12017-013-8266-6 24052421

[B93] Feligioni M. Marcelli S. Knock E. Nadeem U. Arancio O. Fraser P. E. (2015). SUMO modulation of protein aggregation and degradation. *AIMS Mol. Sci.* 2 382–410. 10.3934/molsci.2015.4.382

[B94] Fleming L. M. Johnson G. V. (1995). Modulation of the phosphorylation state of tau in situ: The roles of calcium and cyclic AMP. *Biochem. J.* 309 41–47. 10.1042/bj3090041 7619080 PMC1135797

[B95] Folch J. Petrov D. Ettcheto M. Pedros I. Abad S. Beas-Zarate C. (2015). Masitinib for the treatment of mild to moderate Alzheimer’s disease. *Expert Rev. Neurother.* 15 587–596. 10.1586/14737175.2015.1045419 25961655

[B96] Frieden C. Garai K. (2012). Structural differences between apoE3 and apoE4 may be useful in developing therapeutic agents for Alzheimer’s disease. *Proc. Natl. Acad. Sci. U.S.A.* 109 8913–8918. 10.1073/pnas.1207022109 22615372 PMC3384159

[B97] Furtado D. Björnmalm M. Ayton S. Bush A. I. Kempe K. Caruso F. (2018). Overcoming the blood–brain barrier: The role of nanomaterials in treating neurological diseases. *Adv. Mater.* 30:1801362. 10.1002/adma.201801362 30066406

[B98] Gaiottino J. Norgren N. Dobson R. Topping J. Nissim A. Malaspina A. (2013). Increased neurofilament light chain blood levels in neurodegenerative neurological diseases. *PLoS One* 8:e75091. 10.1371/journal.pone.0075091 24073237 PMC3779219

[B99] Galimberti D. Scarpini E. (2012). Progress in Alzheimer’s disease. *J. Neurol.* 259 201–211. 10.1007/s00415-011-6145-3 21706152

[B100] Galimberti D. Scarpini E. (2017). Pioglitazone for the treatment of Alzheimer’s disease. *Expert Opin. Investig. Drugs* 26 97–101. 10.1080/13543784.2017.1265504 27885860

[B101] German D. M. Mitalipov S. Mishra A. Kaul S. (2019). Therapeutic genome editing in cardiovascular diseases. *JACC: Basic Transl. Sci.* 4 122–131. 10.1016/j.jacbts.2018.11.004 30847427 PMC6390678

[B102] Giri M. Zhang M. Lü Y. (2016). Genes associated with Alzheimer’s disease: An overview and current status. *Clin. Interv. Aging* 11:665. 10.2147/CIA.S105769 27274215 PMC4876682

[B103] Goedert M. Spillantini M. Jakes R. Rutherford D. Crowther R. (1989). Multiple isoforms of human microtubule-associated protein tau: Sequences and localization in neurofibrillary tangles of Alzheimer’s disease. *Neuron* 3 519–526. 10.1016/0896-6273(89)90210-9 2484340

[B104] Gong B. Chen F. Pan Y. Arrieta-Cruz I. Yoshida Y. Haroutunian V. (2010). SCFFbx2-E3-ligase-mediated degradation of BACE1 attenuates Alzheimer’s disease amyloidosis and improves synaptic function. *Aging Cell* 9 1018–1031. 10.1111/j.1474-9726.2010.00632.x 20854419 PMC3307224

[B105] Gonzalez-Santamarta M. Quinet G. Reyes-Garau D. Sola B. Roué G. Rodriguez M. S. (2020). Resistance to the proteasome inhibitors: Lessons from multiple myeloma and mantle cell lymphoma. *Adv. Exp. Med. Biol.* 1233 153–174. 10.1007/978-3-030-38266-7_6 32274756

[B106] Gorantla N. V. Sunny L. P. Rajasekhar K. Nagaraju P. G. Cg P. P. Govindaraju T. (2021). Amyloid-β-derived peptidomimetics inhibits tau aggregation. *ACS Omega.* 6 11131–11138. 10.1021/acsomega.9b03497 34056268 PMC8153954

[B107] Graeber M. Kösel S. Egensperger R. Banati R. Müller U. Bise K. (1997). Rediscovery of the case described by alois Alzheimer in 1911: Historical, histological and molecular genetic analysis. *Neurogenetics* 1 73–80. 10.1007/s100480050011 10735278

[B108] Gräff J. Rei D. Guan J.-S. Wang W.-Y. Seo J. Hennig K. M. (2012). An epigenetic blockade of cognitive functions in the neurodegenerating brain. *Nature* 483 222–226. 10.1038/nature10849 22388814 PMC3498952

[B109] Griebel G. Stemmelin J. Lopez-Grancha M. Boulay D. Boquet G. Slowinski F. (2019). The selective GSK3 inhibitor, SAR502250, displays neuroprotective activity and attenuates behavioral impairments in models of neuropsychiatric symptoms of Alzheimer’s disease in rodents. *Sci. Rep.* 9 1–15. 10.1038/s41598-019-54557-5 31792284 PMC6888874

[B110] Guan J.-S. Haggarty S. J. Giacometti E. Dannenberg J.-H. Joseph N. Gao J. (2009). HDAC2 negatively regulates memory formation and synaptic plasticity. *Nature* 459 55–60. 10.1038/nature07925 19424149 PMC3498958

[B111] Guo Q. Furukawa K. Sopher B. L. Pham D. G. Xie J. Robinson N. (1996). Alzheimer’s PS-1 mutation perturbs calcium homeostasis and sensitizes PC12 cells to death induced by amyloid β-peptide. *Neuroreport* 8 379–383. 10.1097/00001756-199612200-00074 9051814

[B112] Gupta V. Laws S. M. Villemagne V. L. Ames D. Bush A. I. Ellis K. A. (2011). Plasma apolipoprotein E and Alzheimer disease risk: The AIBL study of aging. *Neurology* 76 1091–1098. 10.1212/WNL.0b013e318211c352 21422459

[B113] Hampel H. Lista S. Vanmechelen E. Zetterberg H. Giorgi F. S. Galgani A. (2020). β-Secretase1 biological markers for Alzheimer’s disease: State-of-art of validation and qualification. *Alzheimers Res. Ther.* 12 1–14. 10.1186/s13195-020-00686-3 33066807 PMC7566058

[B114] Hampel H. O’Bryant S. E. Molinuevo J. L. Zetterberg H. Masters C. L. Lista S. (2018). Blood-based biomarkers for Alzheimer disease: Mapping the road to the clinic. *Nat. Rev. Neurol.* 14 639–652. 10.1038/s41582-018-0079-7 30297701 PMC6211654

[B115] Hampel H. Schneider L. S. Giacobini E. Kivipelto M. Sindi S. Dubois B. (2015). Advances in the therapy of Alzheimer’s disease: Targeting amyloid beta and tau and perspectives for the future. *Expert Rev. Neurother.* 15 83–105. 10.1586/14737175.2015.995637 25537424

[B116] Han X. Rozen S. Boyle S. H. Hellegers C. Cheng H. Burke J. R. (2011). Metabolomics in early Alzheimer’s disease: Identification of altered plasma sphingolipidome using shotgun lipidomics. *PLoS One* 6:e21643. 10.1371/journal.pone.0021643 21779331 PMC3136924

[B117] Hanger D. P. Anderton B. H. Noble W. (2009). Tau phosphorylation: The therapeutic challenge for neurodegenerative disease. *Trends Mol. Med.* 15 112–119. 10.1016/j.molmed.2009.01.003 19246243

[B118] Hanger D. P. Byers H. L. Wray S. Leung K.-Y. Saxton M. J. Seereeram A. (2007). Novel phosphorylation sites in tau from Alzheimer brain support a role for casein kinase 1 in disease pathogenesis. *J. Biol. Chem.* 282 23645–23654. 10.1074/jbc.M703269200 17562708

[B119] Hansen D. V. Hanson J. E. Sheng M. (2018). Microglia in Alzheimer’s disease. *J. Cell Biol.* 217 459–472. 10.1083/jcb.201709069 29196460 PMC5800817

[B120] Haque R. U. Levey A. I. (2019). Alzheimer’s disease: A clinical perspective and future nonhuman primate research opportunities. *Proc. Natl. Acad. Sci. U.S.A.* 116 26224–26229. 10.1073/pnas.1912954116 31871211 PMC6936673

[B121] Hardy J. Selkoe D. J. (2002). The amyloid hypothesis of Alzheimer’s disease: Progress and problems on the road to therapeutics. *Science* 297 353–356. 10.1126/science.1072994 12130773

[B122] Haukedal H. Freude K. K. (2021). Implications of glycosylation in Alzheimer’s disease. *Front. Neurosci.* 14:625348. 10.3389/fnins.2020.625348 33519371 PMC7838500

[B123] Henriksen K. O’Bryant S. E. Hampel H. Trojanowski J. Q. Montine T. J. Jeromin A. (2014). The future of blood-based biomarkers for Alzheimer’s disease. *Alzheimers Dement.* 10 115–131. 10.1016/j.jalz.2013.01.013 23850333 PMC4128378

[B124] Heston L. White J. (1978). Pedigrees of 30 families with Alzheimer disease: Associations with defective organization of microfilaments and microtubules. *Behav. Genet.* 8 315–331. 10.1007/BF01067395 567976

[B125] Higginbotham L. Ping L. Dammer E. B. Duong D. M. Zhou M. Gearing M. (2020). Integrated proteomics reveals brain-based cerebrospinal fluid biomarkers in asymptomatic and symptomatic Alzheimer’s disease. *Sci. Adv.* 6:eaaz9360. 10.1126/sciadv.aaz9360 33087358 PMC7577712

[B126] Honig L. S. Vellas B. Woodward M. Boada M. Bullock R. Borrie M. (2018). Trial of solanezumab for mild dementia due to Alzheimer’s disease. *N. Engl. J. Med.* 378 321–330. 10.1056/NEJMoa1705971 29365294

[B127] Hooli B. Tanzi R. E. (2016). *The genetic basis of Alzheimer’s disease: Findings from genome-wide studies. Genomics, circuits, and pathways in clinical neuropsychiatry.* New York, NY: Elsevier, 547–571. 10.1016/B978-0-12-800105-9.00034-2

[B128] Hu G. Yang L. Cai Y. Niu F. Mezzacappa F. Callen S. (2016). Emerging roles of extracellular vesicles in neurodegenerative disorders: Focus on HIV-associated neurological complications. *Cell Death Dis.* 7:e2481. 10.1038/cddis.2016.336 27882942 PMC5260908

[B129] Hu S. Begum A. N. Jones M. R. Oh M. S. Beech W. K. Beech B. H. (2009). GSK3 inhibitors show benefits in an Alzheimer’s disease (AD) model of neurodegeneration but adverse effects in control animals. *Neurobiol. Dis.* 33 193–206. 10.1016/j.nbd.2008.10.007 19038340 PMC4313761

[B130] Huang L.-K. Chao S.-P. Hu C.-J. (2020). Clinical trials of new drugs for Alzheimer disease. *J. Biomed. Sci.* 27 1–13. 10.1186/s12929-019-0609-7 31906949 PMC6943903

[B131] Humphrey S. J. James D. E. Mann M. (2015). Protein phosphorylation: A major switch mechanism for metabolic regulation. *Trends Endocrinol. Metab.* 26 676–687. 10.1016/j.tem.2015.09.013 26498855

[B132] Hung S.-Y. Fu W.-M. (2017). Drug candidates in clinical trials for Alzheimer’s disease. *J. Biomed. Sci.* 24 1–12. 10.1186/s12929-017-0355-7 28720101 PMC5516350

[B133] Huo Z. Yu L. Yang J. Zhu Y. Bennett D. A. Zhao J. (2020). Brain and blood metabolome for Alzheimer’s dementia: Findings from a targeted metabolomics analysis. *Neurobiol. Aging* 86 123–133. 10.1016/j.neurobiolaging.2019.10.014 31785839 PMC6995427

[B134] Husain M. A. Laurent B. Plourde M. (2021). APOE and Alzheimer’s disease: From lipid transport to physiopathology and therapeutics. *Front. Neurosci.* 15:630502. 10.3389/fnins.2021.630502 33679311 PMC7925634

[B135] Ihara M. Saito S. (2020). Drug repositioning for Alzheimer’s disease: Finding hidden clues in old drugs. *J. Alzheimers Dis.* 74 1013–1028. 10.3233/JAD-200049 32144994

[B136] Iqbal K. Liu F. Gong C.-X. (2016). Tau and neurodegenerative disease: The story so far. *Nat. Rev. Neurol.* 12 15–27. 10.1038/nrneurol.2015.225 26635213

[B137] Janas A. M. Sapoń K. Janas T. Stowell M. H. Janas T. (2016). Exosomes and other extracellular vesicles in neural cells and neurodegenerative diseases. *Biochim. Biophys. Acta* 1858 1139–1151. 10.1016/j.bbamem.2016.02.011 26874206

[B138] Janeiro M. H. Ardanaz C. G. Sola-Sevilla N. Dong J. Cortés-Erice M. Solas M. (2021). Biomarkers in Alzheimer’s disease. *Adv. Lab. Med. Av. En Med. Lab.* 2 27–37. 10.1515/almed-2020-0090 PMC10197496 37359199

[B139] Janelidze S. Hertze J. Zetterberg H. Landqvist Waldö M. Santillo A. Blennow K. (2016b). Cerebrospinal fluid neurogranin and YKL-40 as biomarkers of Alzheimer’s disease. *Ann. Clin. Transl. Neurol.* 3 12–20. 10.1002/acn3.266 26783546 PMC4704480

[B140] Janelidze S. Stomrud E. Palmqvist S. Zetterberg H. Van Westen D. Jeromin A. (2016a). Plasma β-amyloid in Alzheimer’s disease and vascular disease. *Sci. Rep.* 6 1–11. 10.1038/srep26801 27241045 PMC4886210

[B141] Jara-Guajardo P. Cabrera P. Celis F. Soler M. Berlanga I. Parra-Muñoz N. (2020). Gold nanoparticles mediate improved detection of β-amyloid aggregates by fluorescence. *Nanomaterials* 10:690. 10.3390/nano10040690 32268543 PMC7221977

[B142] Juang Y.-C. Landry M.-C. Sanches M. Vittal V. Leung C. C. Ceccarelli D. F. (2012). OTUB1 co-opts Lys48-linked ubiquitin recognition to suppress E2 enzyme function. *Mol. Cell* 45 384–397. 10.1016/j.molcel.2012.01.011 22325355 PMC3306812

[B143] Kaidanovich-Beilin O. Eldar-Finkelman H. (2006). Long-term treatment with novel glycogen synthase kinase-3 inhibitor improves glucose homeostasis in ob/ob mice: Molecular characterization in liver and muscle. *J. Pharmacol. Exp. Ther.* 316 17–24. 10.1124/jpet.105.090266 16169938

[B144] Kaidanovich-Beilin O. Milman A. Weizman A. Pick C. G. Eldar-Finkelman H. (2004). Rapid antidepressive-like activity of specific glycogen synthase kinase-3 inhibitor and its effect on β-catenin in mouse hippocampus. *Biol. Psychiatry* 55 781–784. 10.1016/j.biopsych.2004.01.008 15050857

[B145] Kametani F. Hasegawa M. (2018). Reconsideration of amyloid hypothesis and tau hypothesis in Alzheimer’s disease. *Front. Neurosci.* 12:25. 10.3389/fnins.2018.00025 29440986 PMC5797629

[B146] Kang M. J. Hsu M. Krajbich I. M. Loewenstein G. McClure S. M. Wang J. T.-Y. (2009). The wick in the candle of learning: Epistemic curiosity activates reward circuitry and enhances memory. *Psychol. Sci.* 20 963–973. 10.1111/j.1467-9280.2009.02402.x 19619181

[B147] Karimian A. Gorjizadeh N. Alemi F. Asemi Z. Azizian K. Soleimanpour J. (2020). CRISPR/Cas9 novel therapeutic road for the treatment of neurodegenerative diseases. *Life Sci.* 259:118165. 10.1016/j.lfs.2020.118165 32735884

[B148] Karthivashan G. Ganesan P. Park S.-Y. Kim J.-S. Choi D.-K. (2018). Therapeutic strategies and nano-drug delivery applications in management of ageing Alzheimer’s disease. *Drug Deliv.* 25 307–320. 10.1080/10717544.2018.1428243 29350055 PMC6058502

[B149] Keller J. N. Guo Q. Holtsberg F. Bruce-Keller A. Mattson M. P. (1998). Increased sensitivity to mitochondrial toxin-induced apoptosis in neural cells expressing mutant presenilin-1 is linked to perturbed calcium homeostasis and enhanced oxyradical production. *J. Neurosci.* 18 4439–4450. 10.1523/JNEUROSCI.18-12-04439.1998 9614221 PMC6792705

[B150] Khaksarian M. Mirr I. Kordian S. Nooripour R. Ahangari N. Masjedi-Arani A. (2021). A comparison of methylphenidate (MPH) and combined methylphenidate with crocus sativus (Saffron) in the treatment of children and adolescents with ADHD: A randomized, double-blind, parallel-group, clinical trial. Iran. *J. Psychiatry Behav. Sci.* 15:e108390. 10.5812/ijpbs.108390

[B151] Khan N. H. Mir M. Ngowi E. E. Zafar U. Khakwani M. M. A. K. Khattak S. (2021). Nanomedicine: A Promising way to manage Alzheimer’s disease. *Front. Bioeng. Biotechnol.* 9:630055. 10.3389/fbioe.2021.630055 33996777 PMC8120897

[B152] Khoury R. (2022). Deuterated dextromethorphan/quinidine for agitation in Alzheimer’s disease. *Neural Regen. Res.* 17:1013. 10.4103/1673-5374.324842 34558525 PMC8552844

[B153] Khoury R. Marx C. Mirgati S. Velury D. Chakkamparambil B. Grossberg G. (2021). AVP-786 as a promising treatment option for Alzheimer’s disease including agitation. *Expert Opin. Pharmacother.* 22 783–795. 10.1080/14656566.2021.1882995 33615952

[B154] Kim C. Nam D. W. Park S. Y. Song H. Hong H. S. Boo J. H. (2013). O-linked β-N-acetylglucosaminidase inhibitor attenuates β-amyloid plaque and rescues memory impairment. *Neurobiol. Aging* 34 275–285. 10.1016/j.neurobiolaging.2012.03.001 22503002

[B155] Kim J. Basak J. M. Holtzman D. M. (2009). The role of apolipoprotein E in Alzheimer’s disease. *Neuron* 63 287–303. 10.1016/j.neuron.2009.06.026 19679070 PMC3044446

[B156] Kimura Y. Tanaka K. (2010). Regulatory mechanisms involved in the control of ubiquitin homeostasis. *J. Biochem.* 147 793–798. 10.1093/jb/mvq044 20418328

[B157] Kizuka Y. Kitazume S. Taniguchi N. (2017). N-glycan and Alzheimer’s disease. *Biochim. Biophys. Acta Gen. Subj.* 1861 2447–2454. 10.1016/j.bbagen.2017.04.012 28465241

[B158] Kizuka Y. Kitazume S. Fujinawa R. Saito T. Iwata N. Saido T. C. (2015). An aberrant sugar modification of BACE 1 blocks its lysosomal targeting in A lzheimer’s disease. *EMBO Mol. Med.* 7 175–189. 10.15252/emmm.201404438 25592972 PMC4328647

[B159] Klein G. Delmar P. Voyle N. Rehal S. Hofmann C. Abi-Saab D. (2019). Gantenerumab reduces amyloid-β plaques in patients with prodromal to moderate Alzheimer’s disease: A PET substudy interim analysis. *Alzheimers Res. Ther.* 11 1–12. 10.1186/s13195-019-0559-z 31831056 PMC6909550

[B160] Knezevic D. Mizrahi R. J. Psychiatry B. (2018). Molecular imaging of neuroinflammation in Alzheimer’s disease and mild cognitive impairment. *Prog. Neuropsychopharmacol. Biol. Psychiatry* 80 123–131. 10.1016/j.pnpbp.2017.05.007 28533150

[B161] Ko L. W. Ko E. C. Nacharaju P. Liu W.-K. Chang E. Kenessey A. (1999). An immunochemical study on tau glycation in paired helical filaments. *Brain Res.* 830 301–313. 10.1016/S0006-8993(99)01415-8 10366687

[B162] Kuhla B. Haase C. Flach K. Lüth H.-J. Arendt T. Münch G. (2007). Effect of pseudophosphorylation and cross-linking by lipid peroxidation and advanced glycation end product precursors on tau aggregation and filament formation. *J. Biol. Chem.* 282 6984–6991. 10.1074/jbc.M609521200 17082178

[B163] Kumar A. Choi K.-H. Renthal W. Tsankova N. M. Theobald D. E. Truong H.-T. (2005). Chromatin remodeling is a key mechanism underlying cocaine-induced plasticity in striatum. *Neuron* 48 303–314. 10.1016/j.neuron.2005.09.023 16242410

[B164] Kuo M. H. Allis C. D. (1998). Roles of histone acetyltransferases and deacetylases in gene regulation. *Bioessays* 20 615–626. 10.1002/(SICI)1521-1878(199808)20:8<615::AID-BIES4>3.0.CO;2-H 9780836

[B165] Lanyon-Hogg T. Faronato M. Serwa R. A. Tate E. W. (2017). Dynamic protein acylation: New substrates, mechanisms, and drug targets. *Trends Biochem. Sci.* 42 566–581. 10.1016/j.tibs.2017.04.004 28602500

[B166] Laske C. Leyhe T. Stransky E. Hoffmann N. Fallgatter A. J. Dietzsch J. (2011). Identification of a blood-based biomarker panel for classification of Alzheimer’s disease. *Int. J. Neuropsychopharmacol.* 14 1147–1155. 10.1017/S1461145711000459 21466745

[B167] Lau P. Bossers K. Janky R. S. Salta E. Frigerio C. S. Barbash S. (2013). Alteration of the micro RNA network during the progression of Alzheimer’s disease. *EMBO Mol. Med.* 5 1613–1634. 10.1002/emmm.201201974 24014289 PMC3799583

[B168] Lawlor B. Segurado R. Kennelly S. Olde Rikkert M. G. Howard R. Pasquier F. (2018). Nilvadipine in mild to moderate Alzheimer disease: A randomised controlled trial. *PLoS Med.* 15:e1002660. 10.1371/journal.pmed.1002660 30248105 PMC6152871

[B169] Lee G. Thangavel R. Sharma V. M. Litersky J. M. Bhaskar K. Fang S. M. (2004). Phosphorylation of tau by fyn: Implications for Alzheimer’s disease. *J. Neurosci.* 24 2304–2312. 10.1523/JNEUROSCI.4162-03.2004 14999081 PMC6730442

[B170] Lefebvre T. Ferreira S. Dupont-Wallois L. Bussiere T. Dupire M.-J. Delacourte A. (2003). Evidence of a balance between phosphorylation and O-GlcNAc glycosylation of Tau proteins—a role in nuclear localization. *Biochim. Biophys. Acta.* 1619 167–176. 10.1016/S0304-4165(02)00477-4 12527113

[B171] Leost M. Schultz C. Link A. Wu Y. Z. Biernat J. Mandelkow E. M. (2000). Paullones are potent inhibitors of glycogen synthase kinase-3β and cyclin-dependent kinase 5/p25. *Eur. J. Biochem.* 267 5983–5994. 10.1046/j.1432-1327.2000.01673.x 10998059

[B172] Lewczuk P. Ermann N. Andreasson U. Schultheis C. Podhorna J. Spitzer P. (2018). Plasma neurofilament light as a potential biomarker of neurodegeneration in Alzheimer’s disease. *Alzheimers Res. Ther.* 10 1–10. 10.1186/s13195-018-0404-9 30055655 PMC6064615

[B173] Li S.-Q. Yu Y. Han J.-Z. Wang D. Liu J. Qian F. (2015). Deficiency of macrophage migration inhibitory factor attenuates tau hyperphosphorylation in mouse models of Alzheimer’s disease. *J. Neuroinflammation* 12:177. 10.1186/s12974-015-0396-3 26382037 PMC4574615

[B174] Li Y. Huang H. Zhu M. Bai H. Huang X. (2021). Roles of the MYST family in the pathogenesis of Alzheimer’s disease via histone or non-histone acetylation. *Aging Dis.* 12:132. 10.14336/AD.2020.0329 33532133 PMC7801277

[B175] Li Y. Lim E. Fields T. Wu H. Xu Y. Wang Y. A. (2019). Improving sensitivity and specificity of amyloid-β peptides and tau protein detection with antibiofouling magnetic nanoparticles for liquid biopsy of Alzheimer’s disease. *ACS Biomater. Sci. Eng.* 5 3595–3605. 10.1021/acsbiomaterials.9b00086 33405741 PMC8720568

[B176] Li Y. Wang H. Wang S. Quon D. Liu Y.-W. Cordell B. (2003). Positive and negative regulation of APP amyloidogenesis by sumoylation. *Proc. Natl. Acad. Sci. U.S.A.* 100 259–264. 10.1073/pnas.0235361100 12506199 PMC140945

[B177] Liang Y.-C. Lee C.-C. Yao Y.-L. Lai C.-C. Schmitz M. L. Yang W.-M. (2016). SUMO5, a novel poly-SUMO isoform, regulates PML nuclear bodies. *Sci. Rep.* 6 1–15. 10.1038/srep26509 27211601 PMC4876461

[B178] Lin S.-Y. Hsu W.-H. Lin C.-C. Lin C.-L. Yeh H.-C. Kao C.-H. (2019). Association of transfusion with risks of dementia or Alzheimer’s disease: A population-based cohort study. *Front. Psychiatry* 10:571. 10.3389/fpsyt.2019.00571 31474887 PMC6706818

[B179] Liu C.-G. Song J. Zhang Y.-Q. Wang P.-C. (2014). MicroRNA-193b is a regulator of amyloid precursor protein in the blood and cerebrospinal fluid derived exosomal microRNA-193b is a biomarker of Alzheimer’s disease. *Mol. Med. Rep.* 10 2395–2400. 10.3892/mmr.2014.2484 25119742

[B180] Liu F. Grundke-Iqbal I. Iqbal K. Gong C. X. (2005). Contributions of protein phosphatases PP1, PP2A, PP2B and PP5 to the regulation of tau phosphorylation. *Eur. J. Neurosci.* 22 1942–1950. 10.1111/j.1460-9568.2005.04391.x 16262633

[B181] Liu F. Iqbal K. Grundke-Iqbal I. Hart G. W. Gong C.-X. (2004). O-GlcNAcylation regulates phosphorylation of tau: A mechanism involved in Alzheimer’s disease. *Proc. Natl. Acad. Sci. U.S.A.* 101 10804–10809. 10.1073/pnas.0400348101 15249677 PMC490015

[B182] Liu K. Y. Borissova A. Mahmood J. Elliott T. Knowles M. Bentham P. (2021). Pharmacological treatment trials of agitation in Alzheimer’s disease: A systematic review of clinicaltrials. Gov registered trials. *Alzheimers Dement.* 7:e12157. 10.1002/trc2.12157 33816763 PMC8010365

[B183] Liu L. Lauro B. M. He A. Lee H. Bhattarai S. Wolfe M. S. (2022). Identification of the Aβ37/42 peptide ratio in CSF as an improved Aβ biomarker for Alzheimer’s disease. *Alzheimers Dement.* 10.1002/alz.12646 [Epub ahead of print]. 35278341 PMC9464800

[B184] Liu X.-A. Das B. Chen Y. Chen Z. Avchalumov Y. Tian X. (2018). *Recent advances in Alzheimer’s drug discovery research*. Sharjah: Bentham Science, 26. 10.2174/9781681085609118070005

[B185] Lovell M. A. Gabbita S. P. Markesbery W. R. (1999). Increased DNA oxidation and decreased levels of repair products in Alzheimer’s disease ventricular CSF. *J. Neurochem.* 72 771–776. 10.1046/j.1471-4159.1999.0720771.x 9930752

[B186] Lozupone M. Berardino G. Mollica A. Sardone R. Dibello V. Zupo R. (2022). ALZT-OP1: An experimental combination regimen for the treatment of Alzheimer’s disease. *Expert Opin. Investig. Drugs* 31 759–771. 10.1080/13543784.2022.2095261 35758153

[B187] Lu X. Wang L. Yu C. Yu D. Yu G. (2015). Histone acetylation modifiers in the pathogenesis of Alzheimer’s disease. *Front. Cell. Neurosci.* 9:226. 10.3389/fncel.2015.00226 26136662 PMC4468862

[B188] Łukasik P. Baranowska-Bosiacka I. Kulczycka K. Gutowska I. (2021). Inhibitors of cyclin-dependent kinases: Types and their mechanism of action. *Int. J. Mol. Sci.* 22:2806. 10.3390/ijms22062806 33802080 PMC8001317

[B189] Luna-Munoz J. Chavez-Macias L. Garcia-Sierra F. Mena R. (2007). Earliest stages of tau conformational changes are related to the appearance of a sequence of specific phospho-dependent tau epitopes in Alzheimer’s disease 1. *J. Alzheimers Dis.* 12 365–375. 10.3233/JAD-2007-12410 18198423

[B190] Luo H.-B. Xia Y.-Y. Shu X.-J. Liu Z.-C. Feng Y. Liu X.-H. (2014). SUMOylation at K340 inhibits tau degradation through deregulating its phosphorylation and ubiquitination. *Proc. Natl. Acad. Sci. U.S.A.* 111 16586–16591. 10.1073/pnas.1417548111 25378699 PMC4246270

[B191] Luo Y. Ma B. Nussinov R. Wei G. (2014). Structural insight into tau protein’s paradox of intrinsically disordered behavior, self-acetylation activity, and aggregation. *J. Phys. Chem. Lett.* 5 3026–3031. 10.1021/jz501457f 25206938 PMC4154703

[B192] Ly P. T. Wu Y. Zou H. Wang R. Zhou W. Kinoshita A. (2012). Inhibition of GSK3β-mediated BACE1 expression reduces Alzheimer-associated phenotypes. *J. Clin. Investig.* 123 224–235. 10.1172/JCI64516 23202730 PMC3533290

[B193] Ma X. Liu L. Meng J. (2017). MicroRNA-125b promotes neurons cell apoptosis and Tau phosphorylation in Alzheimer’s disease. *Neurosci. Lett.* 661 57–62. 10.1016/j.neulet.2017.09.043 28947385

[B194] MacBean L. F. Smith A. R. Lunnon K. (2020). Exploring beyond the DNA sequence: A review of epigenomic studies of DNA and histone modifications in dementia. *Curr. Genet. Med. Rep.* 8 79–92. 10.1007/s40142-020-00190-y

[B195] Mahoney-Sanchez L. Belaidi A. A. Bush A. I. Ayton S. (2016). The complex role of apolipoprotein E in Alzheimer’s disease: An overview and update. *J. Mol. Neurosci.* 60 325–335. 10.1007/s12031-016-0839-z 27647307

[B196] Maines M. (2000). The heme oxygenase system and its functions in the brain. *Cell. Mol. Biol.* 46 573–585. 10872744

[B197] Majbour N. K. Chiasserini D. Vaikath N. N. Eusebi P. Tokuda T. Van De Berg W. (2017). Increased levels of CSF total but not oligomeric or phosphorylated forms of alpha-synuclein in patients diagnosed with probable Alzheimer’s disease. *Sci. Rep.* 7 1–8. 10.1038/srep40263 28071698 PMC5223278

[B198] Mangialasche F. Kivipelto M. Mecocci P. Rizzuto D. Palmer K. Winblad B. (2010). High plasma levels of vitamin E forms and reduced Alzheimer’s disease risk in advanced age. *J. Alzheimers Dis.* 20 1029–1037. 10.3233/JAD-2010-091450 20413888

[B199] Martins W. C. Tasca C. I. Cimarosti H. (2016). Battling Alzheimer’s disease: Targeting SUMOylation-mediated pathways. *Neurochem. Res.* 41 568–578. 10.1007/s11064-015-1681-3 26227998

[B200] Marzi S. J. Leung S. K. Ribarska T. Hannon E. Smith A. R. Pishva E. (2018). A histone acetylome-wide association study of Alzheimer’s disease identifies disease-associated H3K27ac differences in the entorhinal cortex. *Nat. Neurosci.* 21 1618–1627. 10.1038/s41593-018-0253-7 30349106

[B201] Mattsson N. Andreasson U. Zetterberg H. Blennow K. Initiative A. (2017). Association of plasma neurofilament light with neurodegeneration in patients with Alzheimer disease. *JAMA Neurol.* 74 557–566. 10.1001/jamaneurol.2016.6117 28346578 PMC5822204

[B202] Mattsson N. Smith R. Strandberg O. Palmqvist S. Schöll M. Insel P. S. (2018). Comparing 18F-AV-1451 with CSF t-tau and p-tau for diagnosis of Alzheimer disease. *Neurology.* 90 e388–e395. 10.1212/WNL.0000000000004887 29321235 PMC5791788

[B203] Mayeux R. Stern Y. (2012). Epidemiology of Alzheimer disease. *Cold Spring Harb. Perspect. Med.* 2:a006239. 10.1101/cshperspect.a006239 22908189 PMC3405821

[B204] McClellan A. J. Laugesen S. H. Ellgaard L. (2019). Cellular functions and molecular mechanisms of non-lysine ubiquitination. *Open Biol.* 9:190147. 10.1098/rsob.190147 31530095 PMC6769291

[B205] McConkey D. J. Orrenius S. (1997). The role of calcium in the regulation of apoptosis. *Biochem. Biophys. Res. Commun.* 239 357–366. 10.1006/bbrc.1997.7409 9344835

[B206] Medeiros R. Prediger R. D. Passos G. F. Pandolfo P. Duarte F. S. Franco J. L. (2007). Connecting TNF-α signaling pathways to iNOS expression in a mouse model of Alzheimer’s disease: Relevance for the behavioral and synaptic deficits induced by amyloid β protein. *J. Neurosci.* 27 5394–5404. 10.1523/JNEUROSCI.5047-06.2007 17507561 PMC6672347

[B207] Mersfelder E. L. P. (2008). *Structural and functional characterization of yeast histone acetyltransferase-1.* Columbus, OH: The Ohio State University.

[B208] Mielcarek M. Zielonka D. Carnemolla A. Marcinkowski J. T. Guidez F. J. (2015). HDAC4 as a potential therapeutic target in neurodegenerative diseases: A summary of recent achievements. *Front. Cell Neurosci.* 9:42. 10.3389/fncel.2015.00042 25759639 PMC4338808

[B209] Mielke M. M. Bandaru V. V. R. Haughey N. J. Xia J. Fried L. P. Yasar S. (2012). Serum ceramides increase the risk of Alzheimer disease: The women’s health and aging study II. *Neurology* 79 633–641. 10.1212/WNL.0b013e318264e380 22815558 PMC3414665

[B210] Mietelska-Porowska A. Wasik U. Goras M. Filipek A. Niewiadomska G. (2014). Tau protein modifications and interactions: Their role in function and dysfunction. *Int. J. Mol. Sci.* 15 4671–4713. 10.3390/ijms15034671 24646911 PMC3975420

[B211] Miki T. Yokota O. Haraguchi T. Ikeuchi T. Zhu B. Takenoshita S. (2019). Young adult-onset, very slowly progressive cognitive decline with spastic paraparesis in Alzheimer’s disease with cotton wool plaques due to a novel presenilin1 G417S mutation. *Acta Neuropathol. Commun.* 7 1–15. 10.1186/s40478-019-0672-z 30755281 PMC6371429

[B212] Miller J. L. Grant P. A. (2013). The role of DNA methylation and histone modifications in transcriptional regulation in humans. *Epigenetics* 61 289–317. 10.1007/978-94-007-4525-4_13 PMC6611551 23150256

[B213] Mirza Z. Karim S. (2019). Advancements in CRISPR/Cas9 technology—focusing on cancer therapeutics and beyond. *Semin. Cell Dev. Biol.* 96 13–21. 10.1016/j.semcdb.2019.05.026 31150758

[B214] Miura S. Yoshihisa A. Misaka T. Yamaki T. Kojima T. Toyokawa M. (2020). Amyloid precursor protein 770 is specifically expressed and released from platelets. *J. Biol. Chem.* 295 13194–13201. 10.1074/jbc.RA120.012904 32709752 PMC7504934

[B215] Moreira P. I. Honda K. Liu Q. Aliev G. Oliveira C. R. Santos M. S. (2005). Alzheimer’s disease and oxidative stress: The old problem remains unsolved. *Cent. Nerv. Syst. Agents Med. Chem.* 5 51–62. 10.2174/1568015053202714

[B216] Moremen K. W. Tiemeyer M. Nairn A. V. (2012). Vertebrate protein glycosylation: Diversity, synthesis and function. *Nat. Rev. Mol. Cell Biol.* 13 448–462. 10.1038/nrm3383 22722607 PMC3934011

[B217] Mowrer K. R. Wolfe M. S. (2008). Promotion of BACE1 mRNA alternative splicing reduces amyloid β-peptide production. *J. Biol. Chem.* 283 18694–18701. 10.1074/jbc.M801322200 18468996

[B218] Mukhopadhyay D. Riezman H. (2007). Proteasome-independent functions of ubiquitin in endocytosis and signaling. *Science* 315 201–205. 10.1126/science.1127085 17218518

[B219] Nagata T. Shinagawa S. Nakajima S. Noda Y. Mimura M. (2022). Pharmacotherapeutic combinations for the treatment of Alzheimer’s disease. *Expert Opin. Pharmacother.* 23 727–737. 10.1080/14656566.2022.2042514 35230200

[B220] Nakamura A. Kaneko N. Villemagne V. L. Kato T. Doecke J. Doré V. (2018). High performance plasma amyloid-β biomarkers for Alzheimer’s disease. *Nature* 554 249–254. 10.1038/nature25456 29420472

[B221] Neergaard J. S. Dragsbæk K. Christiansen C. Karsdal M. A. Brix S. Henriksen K. (2018). Two novel blood-based biomarker candidates measuring degradation of tau are associated with dementia: A prospective study. *PLoS One* 13:e0194802. 10.1371/journal.pone.0194802 29641555 PMC5895005

[B222] Nesterova A. P. Yuryev A. Klimov E. A. Zharkova M. Shkrob M. Ivanikova N. V. (2019). *Disease pathways: An atlas of human disease signaling pathways.* New York, NY: Elsevier.

[B223] Neumann U. Ufer M. Jacobson L. H. Rouzade-Dominguez M. L. Huledal G. Kolly C. (2018). The BACE-1 inhibitor CNP 520 for prevention trials in Alzheimer’s disease. *EMBO Mol. Med.* 10:e9316. 10.15252/emmm.201809316 30224383 PMC6220303

[B224] Nilsson M. R. Driscoll M. Raleigh D. P. (2002). Low levels of asparagine deamidation can have a dramatic effect on aggregation of amyloidogenic peptides: Implications for the study of amyloid formation. *Protein Sci.* 11 342–349. 10.1110/ps.48702 11790844 PMC2373442

[B225] Novak G. Streffer J. R. Timmers M. Henley D. Brashear H. R. Bogert J. (2020). Long-term safety and tolerability of atabecestat (JNJ-54861911), an oral BACE1 inhibitor, in early Alzheimer’s disease spectrum patients: A randomized, double-blind, placebo-controlled study and a two-period extension study. *Alzheimers Res. Ther.* 12 1–16. 10.1186/s13195-020-00614-5 32410694 PMC7227237

[B226] O’brien R. J. Wong P. C. (2011). Amyloid precursor protein processing and Alzheimer’s disease. *Annu. Rev. Neurosci.* 34:185. 10.1146/annurev-neuro-061010-113613 21456963 PMC3174086

[B227] O’Bryant S. E. Xiao G. Barber R. Reisch J. Doody R. Fairchild T. (2010). A serum protein–based algorithm for the detection of Alzheimer disease. *Arch. Neurol.* 67 1077–1081. 10.1001/archneurol.2010.215 20837851 PMC3069805

[B228] Oikawa N. Walter J. (2019). Presenilins and γ-secretase in membrane proteostasis. *Cells* 8:209. 10.3390/cells8030209 30823664 PMC6468700

[B229] Olsson B. Lautner R. Andreasson U. Öhrfelt A. Portelius E. Bjerke M. (2016). CSF and blood biomarkers for the diagnosis of Alzheimer’s disease: A systematic review and meta-analysis. *Lancet Neurol.* 15 673–684. 10.1016/S1474-4422(16)00070-3 27068280

[B230] Ovod V. Ramsey K. N. Mawuenyega K. G. Bollinger J. G. Hicks T. Schneider T. (2017). Amyloid β concentrations and stable isotope labeling kinetics of human plasma specific to central nervous system amyloidosis. *Alzheimers Dement.* 13 841–849. 10.1016/j.jalz.2017.06.2266 28734653 PMC5567785

[B231] Palmal S. Maity A. R. Singh B. K. Basu S. Jana N. R. Jana N. R. (2014). Inhibition of amyloid fibril growth and dissolution of amyloid fibrils by curcumin–gold nanoparticles. *Chem. Eur. J.* 20 6184–6191. 10.1002/chem.201400079 24691975

[B232] Pannee J. Törnqvist U. Westerlund A. Ingelsson M. Lannfelt L. Brinkmalm G. (2014). The amyloid-β degradation pattern in plasma—a possible tool for clinical trials in Alzheimer’s disease. *Neurosci. Lett.* 573 7–12. 10.1016/j.neulet.2014.04.041 24796810

[B233] Panza F. Solfrizzi V. Seripa D. Imbimbo B. P. Lozupone M. Santamato A. (2016). Tau-centric targets and drugs in clinical development for the treatment of Alzheimer’s disease. *Biomed Res. Int.* 2016:3245935. 10.1155/2016/3245935 27429978 PMC4939203

[B234] Pao P.-C. Patnaik D. Watson L. A. Gao F. Pan L. Wang J. (2020). HDAC1 modulates OGG1-initiated oxidative DNA damage repair in the aging brain and Alzheimer’s disease. *Nat. Commun.* 11 1–17. 10.1038/s41467-020-16361-y 32424276 PMC7235043

[B235] Park S.-Y. Lee Y.-H. Seong A.-R. Lee J. Jun W. Yoon H.-G. (2013). Selective inhibition of PCAF suppresses microglial-mediated β-amyloid neurotoxicity. *Int. J. Mol. Med.* 32 469–475. 10.3892/ijmm.2013.1407 23740527

[B236] Patel S. Bansoad A. V. Singh R. Khatik G. (2022). BACE1: A key regulator in Alzheimer’s disease progression and current development of its inhibitors. *Curr. Neuropharmacol.* 20 1174–1193. 10.2174/1570159X19666211201094031 34852746 PMC9886827

[B237] Perluigi M. Barone E. Di Domenico F. Butterfield D. (2016). Aberrant protein phosphorylation in Alzheimer disease brain disturbs pro-survival and cell death pathways. *Biochim. Biophys. Acta* 1862 1871–1882. 10.1016/j.bbadis.2016.07.005 27425034

[B238] Petit D. Fernández S. G. Zoltowska K. M. Enzlein T. Ryan N. S. O’Connor A. (2022). Aβ profiles generated by Alzheimer’s disease causing PSEN1 variants determine the pathogenicity of the mutation and predict age at disease onset. *Mol. Psychiatry* 27 2821–2832. 10.1038/s41380-022-01518-6 35365805 PMC9156411

[B239] Piedrahita D. Hernández I. López-Tobón A. Fedorov D. Obara B. Manjunath B. (2010). Silencing of CDK5 reduces neurofibrillary tangles in transgenic Alzheimer’s mice. *J. Neurosci.* 30 13966–13976. 10.1523/JNEUROSCI.3637-10.2010 20962218 PMC3003593

[B240] Pini L. Pievani M. Bocchetta M. Altomare D. Bosco P. Cavedo E. (2016). Brain atrophy in Alzheimer’s disease and aging. *Ageing Res. Rev.* 30 25–48. 10.1016/j.arr.2016.01.002 26827786

[B241] Porsteinsson A. P. Antonsdottir I. M. (2017). An update on the advancements in the treatment of agitation in Alzheimer’s disease. *Expert Opin. Pharmacother.* 18 611–620. 10.1080/14656566.2017.1307340 28300462

[B242] Potasiewicz A. Krawczyk M. Gzielo K. Popik P. Nikiforuk A. (2020). Positive allosteric modulators of alpha 7 nicotinic acetylcholine receptors enhance procognitive effects of conventional anti-Alzheimer drugs in scopolamine-treated rats. *Behav. Brain Res.* 385:112547. 10.1016/j.bbr.2020.112547 32087183

[B243] Qian W. Shi J. Yin X. Iqbal K. Grundke-Iqbal I. Gong C.-X. (2010). PP2A regulates tau phosphorylation directly and also indirectly via activating GSK-3β. *J. Alzheimers Dis.* 19 1221–1229. 10.3233/JAD-2010-1317 20308788

[B244] Qu Z.-Q. Zhou Y. Zeng Y.-S. Lin Y.-K. Li Y. Zhong Z.-Q. (2012). Protective effects of a Rhodiola crenulata extract and salidroside on hippocampal neurogenesis against streptozotocin-induced neural injury in the rat. *PLoS One.* 7:e29641. 10.1371/journal.pone.0029641 22235318 PMC3250459

[B245] Ramamurthy E. Welch G. Cheng J. Yuan Y. Gunsalus L. Bennett D. A. (2020). Cell type-specific histone acetylation profiling of Alzheimer’s Disease subjects and integration with genetics. *bioRxiv [Preprint]* 010330. 10.1101/2020.03.26.010330 PMC9853565 36683855

[B246] Ramesh M. Gopinath P. Govindaraju T. (2020). Role of post-translational modifications in Alzheimer’s disease. *Chembiochem* 21 1052–1079. 10.1002/cbic.201900573 31863723

[B247] Raposo G. Stahl P. D. (2019). Extracellular vesicles: A new communication paradigm? *Nat. Rev. Mol. Cell Biol.* 20 509–510. 10.1038/s41580-019-0158-7 31324871

[B248] Ravid T. Hochstrasser M. (2008). Diversity of degradation signals in the ubiquitin–proteasome system. *Nat. Rev. Mol. Cell Biol.* 9 679–689. 10.1038/nrm2468 18698327 PMC2606094

[B249] Reily C. Stewart T. J. Renfrow M. B. Novak J. (2019). Glycosylation in health and disease. *Nat. Rev. Nephrol.* 15 346–366. 10.1038/s41581-019-0129-4 30858582 PMC6590709

[B250] Resh M. D. (2016). Fatty acylation of proteins: The long and the short of it. *Prog. Lipid Res.* 63 120–131. 10.1016/j.plipres.2016.05.002 27233110 PMC4975971

[B251] Ridge P. G. Ebbert M. T. Kauwe J. (2013). Genetics of Alzheimer’s disease. *Biomed. Res. Int.* 2013:254954. 10.1155/2013/254954 23984328 PMC3741956

[B252] Rinaldi P. Polidori M. C. Metastasio A. Mariani E. Mattioli P. Cherubini A. (2003). Plasma antioxidants are similarly depleted in mild cognitive impairment and in Alzheimer’s disease. *Neurobiol. Aging.* 24 915–919. 10.1016/S0197-4580(03)00031-9 12928050

[B253] Robinson N. Robinson A. (2001). Deamidation of human proteins. *Proc. Natl. Acad. Sci. U.S.A.* 98 12409–12413. 10.1073/pnas.221463198 11606750 PMC60067

[B254] Roher A. Lowenson J. Clarke S. Wolkow C. Wang R. Cotter R. (1993). Structural alterations in the peptide backbone of beta-amyloid core protein may account for its deposition and stability in Alzheimer’s disease. *J. Biol. Chem.* 268 3072–3083. 10.1016/S0021-9258(18)53661-9 8428986

[B255] Rouaux C. Jokic N. Mbebi C. Boutillier S. Loeffler J. P. Boutillier A. L. (2003). Critical loss of CBP/p300 histone acetylase activity by caspase-6 during neurodegeneration. *EMBO J.* 22 6537–6549. 10.1093/emboj/cdg615 14657026 PMC291810

[B256] Ruthirakuhan M. Herrmann N. Andreazza A. C. Verhoeff N. P. L. Gallagher D. Black S. E. (2020). Agitation, oxidative stress, and cytokines in Alzheimer disease: Biomarker analyses from a clinical trial with nabilone for agitation. *J. Geriatr. Psychiatry Neurol.* 33 175–184. 10.1177/0891988719874118 31547752

[B257] Sacco F. Perfetto L. Castagnoli L. Cesareni G. (2012). The human phosphatase interactome: An intricate family portrait. *FEBS Lett.* 586 2732–2739. 10.1016/j.febslet.2012.05.008 22626554 PMC3437441

[B258] Salloway S. Farlow M. McDade E. Clifford D. B. Wang G. Llibre-Guerra J. J. (2021). A trial of gantenerumab or solanezumab in dominantly inherited Alzheimer’s disease. *Nat. Med.* 27 1187–1196. 10.1038/s41591-021-01369-8 34155411 PMC8988051

[B259] Salloway S. Honigberg L. A. Cho W. Ward M. Friesenhahn M. Brunstein F. (2018). Amyloid positron emission tomography and cerebrospinal fluid results from a crenezumab anti-amyloid-beta antibody double-blind, placebo-controlled, randomized phase II study in mild-to-moderate Alzheimer’s disease (BLAZE). *Alzheimers Res. Ther.* 10 1–13. 10.1186/s13195-018-0424-5 30231896 PMC6146627

[B260] Saman S. Kim W. Raya M. Visnick Y. Miro S. Saman S. (2012). Exosome-associated tau is secreted in tauopathy models and is selectively phosphorylated in cerebrospinal fluid in early Alzheimer disease. *J. Biol. Chem.* 287 3842–3849. 10.1074/jbc.M111.277061 22057275 PMC3281682

[B261] Santos A. L. Lindner A. B. (2017). Protein posttranslational modifications: Roles in aging and age-related disease. *Oxid. Med. Cell. Longev.* 2017:5716409. 10.1155/2017/5716409 28894508 PMC5574318

[B262] Sathe G. Albert M. Darrow J. Saito A. Troncoso J. Pandey A. (2021). Quantitative proteomic analysis of the frontal cortex in Alzheimer’s disease. *J. Neurochem.* 156 988–1002. 10.1111/jnc.15116 32614981 PMC7775912

[B263] Schedin-Weiss S. Winblad B. Tjernberg L. O. (2014). The role of protein glycosylation in Alzheimer disease. *FEBS J.* 281 46–62. 10.1111/febs.12590 24279329

[B264] Schmidt M. F. Gan Z. Y. Komander D. Dewson G. (2021). Ubiquitin signalling in neurodegeneration: Mechanisms and therapeutic opportunities. *Cell Death Differ.* 28 570–590. 10.1038/s41418-020-00706-7 33414510 PMC7862249

[B265] Schneller J. L. Lee C. M. Bao G. Venditti C. P. (2017). Genome editing for inborn errors of metabolism: Advancing towards the clinic. *BMC Med.* 15:43. 10.1186/s12916-017-0798-4 28238287 PMC5327528

[B266] Schumacher-Schuh A. Bieger A. Borelli W. V. Portley M. K. Awad P. S. Bandres-Ciga S. (2021). Advances in proteomic and metabolomic profiling of neurodegenerative diseases. *Front. Neurol.* 12:792227. 10.3389/fneur.2021.792227 35173667 PMC8841717

[B267] Serenó L. Coma M. Rodriguez M. Sanchez-Ferrer P. Sánchez M. B. Gich I. (2009). A novel GSK-3β inhibitor reduces Alzheimer’s pathology and rescues neuronal loss in vivo. *Neurobiol. Dis.* 35 359–367. 10.1016/j.nbd.2009.05.025 19523516

[B268] Serrano-Pozo A. Qian J. Monsell S. Betensky R. Hyman B. (2015). APOEε2 is associated with milder clinical and pathological Alzheimer’s disease. *Ann. Neurol.* 77 917–929. 10.1002/ana.24369 25623662 PMC4447539

[B269] Seto E. Yoshida M. (2014). Erasers of histone acetylation: The histone deacetylase enzymes. *Cold Spring Harb. Perspect. Biol.* 6:a018713. 10.1101/cshperspect.a018713 24691964 PMC3970420

[B270] Sevigny J. Chiao P. Bussière T. Weinreb P. H. Williams L. Maier M. (2016). The antibody aducanumab reduces Aβ plaques in Alzheimer’s disease. *Nature* 537 50–56. 10.1038/nature19323 27582220

[B271] Seymour T. Zhang J. (2021). Porphyromonas gingivalis in the pathogenesis of Alzheimer’s disease and its therapeutic target. *J Explor Res. Pharmacol.* 7 45–53. 10.14218/JERP.2021.00030

[B272] Shahbazian M. D. Grunstein M. (2007). Functions of site-specific histone acetylation and deacetylation. *Annu. Rev. Biochem.* 76 75–100. 10.1146/annurev.biochem.76.052705.162114 17362198

[B273] Sheline Y. I. Snider B. J. Beer J. C. Seok D. Fagan A. M. Suckow R. F. (2020). Effect of escitalopram dose and treatment duration on CSF Aβ levels in healthy older adults: A controlled clinical trial. *Neurology* 95 e2658–e2665. 10.1212/WNL.0000000000010725 32913021 PMC7713735

[B274] Shental-Bechor D. Levy Y. (2008). Effect of glycosylation on protein folding: A close look at thermodynamic stabilization. *Proc. Natl. Acad. Sci. U.S.A.* 105 8256–8261. 10.1073/pnas.0801340105 18550810 PMC2448824

[B275] Shkodina A. D. Tan S. C. Hasan M. M. Abdelgawad M. Chopra H. Bilal M. (2021). Roles of clock genes in the pathogenesis of Parkinson’s disease. *Ageing Res. Rev.* 74:101554. 10.1016/j.arr.2021.101554 34973458

[B276] Singh S. Li S. S.-L. (2012). Epigenetic effects of environmental chemicals bisphenol A and phthalates. *Int. J. Mol. Sci.* 13 10143–10153. 10.3390/ijms130810143 22949852 PMC3431850

[B277] Snyder H. M. Carrillo M. C. Grodstein F. Henriksen K. Jeromin A. Lovestone S. (2014). Developing novel blood-based biomarkers for Alzheimer’s disease. *Alzheimers Dement.* 10 109–114. 10.1016/j.jalz.2013.10.007 24365657 PMC4769619

[B278] Soares Martins T. Trindade D. Vaz M. Campelo I. Almeida M. Trigo G. (2021). Diagnostic and therapeutic potential of exosomes in Alzheimer’s disease. *J. Neurochem.* 156 162–181. 10.1111/jnc.15112 32618370

[B279] Soares H. D. Potter W. Z. Pickering E. Kuhn M. Immermann F. W. Shera D. M. (2012). Plasma biomarkers associated with the apolipoprotein E genotype and Alzheimer disease. *Arch. Neurol.* 69 1310–1317. 10.1001/archneurol.2012.1070 22801723 PMC3683865

[B280] Spiro R. G. (2002). Protein glycosylation: Nature, distribution, enzymatic formation, and disease implications of glycopeptide bonds. *Glycobiology* 12 43R–56R. 10.1093/glycob/12.4.43R 12042244

[B281] Stabler S. M. Ostrowski L. L. Janicki S. M. Monteiro M. J. (1999). A myristoylated calcium-binding protein that preferentially interacts with the Alzheimer’s disease presenilin 2 protein. *J. Cell Biol.* 145 1277–1292. 10.1083/jcb.145.6.1277 10366599 PMC2133148

[B282] Steiner H. Fluhrer R. Haass C. (2008). Intramembrane proteolysis by γ-secretase. *J. Biol. Chem.* 283 29627–29631. 10.1074/jbc.R800010200 18650432 PMC2662049

[B283] Stoothoff W. H. Johnson G. V. (2005). Tau phosphorylation: Physiological and pathological consequences. *Biochim. Biophys. Acta.* 1739 280–297. 10.1016/j.bbadis.2004.06.017 15615646

[B284] Struhl K. (1998). Histone acetylation and transcriptional regulatory mechanisms. *Genes Dev.* 12 599–606. 10.1101/gad.12.5.599 9499396

[B285] Su R. Han Z.-Y. Fan J.-P. Zhang Y.-L. (2010). A possible role of myristoylated alanine-rich C kinase substrate in endocytic pathway of Alzheimer’s disease. *Neurosci. Bull.* 26 338–344. 10.1007/s12264-010-0131-0 20651816 PMC5552570

[B286] Suárez-Calvet M. Kleinberger G. Araque Caballero M. Á Brendel M. Rominger A. Alcolea D. (2016). sTREM 2 cerebrospinal fluid levels are a potential biomarker for microglia activity in early-stage Alzheimer’s disease and associate with neuronal injury markers. *EMBO Mol. Med.* 8 466–476. 10.15252/emmm.201506123 26941262 PMC5120370

[B287] Sultan A. Nesslany F. Violet M. Bégard S. Loyens A. Talahari S. (2011). Nuclear tau, a key player in neuronal DNA protection. *J. Biol. Chem.* 286 4566–4575. 10.1074/jbc.M110.199976 21131359 PMC3039398

[B288] Sun J. Carlson-Stevermer J. Das U. Shen M. Delenclos M. Snead A. M. (2019). CRISPR/Cas9 editing of APP C-terminus attenuates β-cleavage and promotes α-cleavage. *Nat. Commun.* 10 1–11. 10.1038/s41467-018-07971-8 30604771 PMC6318289

[B289] Sun L. Zhou R. Yang G. Shi Y. (2017). Analysis of 138 pathogenic mutations in presenilin-1 on the in vitro production of Aβ42 and Aβ40 peptides by γ-secretase. *Proc. Natl. Acad. Sci. U.S.A.* 114 E476–E485. 10.1073/pnas.1618657114 27930341 PMC5278480

[B290] Suprun E. V. (2019). Protein post-translational modifications–A challenge for bioelectrochemistry. *Trends Anal. Chem.* 116 44–60. 10.1016/j.trac.2019.04.019

[B291] Surgucheva I. Sharov V. S. Surguchov A. (2012). γ-Synuclein: Seeding of α-synuclein aggregation and transmission between cells. *Biochemistry* 51 4743–4754. 10.1021/bi300478w 22620680

[B292] Szabo-Reed A. N. Vidoni E. Binder E. F. Burns J. Cullum C. M. Gahan W. P. (2019). Rationale and methods for a multicenter clinical trial assessing exercise and intensive vascular risk reduction in preventing dementia (rrAD Study). *Contemp. Clin. Trials* 79 44–54. 10.1016/j.cct.2019.02.007 30826452 PMC6436980

[B293] Tampi R. R. Forester B. P. Agronin M. (2021). Aducanumab: Evidence from clinical trial data and controversies. *Drugs Context* 10 2021–2027. 10.7573/dic.2021-7-3 34650610 PMC8491638

[B294] Tao C. C. Hsu W. L. Ma Y. L. Cheng S. J. Lee E. H. (2017). Epigenetic regulation of HDAC1 SUMOylation as an endogenous neuroprotection against A β toxicity in a mouse model of Alzheimer’s disease. *Cell Death Differ.* 24 597–614. 10.1038/cdd.2016.161 28186506 PMC5384022

[B295] Tate E. W. Kalesh K. A. Lanyon-Hogg T. Storck E. M. Thinon E. (2015). Global profiling of protein lipidation using chemical proteomic technologies. *Curr. Opin. Chem. Biol.* 24 48–57. 10.1016/j.cbpa.2014.10.016 25461723 PMC4319709

[B296] Thiel G. Lietz M. Hohl M. (2004). How mammalian transcriptional repressors work. *Eur. J. Biochem.* 271 2855–2862. 10.1111/j.1432-1033.2004.04174.x 15233782

[B297] Thinon E. Serwa R. A. Broncel M. Brannigan J. A. Brassat U. Wright M. H. (2014). Global profiling of co-and post-translationally N-myristoylated proteomes in human cells. *Nat. Commun.* 5 1–13. 10.1038/ncomms5919 25255805 PMC4200515

[B298] Tönnies E. Trushina E. (2017). Oxidative stress, synaptic dysfunction, and Alzheimer’s disease. *J. Alzheimers Dis.* 57 1105–1121. 10.3233/JAD-161088 28059794 PMC5409043

[B299] Tsou Y. H. Zhang X. Q. Zhu H. Syed S. Xu X. (2017). Drug delivery to the brain across the blood–brain barrier using nanomaterials. *Small* 13:1701921. 10.1002/smll.201701921 29045030

[B300] Uddin M. S. Mamun A. A. Rahman M. M. Jeandet P. Alexiou A. Behl T. (2021). Natural products for neurodegeneration: Regulating neurotrophic signals. *Oxid. Med. Cell. Longev.* 2021:8820406. 10.1155/2021/8820406 34239696 PMC8241508

[B301] Undurraga J. Sim K. Tondo L. Gorodischer A. Azua E. Tay K. H. (2019). Lithium treatment for unipolar major depressive disorder: Systematic review. *J. Psychopharmacol.* 33 167–176. 10.1177/0269881118822161 30698058

[B302] van Rheenen J. Mulugeta Achame E. Janssen H. Calafat J. Jalink K. (2005). PIP2 signaling in lipid domains: A critical re-evaluation. *EMBO J.* 24 1664–1673. 10.1038/sj.emboj.7600655 15861130 PMC1142585

[B303] Varki A. (2017). Biological roles of glycans. *Glycobiology* 27 3–49. 10.1093/glycob/cww086 27558841 PMC5884436

[B304] Vassar R. Kovacs D. M. Yan R. Wong P. C. (2009). The β-secretase enzyme BACE in health and Alzheimer’s disease: Regulation, cell biology, function, and therapeutic potential. *J. Neurosci.* 29 12787–12794. 10.1523/JNEUROSCI.3657-09.2009 19828790 PMC2879048

[B305] Vellas B. Coley N. Ousset P.-J. Berrut G. Dartigues J.-F. Dubois B. (2012). Long-term use of standardised Ginkgo biloba extract for the prevention of Alzheimer’s disease (GuidAge): A randomised placebo-controlled trial. *Lancet Neurol.* 11 851–859. 10.1016/S1474-4422(12)70206-5 22959217

[B306] Venkatesh S. Workman J. L. (2015). Histone exchange, chromatin structure and the regulation of transcription. *Nat. Rev. Mol. Cell Biol.* 16 178–189. 10.1038/nrm3941 25650798

[B307] Verkhratsky A. Zorec R. Rodríguez J. J. Parpura V. (2016). Astroglia dynamics in ageing and Alzheimer’s disease. *Curr. Opin. Pharmacol.* 26 74–79. 10.1016/j.coph.2015.09.011 26515274

[B308] Vetrivel K. S. Meckler X. Chen Y. Nguyen P. D. Seidah N. G. Vassar R. (2009). Alzheimer disease Aβ production in the absence of S-palmitoylation-dependent targeting of BACE1 to lipid rafts. *J. Biol. Chem.* 284 3793–3803. 10.1074/jbc.M808920200 19074428 PMC2635050

[B309] Villarreal S. Zhao F. Hyde L. A. Holder D. Forest T. Sondey M. (2017). Chronic verubecestat treatment suppresses amyloid accumulation in advanced aged Tg2576-AβPP swe mice without inducing microhemorrhage. *J. Alzheimers Dis.* 59 1393–1413. 10.3233/JAD-170056 28800329 PMC5611839

[B310] Volmar C.-H. Wahlestedt C. (2015). Histone deacetylases (HDACs) and brain function. *Neuroepigenetics* 1 20–27. 10.1016/j.nepig.2014.10.002

[B311] Wakabayashi T. De Strooper B. J. P. (2008). Presenilins: Members of the γ-secretase quartets, but part-time soloists too. *Physiology (Bethesda)* 23 194–204. 10.1152/physiol.00009.2008 18697993

[B312] Wakutani Y. Watanabe K. Adachi Y. Wada-Isoe K. Urakami K. Ninomiya H. (2004). Novel amyloid precursor protein gene missense mutation (D678N) in probable familial Alzheimer’s disease. *J. Neurol. Neurosurg. Psychiatry* 75 1039–1042. 10.1136/jnnp.2003.010611 15201367 PMC1739111

[B313] Walia V. Kaushik D. Mittal V. Kumar K. Verma R. Parashar J. (2021). Delineation of neuroprotective effects and possible benefits of antioxidantstherapy for the treatment of Alzheimer’s diseases by targeting mitochondrial-derived reactive oxygen species: Bench to bedside. *Mol. Neurobiol.* 59 657–680. 10.1007/s12035-021-02617-1 34751889

[B314] Wang J. Z. Xia Y. Y. Grundke-Iqbal I. Iqbal K. (2013). Abnormal hyperphosphorylation of tau: Sites, regulation, and molecular mechanism of neurofibrillary degeneration. *J. Alzheimers Dis.* 33 S123–S139. 10.3233/JAD-2012-129031 22710920

[B315] Wang R. Ying Z. Zhao J. Zhang Y. Wang R. Lu H. (2012). Lys203 and Lys382 are essential for the proteasomal degradation of BACE1. *Curr. Alzheimer Res.* 9 606–615. 10.2174/156720512800618026 22299711

[B316] Wang T. Kuang W. Chen W. Xu W. Zhang L. Li Y. (2020). A phase II randomized trial of sodium oligomannate in Alzheimer’s dementia. *Alzheimers Res. Ther.* 12 1–10. 10.1186/s13195-020-00678-3 32928279 PMC7489025

[B317] Wang W.-Y. Tan M.-S. Yu J.-T. Tan L. (2015). Role of pro-inflammatory cytokines released from microglia in Alzheimer’s disease. *Ann. Transl. Med.* 3:136. 10.3978/j.issn.2305-5839.2015.03.49 PMC4486922 26207229

[B318] Wang Z. Yu K. Tan H. Wu Z. Cho J.-H. Han X. (2020). 27-Plex tandem mass tag mass spectrometry for profiling brain proteome in Alzheimer’s disease. *Anal. Chem.* 92 7162–7170. 10.1021/acs.analchem.0c00655 32343560 PMC8176402

[B319] Watson L. S. Hamlett E. D. Stone T. D. Sims-Robinson C. (2019). Neuronally derived extracellular vesicles: An emerging tool for understanding Alzheimer’s disease. *Mol. Neurodegener.* 14 1–9. 10.1186/s13024-019-0317-5 31182115 PMC6558712

[B320] Weeraratna A. T. Kalehua A. DeLeon I. Bertak D. Maher G. Wade M. S. (2007). Alterations in immunological and neurological gene expression patterns in Alzheimer’s disease tissues. *Exp. Cell Res.* 313 450–461. 10.1016/j.yexcr.2006.10.028 17188679 PMC2565515

[B321] Wellington H. Paterson R. W. Portelius E. Törnqvist U. Magdalinou N. Fox N. C. (2016). Increased CSF neurogranin concentration is specific to Alzheimer disease. *Neurology* 86 829–835. 10.1212/WNL.0000000000002423 26826204 PMC4793782

[B322] Wessels A. M. Tariot P. N. Zimmer J. A. Selzler K. J. Bragg S. M. Andersen S. W. (2020). Efficacy and safety of lanabecestat for treatment of early and mild Alzheimer disease: The AMARANTH and DAYBREAK-ALZ randomized clinical trials. *JAMA Neurol.* 77 199–209. 10.1001/jamaneurol.2019.3988 31764959 PMC6902191

[B323] Williamson R. L. Laulagnier K. Miranda A. M. Fernandez M. A. Wolfe M. S. Sadoul R. (2017). Disruption of amyloid precursor protein ubiquitination selectively increases amyloid β (Aβ) 40 levels via presenilin 2-mediated cleavage. *J. Biol. Chem.* 292 19873–19889. 10.1074/jbc.M117.818138 29021256 PMC5712626

[B324] Wright M. H. Heal W. P. Mann D. J. Tate E. W. (2010). Protein myristoylation in health and disease. *J. Chem. Biol.* 3 19–35. 10.1007/s12154-009-0032-8 19898886 PMC2816741

[B325] Wu C.-H. (2017). The association between the use of zolpidem and the risk of Alzheimer’s disease among older people. *J. Am. Geriatr. Soc.* 65 2488–2495. 10.1111/jgs.15018 28884784

[B326] Xia D. Watanabe H. Wu B. Lee S. H. Li Y. Tsvetkov E. (2015). Presenilin-1 knockin mice reveal loss-of-function mechanism for familial Alzheimer’s disease. *Neuron* 85 967–981. 10.1016/j.neuron.2015.02.010 25741723 PMC4358812

[B327] Xiang Z. Haroutunian V. Ho L. Purohit D. Pasinetti G. M. (2006). Microglia activation in the brain as inflammatory biomarker of Alzheimer’s disease neuropathology and clinical dementia. *Dis. Markers* 22 95–102. 10.1155/2006/276239 16410654 PMC3850819

[B328] Xie L. Zhu Q. Lu J. (2022). Can we use ginkgo biloba extract to treat Alzheimer’s disease? Lessons from preclinical and clinical studies. *Cells* 11:479. 10.3390/cells11030479 35159288 PMC8833923

[B329] Xu K. Dai X.-L. Huang H.-C. Jiang Z.-F. (2011). Targeting HDACs: A promising therapy for Alzheimer’s disease. *Oxid. Med. Cell. Longev.* 2011:143269. 10.1155/2011/143269 21941604 PMC3177096

[B330] Xu S. Wilf R. Menon T. Panikker P. Sarthi J. Elefant F. (2014). Epigenetic control of learning and memory in Drosophila by Tip60 HAT action. *Genetics* 198 1571–1586. 10.1534/genetics.114.171660 25326235 PMC4256772

[B331] Xu T. Li L. Liu Y. C. Cao W. Chen J. S. Hu S. (2020). CRISPR/Cas9-related technologies in liver diseases: From feasibility to future diversity. *Int. J. Biol. Sci.* 16:2283. 10.7150/ijbs.33481 32760197 PMC7378651

[B332] Xu X.-H. Huang Y. Wang G. Chen S.-D. (2012). Metabolomics: A novel approach to identify potential diagnostic biomarkers and pathogenesis in Alzheimer’s disease. *Neurosci. Bull.* 28 641–648. 10.1007/s12264-012-1272-0 23054640 PMC5561924

[B333] Yamini P. Ray R. Chopra K. J. I. (2018). Vitamin D_3_ attenuates cognitive deficits and neuroinflammatory responses in ICV-STZ induced sporadic Alzheimer’s disease. *Inflammopharmacology* 26 39–55. 10.1007/s10787-017-0372-x 28702935

[B334] Yan M. H. Wang X. Zhu X. (2013). Mitochondrial defects and oxidative stress in Alzheimer disease and Parkinson disease. *Free Radic. Biol. Med.* 62 90–101. 10.1016/j.freeradbiomed.2012.11.014 23200807 PMC3744189

[B335] Yang C.-C. Chiu M.-J. Chen T.-F. Chang H.-L. Liu B.-H. Yang S.-Y. (2018). Assay of plasma phosphorylated tau protein (threonine 181) and total tau protein in early-stage Alzheimer’s disease. *J. Alzheimers Dis.* 61 1323–1332. 10.3233/JAD-170810 29376870

[B336] Yang L. Liu Y. Wang Y. Li J. Liu N. J. C. (2021). Azeliragon ameliorates Alzheimer’s disease via the Janus tyrosine kinase and signal transducer and activator of transcription signaling pathway. *Clinics* 76:e2348. 10.6061/clinics/2021/e2348 33681944 PMC7920406

[B337] Yiannopoulou K. G. Papageorgiou S. G. (2020). Current and future treatments in Alzheimer disease: An update. *J. Cent. Nerv. Syst. Dis.* 12:1179573520907397. 10.1177/1179573520907397 32165850 PMC7050025

[B338] Yuzwa S. A. Yadav A. K. Skorobogatko Y. Clark T. Vosseller K. Vocadlo D. J. (2011). Mapping O-GlcNAc modification sites on tau and generation of a site-specific O-GlcNAc tau antibody. *Amino Acids.* 40 857–868. 10.1007/s00726-010-0705-1 20706749

[B339] Zarini-Gakiye E. Amini J. Sanadgol N. Vaezi G. Parivar K. (2020). Recent updates in the Alzheimer’s disease etiopathology and possible treatment approaches: A narrative review of current clinical trials. *Curr. Mol. Pharmacol.* 13 273–294. 10.2174/1874467213666200422090135 32321414

[B340] Zetterberg H. Wilson D. Andreasson U. Minthon L. Blennow K. Randall J. (2013). Plasma tau levels in Alzheimer’s disease. *Alzheimers Res. Ther.* 5 1–3. 10.1186/alzrt163 23551972 PMC3707015

[B341] Zhang F. Phiel C. J. Spece L. Gurvich N. Klein P. S. (2003). Inhibitory phosphorylation of glycogen synthase kinase-3 (GSK-3) in response to lithium: Evidence for autoregulation of GSK-3. *J. Biol. Chem.* 278 33067–33077. 10.1074/jbc.M212635200 12796505

[B342] Zhang L. Yu H. Sun Y. Lin X. Chen B. Tan C. (2007). Protective effects of salidroside on hydrogen peroxide-induced apoptosis in SH-SY5Y human neuroblastoma cells. *Eur. J. Pharmacol.* 564 18–25. 10.1016/j.ejphar.2007.01.089 17349619

[B343] Zhang M. Wang S. Mao L. Leak R. K. Shi Y. Zhang W. (2014). Omega-3 fatty acids protect the brain against ischemic injury by activating Nrf2 and upregulating heme oxygenase 1. *J. Neurosci.* 34 1903–1915. 10.1523/JNEUROSCI.4043-13.2014 24478369 PMC3905150

[B344] Zhang S. Lei C. Liu P. Zhang M. Tao W. Liu H. (2015). Association between variant amyloid deposits and motor deficits in FAD-associated presenilin-1 mutations: A systematic review. *Neurosci. Biobehav. Rev.* 56 180–192. 10.1016/j.neubiorev.2015.07.003 26165445

[B345] Zhang Y. Chen X. Zhao Y. Ponnusamy M. Liu Y. (2017). The role of ubiquitin proteasomal system and autophagy-lysosome pathway in Alzheimer’s disease. *Nat. Rev. Neurosci.* 28 861–868. 10.1515/revneuro-2017-0013 28704199

[B346] Zhang Y.-Q. Sarge K. D. (2008). Sumoylation of amyloid precursor protein negatively regulates Aβ aggregate levels. *Biochem. Biophys. Res. Commun.* 374 673–678. 10.1016/j.bbrc.2008.07.109 18675254 PMC2596940

[B347] Zhao J. Liu X. Xia W. Zhang Y. Wang C. (2020). Targeting amyloidogenic processing of APP in Alzheimer’s disease. *Front. Mol. Neurosci.* 13:137. 10.3389/fnmol.2020.00137 32848600 PMC7418514

[B348] Zheng N. Wang N. Jia J.-M. (2020). Therapeutic benefit of aripiprazole-olanzapine combination in the treatment of senile Alzheimer’s disease complicated by mental disorders. *Trop. J. Pharm. Res.* 19 441–446. 10.4314/tjpr.v19i2.29

[B349] Zhou F. Yan X.-D. Wang C. He Y.-X. Li Y.-Y. Zhang J. (2020). Suvorexant ameliorates cognitive impairments and pathology in APP/PS1 transgenic mice. *Neurobiol. Aging* 91 66–75. 10.1016/j.neurobiolaging.2020.02.020 32224066

[B350] Zhou Y. Shi J. Chu D. Hu W. Guan Z. Gong C.-X. (2018). Relevance of phosphorylation and truncation of tau to the etiopathogenesis of Alzheimer’s disease. *Front. Aging Neurosci.* 10:27. 10.3389/fnagi.2018.00027 29472853 PMC5810298

[B351] Zhou Y. Zhu F. Liu Y. Zheng M. Wang Y. Zhang D. (2020). Blood-brain barrier–penetrating siRNA nanomedicine for Alzheimer’s disease therapy. *Sci. Adv.* 6:eabc7031. 10.1126/sciadv.abc7031 33036977 PMC7546706

[B352] Zhu X. Rottkamp C. A. Boux H. Takeda A. Perry G. Smith M. A. (2000). Activation of p38 kinase links tau phosphorylation, oxidative stress, and cell cycle-related events in Alzheimer disease. *J. Neuropathol. Exp. Neurol.* 59 880–888. 10.1093/jnen/59.10.880 11079778

